# General strategy for boosting the performance of speed-tunable rotary molecular motors with visible light

**DOI:** 10.1126/sciadv.adr9326

**Published:** 2025-02-19

**Authors:** Jinyu Sheng, Carlijn L. F. van Beek, Charlotte N. Stindt, Wojciech Danowski, Joanna Jankowska, Stefano Crespi, Daisy R. S. Pooler, Michiel F. Hilbers, Wybren Jan Buma, Ben L. Feringa

**Affiliations:** ^1^ Stratingh Institute for Chemistry, University of Groningen, Nijenborgh 3, 9747 AG Groningen, Netherlands.; ^2^ Institute of Science and Technology Austria, Am Campus 1, 3400 Klosterneuburg, Austria.; ^3^ Faculty of Chemistry, University of Warsaw, Pasteura 1, 02-093 Warsaw, Poland.; ^4^ Department of Chemistry - Ångström Laboratory, Uppsala University, Box 523, 751 20 Uppsala, Sweden.; ^5^ Van ‘t Hoff Institute for Molecular Sciences, University of Amsterdam, Science Park 904, 1098 XH Amsterdam, Netherlands.; ^6^ FELIX Laboratory, Radboud University, Toernooiveld 7c, 6525 ED Nijmegen, Netherlands.; ^7^ Zernike Institute for Advanced Materials, University of Groningen, Nijenborgh 4, 9747 AG Groningen, Netherlands.

## Abstract

Light-driven molecular rotary motors perform chirality-controlled unidirectional rotations fueled by light and heat. This unique function renders them appealing for the construction of dynamic molecular systems, actuating materials, and molecular machines. Achieving a combination of high photoefficiency, visible-light responsiveness, synthetic accessibility, and easy tuning of dynamic properties within a single scaffold is critical for these applications but remains a longstanding challenge. Herein, a series of highly photoefficient visible-light–responsive molecular motors (MMs), featuring various rotary speeds, was obtained by a convenient one-step formylation of their parent motors. This strategy greatly improves all aspects of the performance of MMs—red-shifted wavelengths of excitation, high photoisomerization quantum yields, and high photostationary state distributions of isomers—beyond the state-of-the-art light-responsive MM systems. The development of this late-stage functionalization strategy of MMs opens avenues for the construction of high-performance molecular machines and devices for applications in materials science and biological systems, representing a major advance in the synthetic toolbox of molecular machines.

## Teaser

Formylation improves all aspects of the performance of second-generation overcrowded alkene–derived molecular motors.

## INTRODUCTION

The development of artificial molecular machines has unlocked opportunities to exert control over dynamic functions using synthetic nanoscale systems with unprecedented, molecular precision (*1*–*13*). The pioneering synthetic efforts provided guidelines for the design of molecular motors (MMs) and machines (*4*, *11*, *14*–*17*) capable of operation in diluted solutions as well as in supramolecular assemblies, polymers, and the solid state (*8*, *10*, *13*, *18*). Among these structures, overcrowded alkene–derived light-driven MMs emerge as a unique class of small-molecule motors, owing to their intrinsic, chirality-directed 360° unidirectional rotary motion powered by light. The full rotary cycle proceeds through the sequence of alternating photochemical E/Z isomerization and thermal helix inversion (THI) steps, each associated with the inversion of the overall helical chirality of the molecule (*19*, *20*). Following the development of the first-generation motors (*21*), having two stereogenic centers, systematic synthetic modifications led to second-generation motors featuring a single asymmetric carbon center and third-generation mesostructures (*22*, *23*). This structural versatility renders these motors appealing not only for the tuning of dynamic functions (*6*, *13*) but also for functional systems, such as liquid crystals (*24*), responsive catalysis (*25*, *26*), artificial muscle-like fibers (*27*, *28*), metal-organic-frameworks (*29*, *30*), self-assembly architectures (*31*, *32*), as well as functional surfaces (*33*) or responsive membranes (*34*).

**Fig. 1.**
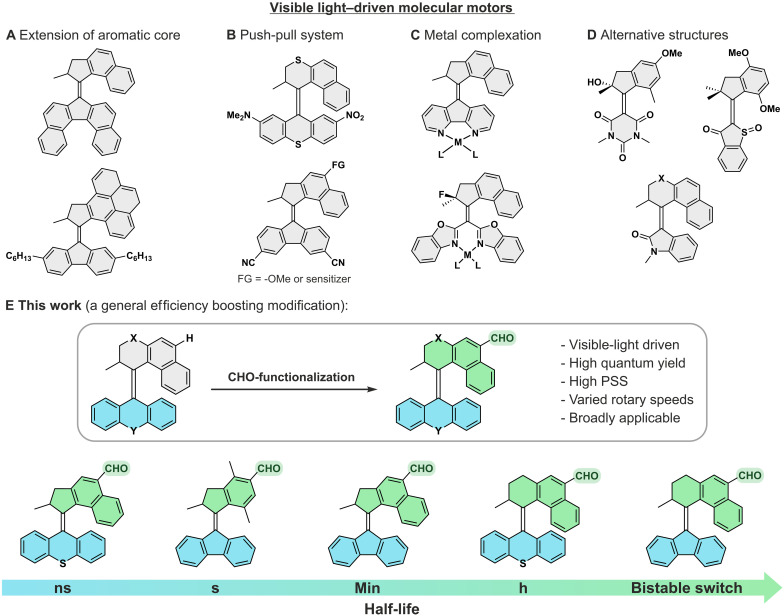
Visible light–driven unidirectional molecular rotary motors and schematic representation of current strategy. Reported visible light–driven MMs (**A** to **D**) and (**E**) our strategy toward visible light–driven motors with high photoefficiency and tunable rotary speeds.

However, to fully explore the applicability of MMs, several challenges remain particularly in enhancing their performance. First, the excitation wavelength of MMs needs to be shifted from the harmful ultraviolet (UV) to the visible light region (*35*). Toward this goal, strategies such as extension of the π system (*36*–*38*), push-pull substitution (*39*, *40*), utilization of photosensitizers (*41*–*44*), and metal-complexation (*45*–*48*) have been developed (Fig. 1, A to C). Alternatively, the redesign of the chromophores to hemithioindigo (*49*–*51*), oxindole (*52*), or barbituric acid (*53*)–based scaffolds were shown to be successful (Fig. 1D). However, all these strategies rely on specific substitution patterns and require elaborate synthetic routes, which limit their applicability in constructing dynamic, visible light–responsive architectures beyond the molecular level. Second, high photoefficiency and high conversion to the metastable isomers at the photostationary state (PSS) must be engineered in the visible region of the absorption spectra. Last, given the diverse range of applications of MMs necessitating varying rotary speeds and any strategy aimed at achieving these two key objectives must be adaptable across multiple scaffolds. Specifically, because of their directional rotation, ultrafast motor cores are widely used to control polymeric gels and to disrupt biological membranes. In contrast, slower motor cores, with half-lives ranging from seconds to hours, often find applications in construction of soft actuators and responsive liquid crystal matrices or porous materials. Consequently, the development of a simple, late-stage, and widely applicable modification strategy of light-driven MMs is needed. This strategy should provide high photoefficiency of the isomerization [high photoisomerization quantum yield (QY)], a red-shift of the excitation wavelength toward visible light, and high PSS distributions. Such a modification strategy would greatly facilitate the applicability of these motors and hence constitutes a fundamental challenge (*6*, *13*, *17*).

Here, we demonstrate a simple, practical, and generally applicable postmodification strategy of second-generation MMs, improving all key aspects of their performance. Recently, we serendipitously found that formylation of first-generation MMs (*54*) led to a drastic improvement in all aspects of their performance. Here, we present the practical extension of this strategy to second-generation motor cores. In this study, we constructed a library of visible light–driven second-generation MM cores with tunable rotary speeds, remarkably high PSS ratios, and substantially higher photoisomerization QYs compared to state-of-the-art visible-light-driven MMs (Fig. 1E).

## RESULTS

### Synthesis of MMs

**Fig. 2.**
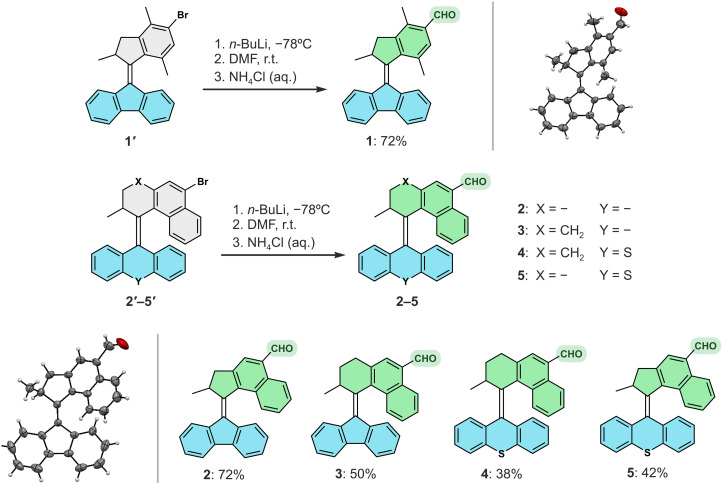
Synthesis of MMs with different rotary speeds. Schematic representation of the formylation strategy and structures of formylated overcrowded alkenes **1** to **5**. SC–x-ray structures of motors **1** (top right corner) and **2** (bottom left corner) are provided.

The parent brominated motors **1′**_**st**_ to **5′**_**st**_ (Fig. 2) were synthesized using a Barton-Kellogg olefination reaction as the key step (*55*). The upper and bottom halves of these precursors were obtained following established procedures developed by our group (*17*). The targeted second-generation motors **1**_**st**_ to **5**_**st**_ (Fig. 2) were synthesized in a single step from their Br-substituted precursors by lithium-halogen exchange and subsequent *N*,*N*′-dimethylformamide quenching to generate formylated motors in good yields (Fig. 2). Single crystals suitable for x-ray diffraction of motor **1**_**st**_ and **2**_**st**_ were successfully obtained by slow evaporation of a concentrated CH_2_Cl_2_ solution (Fig. 2). All previously unreported motors were fully characterized by nuclear magnetic resonance (NMR) spectroscopy and high-resolution mass spectrometry, and these motors show no degradation after 3 years of storage under ambient condition without specific precautions (see the Supplementary Materials).

### Photochemical properties of synthesized MMs

**Fig. 3.**
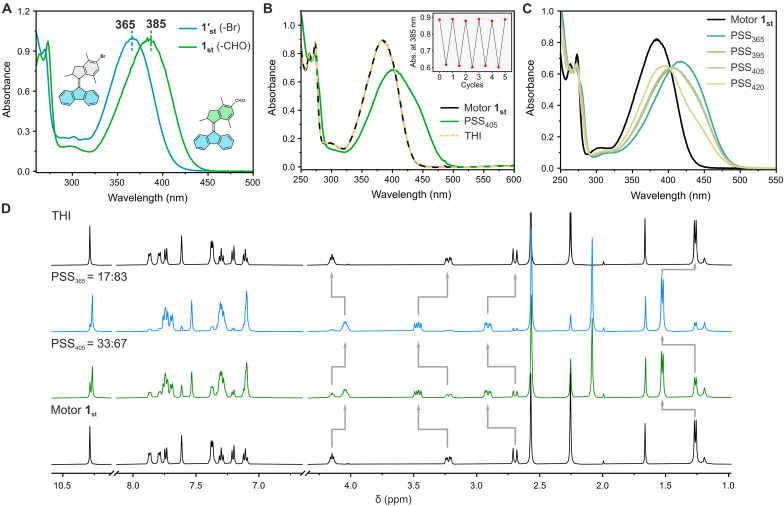
Photochemical and thermal isomerization studies of motor. (**A**) Normalized UV-Vis spectra comparison of **1′**_**st**_ (blue line) and **1**_**st**_ (green line) in CH_2_Cl_2_. (**B**) UV-Vis spectra of motor **1** (CH_2_Cl_2_, −15°C, 28 μM) before irradiation (black solid line), PSS_405_ (green solid line), and THI (yellow dash line). Inset shows the fatigue study of motor **1**_**st**_. (**C**) UV-Vis spectra of motor **1**_**st**_ and PSS at different irradiation wavelengths in CH_2_Cl_2_. (**D**) ^1^H NMR spectra of motor **1**_**st**_ (CD_2_Cl_2_, −50°C, 1.0 mM) before irradiation (bottom black line), PSS_405_ (green line), PSS_365_ (blue line), and THI (top black line).

With these five motors available, we started our investigation with formylated motor **1**_**st**_. Owing to its relatively high rotary speed (*t*_1/2_ = 0.2 min at 20°C) (*56*), the parent *p*-xylene–based second-generation motor has been successfully used in a plethora of applications (*57*) such as bending and helical actuation in liquid-crystal polymer networks (*58*, *59*) or regulating interfacial interactions by surface-assembled MMs (*60*). However, the maximum of the electronic absorption band for this motor scaffolds is centered around 360 nm, which precludes visible-light excitation. The photochemical and thermal isomerization behavior was investigated by UV-Vis absorption and ^1^H NMR spectroscopies at various irradiation wavelengths. The initial UV-Vis absorption maximum of motor **1**_**st**_ (st referring to the stable state) in CH_2_Cl_2_ containing a major band centered at 385 nm, is 20 to 25 nm red-shifted compared to brominated motor **1′**_**st**_ (Fig. 3A) and the nonsubstituted *p*-xylene–based second-generation motor (table S4) (*56*). Upon irradiation with 405-nm light, a bathochromic shift was observed in the UV-Vis absorption spectrum of motor **1**_**st**_, consistent with the formation of **1**_**mst**_ (Fig. 3B, mst referring to the metastable state). A clear isosbestic point at 403 nm was observed (fig. S4) even without any special precautions of the sample such as degassing or base treatment of the solvent, indicating a selective unimolecular photoisomerization process of formylated motor **1**_**st**_. Motor **1** shows a high photostability and no signs of fatigue even upon five full isomerization cycles (Fig. 2B, inset) in aerated, halogenated solvents. In contrast, operation of UV light–driven MMs under these conditions is typically associated with substantial degradation caused by either oxidative or acid-induced damage. In situ ^1^H NMR irradiation below −50°C indicated that the PSS mixture (PSS_405_) for motor **1** consists of predominantly metastable isomer (67:33 of **1**_**mst**_ : **1**_**st**_), which could be further improved to 83:17 (**1**_**mst**_ : **1**_**st**_) by irradiation with 365-nm light (Fig. 3, C and D). Unexpectedly, motor **1**_**st**_ can even be activated by 420-nm light irradiation, which is the maximum absorption wavelength of **1**_**mst**_, indicating the high selectivity in the photoisomerization process of this motor (from **1**_**st**_ to **1**_**mst**_). Higher conversions to the metastable isomer upon irradiation at shorter wavelengths (395 or 365 nm), were further confirmed by the UV-Vis spectra (Fig. 3C and fig. S4). Subsequent heating of the PSS mixture to room temperature (RT) led to a full recovery of the initial UV-Vis absorption and ^1^H NMR spectra of **1**_**st**_, in line with a low thermal barrier of the THI isomerization step for this motor core (Fig. 3B, red dash line; Fig. 3D, top spectrum).

**Fig. 4.**
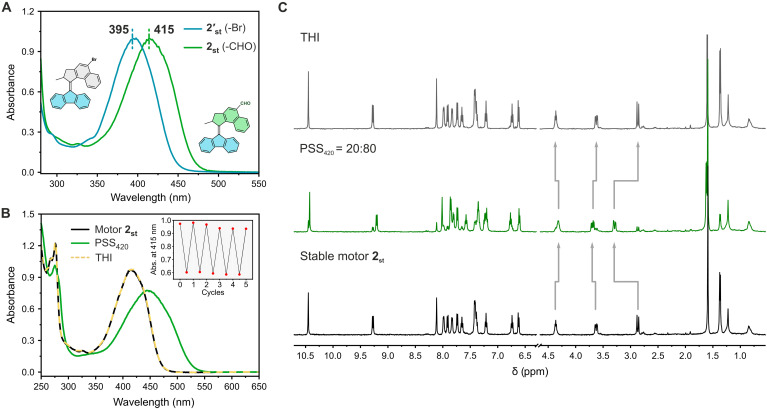
Photochemical and thermal isomerization studies of motor 2. (**A**) Normalized UV-Vis spectra of **2′**_**st**_ (blue line) and **2**_**st**_ (green line) in CH_2_Cl_2_. (**B**) UV-Vis spectra of motor **2**_**st**_ (CH_2_Cl_2_, −15°C, 41 μM) before irradiation (black solid line), at PSS_420_ (green solid line), and after THI (yellow dash line). Inset shows the fatigue study of motor **2**_**st**_. (**C**) ^1^H NMR spectra of motor **2**_**st**_ (CD_2_Cl_2_, −50°C, 2.0 mM) before irradiation (bottom black line), PSS_420_ (green line), and after THI (top black line).

Motor **2** represents an archetype scaffold of a second-generation MM with a central alkene double bond—which is the axle of rotation—flanked by two five-membered rings. The steric congestion, generated by the bulky naphthalene moiety around the motor core drastically impedes the rotary speed (*t*_1/2_ = ~min), which renders this structure suitable for a plethora of applications (*24*, *29*, *30*, *61*, *62*). Therefore, several strategies have been developed to red-shift the excitation wavelength of this scaffold (see also Fig. 1, A and B), including π system extension (*36*), push-pull substitution (*40*), and by using photosensitizers (*41*). However, most of these modifications lead to complicated substitution patterns, which hamper further modification, or display a low photoefficiency of rotation (*17*). Hence, we offer a viable alternative involving a one-step formylation. Motor **2** features a more extended π system compared with motor **1**, resulting in a bathochromically shifted UV-Vis absorption spectrum with the main band centered at 415 nm, that is 20 to 25 nm red-shifted compared to the brominated and unsubstituted parent motor cores (Fig. 4A and table S4). Irradiation of **2** at 420 nm led to a drastic change in the absorption giving rise to a band centered at 450 nm, in line with the formation of **2**_**mst**_ (Fig. 4B). Heating of the sample at 35°C led to the recovery of the initial spectrum, indicating full recovery of **2**_**st**_ (Fig. 4B, red dash line). The photochemical/thermal isomerization cycles could be repeated for five cycles without any noticeable signs of fatigue, further illustrating the high stability of the formylated motor **2** (Fig. 4A, inset). The ratio of isomers established at the PSS generated upon irradiation of **2**_**st**_ at 420 nm was quantified by ^1^H NMR. At the PSS_420_, high conversion to the metastable isomer was observed (80:20 ratio of **2**_**mst**_ : **2**_**st**_) (Fig. 4C), which is higher than other visible light–responsive MMs derived from this scaffold (table S1). These results further illustrate the great advantages of our formylation strategy for designing visible light–driven dynamic systems.

**Fig. 5.**
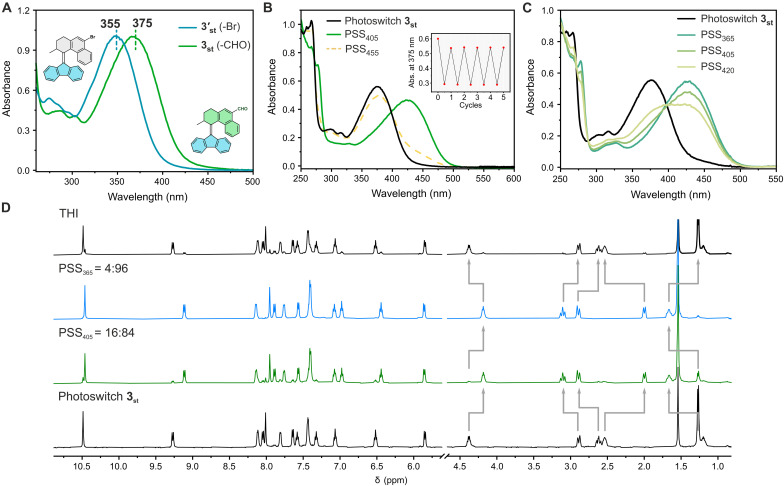
Photochemical and thermal isomerization studies of bistable switch 3. (**A**) Normalized UV-Vis spectra of overcrowded-alkene–based photoswitch **3′**_**st**_ (blue line) and **3**_**st**_ (green line) in CH_2_Cl_2_. (**B**) UV-Vis spectra of overcrowded alkene **3**_**st**_ (CH_2_Cl_2_, RT, 32 μM) before irradiation (black solid line), at PSS_405_ (green solid line), and after THI (yellow dash line). Inset shows the fatigue study of photoswitch **3**_**st**_. (**C**) UV-Vis spectra of photoswitch **3**_**st**_ and PSS mixtures at different irradiation wavelengths in CH_2_Cl_2_. (**D**) ^1^H NMR spectra of photoswitch **3** (CD_2_Cl_2_, −50°C, 1.5 mM) before irradiation (bottom black line), at PSS_405_ (green line), at PSS_365_ (blue line), and at PSS_455_ (top black line).

Because of the high thermal barrier for the THI step, the parent unsubstituted overcrowded alkene **3** is a bistable chiroptical photoswitch as demonstrated in our previous studies (*63*). Therefore, a longer wavelength light could be chosen to trigger backswitching from the metastable isomer to the stable isomer. In addition, the metastable isomer is thermally stable at RT, with a half-life in the order of years. Owing to these specific features, this structure has shown unique advantages as chiropical switch in applications ranging from enantiodivergent catalysis (*25*, *26*) to dynamic smart materials (*64*–*66*). All-visible-light–responsive overcrowded alkene–based chiroptical switches are currently less developed but are highly sought after. Formylation of **3′**_**st**_ to **3**_**st**_ led to a 20-nm red shift of the UV-Vis absorption spectra from 355 to 375 nm (Fig. 5A). UV-Vis spectroscopic studies of **3** showed that the photoisomerization of **3**_**st**_ could be triggered by visible light upon continuous irradiation at 405 nm. A bathochromically shifted band was observed at 425 nm, shifting the maximum absorbance into the green light region, in accordance with the formation of the metastable isomer (Fig. 5B). Unexpectedly, irradiating at a longer wavelength of 420 nm, which is the main absorption band for **3**_**mst**_ isomer, could also induce the isomerization of **3**_**st**_ to **3**_**mst**_, while irradiation at 365 nm led to the highest conversion to the metastable isomer at PSS, as established by UV-Vis spectroscopy (Fig. 5C and fig. S5). The composition of the mixtures at the PSSs established upon irradiation at various wavelengths was further quantified with ^1^H NMR spectroscopy. In line with the UV-Vis absorption data, a relatively high conversion to the metastable isomer was observed upon irradiation of **3**_**st**_ at 405 nm (16:84, **3**_**st**_ : **3**_**mst**_, Fig. 5D) and almost quantitative conversion upon irradiation at 365 nm (4:96, **3**_**st**_ : **3**_**mst**_, Fig. 5D). Accordingly, irradiation at 455 nm led to back-isomerization with high conversion to the thermodynamically stable **3**_**st**_ isomer (**3**_**st**_) at the PSS_455_ (84:16, **3**_**st**_ : **3**_**mst**_, Fig. 5B), thereby illustrating an excellent bidirectional photoswitching with visible light (Fig. 5D). Last, no light-induced fatigue of the photoswitch was observed over several isomerization cycles, further supporting the stability of this visible light–triggered system (Fig. 5B, inset). It is worth mentioning that this is the first example of a chiroptical overcrowded alkene–based photoswitch that can be manipulated by visible light in both directions, expanding the family of P-type switches that respond to visible light (*67*, *68*) such as stiff-stilbenes (*69*, *70*) and ortho-substituted azobenzene derivatives (*71*–*74*).

**Fig. 6.**
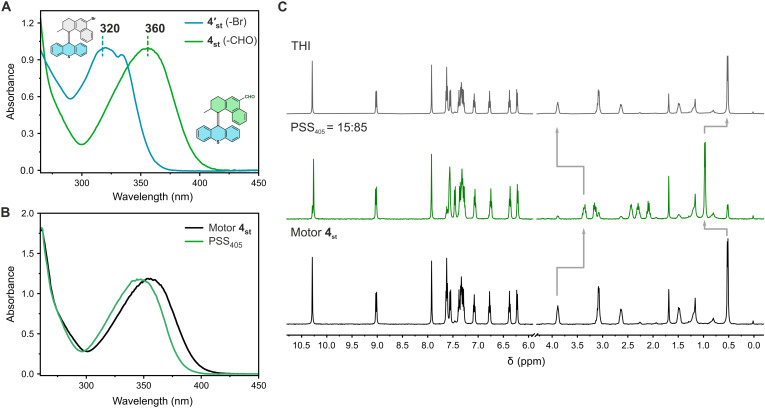
Photochemical and thermal isomerization studies of motor 4. (**A**) Normalized UV-Vis spectra of **4′**_**st**_ (blue line) and **4**_**st**_ (green line) in CH_2_Cl_2_. (**B**) UV-Vis spectra of motor **4**_**st**_ (CH_2_Cl_2_, 0°C, 63 μM) before irradiation (black solid line) and at PSS_405_ (green solid line). (**C**) ^1^H NMR spectra of motor **4**_**st**_ (CD_2_Cl_2_, −50°C, 2.0 mM) before irradiation (bottom black line), at PSS_405_ (green line), and after THI (top black line).

Unlike other second-generation motor cores, motor **4** with the double bond flanked by two six-membered rings exhibits negative photochromism upon photoisomerization between two anti-folded diastereomers (*39*). This scaffold is often used for fabrication of soft-matter–based assemblies and artificial muscle-like fibers (*27*, *28*). As this motif was recently used in the construction of a photoresponsive cell culture scaffold (*28*), shifting its absorption to visible light would be highly advantageous in mitigating the cytotoxic effects of UV light. Consistent with the other scaffolds, formylation of **4′**_**st**_ to **4**_**st**_ led to a 40-nm red shift of the UV-Vis absorption (Fig. 6A). Consequently, upon irradiation of **4**_**st**_ at 405 nm, the stable isomer was converted to the twisted metastable isomer with the absorption band maximum shifting from 360 to 340 nm (Fig. 6B). A remarkably high conversion to the metastable isomer of PSS_405_ of 85:15 was determined by ^1^H NMR upon irradiation of **4**_**st**_ at 405 nm, showing isomerization triggered by visible light (Fig. 6C). In addition, comparable conversions to the metastable **4**_**mst**_ were observed upon irradiation at 365 or 395 nm (table S2 and figs. S6 and S8). Heating the metastable **4**_**mst**_ led to quantitative recovery of the stable **4**_**st**_, as expected after the THI step (Fig. 6C). This method offers a visible light–driven motor system with a rotary axle flanked by two six-membered rings that could potentially be modified and used for biological applications.

**Fig. 7.**
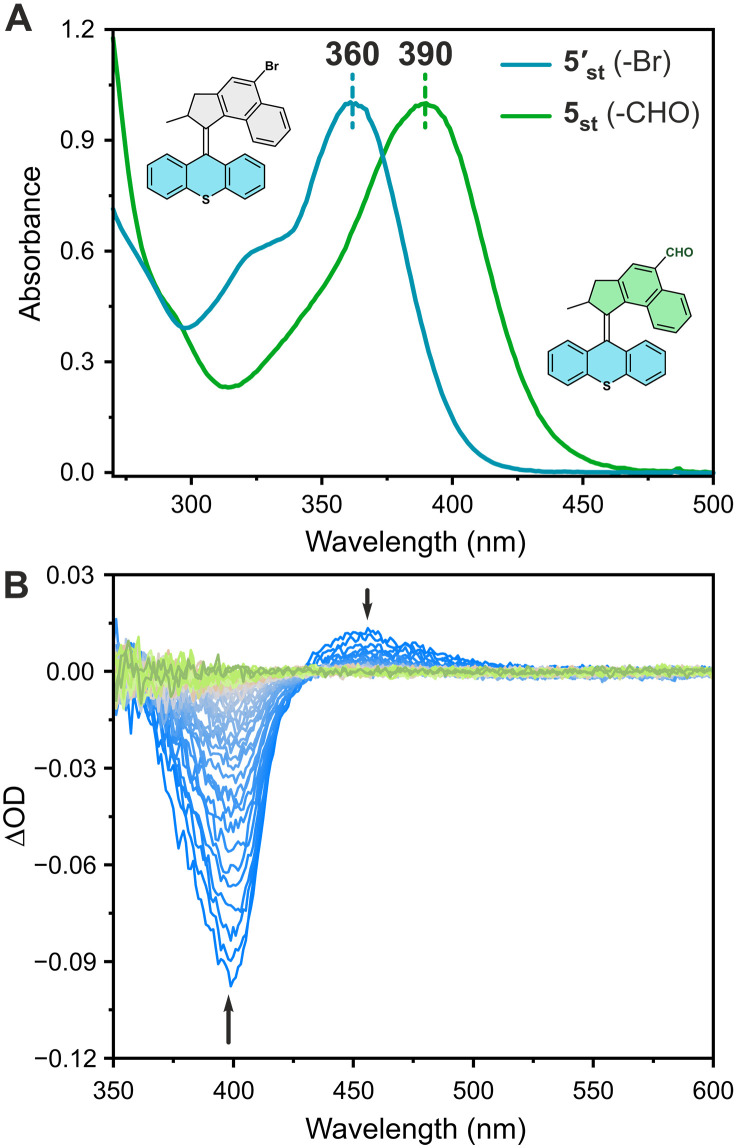
Photochemical and thermal isomerization studies of ultrafast motor 5. (**A**) Normalized UV-Vis spectra of **5′**_**st**_ (blue line) and **5**_**st**_ (orange line) in CH_2_Cl_2_. (**B**) Nanosecond TA spectra of motor **5**_**st**_ (CH_2_Cl_2_, RT) after irradiation with a 390-nm light pulse, upon which the spectra were recorded in steps of increasing delay (blue → green, every 5 ms).

Last, we selected an ultrafast motor core system (the parent structure of motor **5**) with a half-life time of ~270 ns, which has been applied as a privileged motor in a number of systems for its continuous and fast directional rotation under ambient conditions (*75*–*77*). The aldehyde-appended motor **5**_**st**_ displays an absorption maximum at 390 nm, 30 nm red-shifted from the brominated **5′**_**st**_ (Fig. 7A), and its nonsubstituted parent motor core (table S4) (*78*). Because of the ultrafast rotary speed, conventional UV-Vis and ^1^H NMR spectroscopies are not practical to study the photoisomerization behavior of this motor. Because of the extremely low thermal stability of the metastable isomer, we were unable to observe any appreciable concentration of the metastable isomer by ^1^H NMR spectroscopy even when the sample was irradiated at −80°C (fig. S9). On the other hand, isomerization of the motor could conveniently be followed by nanosecond (ns)–pulsed laser transient absorption (TA) spectroscopy. Immediately following the laser pulse at 390 nm, a transient signal with a positive change in optical density was observed, indicating population of a new species. This signal is characterized by a red-shifted absorption band (λ_max_ = 455 nm), coinciding with depletion of the absorption band of the stable isomer (ground state bleach, λ_max_ = 400 nm). We ascribe this signal to the instantaneous formation of the metastable isomer, which over time decays back to zero (Fig. 7B). Furthermore, chiral supercritical fluid chromatography was applied to separate the two enantiomers of motor **5**_**st**_. The two enantiomers were identified by circular dichroism (CD) spectroscopy (fig. S22). These enantiopure motors can be used for applications necessitating chirality-correlated rotations (clockwise and counter-clockwise motion) (*13*).

### THI parameters of investigated MMs

The Gibbs free energies of activation for the THI steps for MMs **1**_**mst**_, **2**_**mst**_, and **4**_**mst**_ were determined by Eyring analysis (figs. S1 to S3 and Table 1). The experimental trend in the values of the rate of THI closely follows that of the parent scaffolds, which further illustrates that the rate of THI is predominantly determined by steric congestion in the fjord region between the top and bottom parts of the molecule. However, we found that the half-lives of the aldehyde-appended motors are consistently slightly longer that those of the parent motors. The synthesized motors cover a wide range of rotary rates, starting from lifetimes in the range of microseconds up to several hours. Specifically, motors **1** and **2** have distinct rotary speeds, with a half-life of 4.6 min at 20°C determined for motor **2**_**mst**_, 14 times higher than for motor **1**_**mst**_ (*t*_1/2_ = 20 s at 20°C). In contrast, for motor **4**_**mst**_, a half-life of 1.9 hours was determined, the longest in the series reported here. We note that the metastable overcrowded alkene **3**_**mst**_ is expected to be thermally stable; however, the thermal isomerization of these structures is known to follow two distinct mechanisms (*63*, *79*). TA spectroscopy provided a half-life time of 69.0 μs for the motor **5**_**mst**_ (fig. S23), also slower in comparison that of the parent motor (*78*). In summary, by simple formylation of different motor cores, we obtained visible-light-driven second-generation MMs with varied rotary speeds ranging from nanoseconds to hours, as well as a highly stereoselective chiroptical bistable photoswitch.

**Table 1.** THI steps of motors 1, 2, 4, and 5.

Motor	∆^‡^G* (kJ/mol)	*t*_1/2_ (20°C)^†^
**1**_**m**_	80.0 ± 0.3	20.1 s
**2**_**m**_	86.4 ± 0.1	4.6 min
**4**_**m**_	94.2 ± 0.3	1.9 hours
**5**_**m**_	-	69.0 μs^§^

*Determined at 20°C.

†Defined as ln (2)/*k* at 20°C.

§Half-life of motor **5**_**m**_ was determined by transient spectroscopy.

### Computational studies of investigated MMs

Density functional theory calculations studies were performed to gain a better understanding of the structural parameters and the relative energies of the investigated MMs. First, the calculated trend in λ_max_ of MMs is in excellent agreement with the experimentally observed results (table S4), clearly indicating that the formylation of MMs induces a redshift of the absorption of MMs compared to the Br- or H-substituted MMs. The calculated relative Gibbs free energies of stable and metastable diastereomers of CHO-appended MMs as well as the transition states for THI (tables S6 to S10), were used to approximate the corresponding Gibbs free energies of activation of aldehyde-appended MMs (table S5). The calculated Gibbs free energies of activations followed the same trend as the experimental data, that is, the trend of steric crowding around the double bond. Specifically, motor **5** has the lowest activation energy for the THI step, and for overcrowded alkene **3,** a thermal E-Z isomerization pathway is preferred over a THI step (*63*), which is in agreement with the available experimental data. The computational data thus further confirm the observed rotary behavior of the investigated MMs.

### QY determinations and TA measurements of investigated MMs

QYs of the photoisomerization of MMs (**1**, **2**, and **4**) and overcrowded alkene–derived bistable photoswitch **3** were determined by comparing the rate of their photoisomerization to that of a ferrioxalate standard under the same photon flux (*40*). All motors exhibit substantially higher QYs than current second-generation motor systems at both 390 and 365 nm for the forward photoreactions. For nearly all motors and at both wavelengths (390 and 365 nm), a favorable ratio of QYs for forward and back photoreactions was found, indicating that an excess of the metastable isomer at the PSS stems not only from band separation between the two isomers but also from a higher tendency of the excited molecule to progress along the reaction coordinate. The exception to this pattern is motor **4**, for which a very high QY (88.4%) at 365 nm of undesired **4**_**ms**_ → **4**_**st**_ isomerization was observed. However, adjusting the irradiation wavelength to 390 nm restores the favorable ratio of the QY of isomerization (Table 2). These observations are consistent with high conversions to the metastable diastereomers at PSS of the respective motors. Overall, large differences between QYs at 365 and 390 nm were observed for all the investigated motors, indicating anti-Kasha’s rule behavior of all investigated MMs and the switch. Similar deviations from Kasha rule were also observed in overcrowded alkenes (*63*, *80*) and more recently, for hemithioindigo-derived (*81*) MMs, initiating a complex topography of the excited state potential energy surface along the reaction coordinate (*82*). Motor **2** shows among all investigated motors a record high photoisomerization QY (26 to 28%) at either 420 or 390 nm as well as the highest QY among the second-generation overcrowded alkene–derived MMs reported to date (*17*) (Table 2 and table S1). Furthermore, compared to other substituents attached at this position of the naphthalene-derived motors (*80*), the formyl substituent provides the highest nominal value of QY while also reducing the QY of the backward photoreaction (table S11). As for switch **3**, the QY at 390 nm was measured to be ~ 20%, again the highest among this overcrowded alkene–type of bistable photoswitches (*63*). Last, femtosecond (fs)–TA spectroscopy studies on motor **1** and **2** indicated no involvement of the triplet excited state intermediates in the isomerization, which is consistent with our previous observations on formylated motor second-generation MMs (*54*) and indicating that the aldehyde substituent does not fundamentally alter the singlet-dominated isomerization mechanism of the MMs (figs. S24 to S26). While alternative carbonyl substituents at the same position, such as ketone, should also endow these positive effects, the current synthetic approach encompassing attaching aldehyde is simple and general. However, a simple electronic effect has been ruled out since no similar effects have been found in previously synthesized second generation molecular motors (*80*). The computational study indicated that the red shift of the absorption spectra of all the motors could be ascribed to the stabilization of the lowest unoccupied molecular orbital by the aldehyde group (figs. S33 to S34). However, further studies aimed at unravelling the impact of aldehyde on QY found only marginal differences in the excited state between the aldehyde-appended and parent motors (figs. S35 to S38). Hence, the boosting effect of aldehyde likely originates from combinations of several subtle factors. Future efforts focusing on spectroscopic and theoretical studies may help to identify transient species and underpin processes responsible for the boosted performance of these light-driven molecular motors.

**Table 2.** QYs (%) of photo-isomerization steps of motor 1 to 4.

Motor	Φ_st→mst_ (390 nm)	Φ_mst→st_ (390 nm)	Φ_st→mst_ (365 nm)	Φ_mst→st_ (365 nm)
**1**	21.4	19.5	18.1	5.1
**2**	26.5	8.5	27.7*	17.4*
**3**	19.2	1.2	12.5^†^	1.8^‡^
**4**	9.0	7.6	13.4	88.4

*QY obtained upon irradiation at 420 nm.

†QY obtained upon irradiation at 445 nm from **3**_**mst**_ to **3**_**st**_.

‡QY obtained upon irradiation at 445 nm light from **3**_**st**_ to **3**_**mst**_.

## DISCUSSION

In conclusion, we developed a simple, easily accessible, effective, and general post-synthetic modification strategy of second-generation MMs. The one-step formylation was found to be widely applicable, thus allowing us to access a variety of MMs featuring half-lives ranging from nanoseconds to years. The optimized synthetic routes for these compounds allow for the straightforward synthesis of large quantities of the material. The five substrates that were examined in this study all displayed excellent photochemical switching behavior, with absorption maxima consistently shifted toward the visible-light region compared to the parent structures and excellent resistance to light-induced fatigue. QYs of the photochemical isomerization were found to be considerably higher compared to other reported visible light–driven MMs. Hence, this study provides a simple access to a wide range of visible light–driven MM scaffolds, most of which were previously only operated using UV light. We envision that these robust MMs will provide previously undiscovered approaches toward smart materials and biological applications, thus bringing these molecules one step closer to practical applications.

## MATERIALS AND METHODS

The UV-Vis, CD, and NMR irradiation experiments were performed using fiber-coupled LEDs (M365F1, M395F2, M405FP1, M420F2, and M455F1) obtained from Thorlabs Inc. UV-Vis absorption spectra were measured on a Hewlett-Packard 8453 diode array spectrometer in a 1-cm quartz cuvette. CD spectra were recorded on a Jasco J-815 CD spectrometer. All UV experiments are performed directly using the solvents from the solvent purification system without degassing. The NMR irradiation study was performed using CD_2_Cl_2_ treated with K_2_CO_3_ without degassing.

## Supplementary Materials

### The PDF file includes:

Supplementary Text Figs. S1 to S40 Tables S1 to S12 Legend for data S1 References
