# Supplementary material for: General strategy for boosting the performance of speed-tunable rotary molecular motors with visible light
Source: Sci Adv. 2025 Feb 19;11(8):eadr9326. doi: 10.1126/sciadv.adr9326 (PMC11838004; doi:10.1126/sciadv.adr9326)
Supplement: Supplementary file 1 — Supplementary Text Figs. S1 to S40 Tables S1 to S12 Legend for data S1 References [file sciadv.adr9326_sm.pdf]

Science Advances
AAAS

# Supplementary Materials for

## General strategy for boosting the performance of speed-tunable rotary molecular motors with visible light

Jinyu Sheng *et al.*

Corresponding author: Wojciech Danowski, w.danowski2@uw.edu.pl; Ben L. Feringa, b.l.feringa@rug.nl

*Sci. Adv.* **11**, eadr9326 (2025)
DOI: 10.1126/sciadv.adr9326

**The PDF file includes:**

Supplementary Text
Figs. S1 to S40
Tables S1 to S12
Legend for data S1
References

**Other Supplementary Material for this manuscript includes the following:**

Data S1

Supplementary Text

## General Information

All reagents from commercial sources such as Aldrich, TCI, Fluorochem, combi-Blocks were used as received. Anhydrous DCM, THF, Diethyl ether was obtained from a solvent purification system (MBRAUN SPS systems, MBSPS-800). Flash column chromatography was performed using silica gel ($SiO_2$) purchased from Merck (type 9385, 230-400 mesh) or on a Büchi Reveleris purification system with Büchi cartridges. NMR spectra were recorded on Varian AMX400 ($^1H$: 400 MHz, $^{13}C$: 100 MHz) and Varian Unity Plus ($^1H$: 500 MHz, $^{13}C$: 125 MHz) spectrometers. Chemical shifts are quoted in parts per million (ppm) relative to the residual solvent signal (for $CDCl_3$ δ 7.26 for 1H, δ 77.16 for $^{13}C$ and for $CD_2Cl_2$ δ 5.32 for $^1H$, δ 53.84 for $^{13}C$). For $^1H$ NMR spectroscopy, the splitting pattern of peaks is designated as follows: s (singlet), d (doublet), t (triplet), m (multiplet), br (broad), or dd (doublet of doublets). High resolution mass spectrometry (ESI or APCI-MS) was performed on a LTQ Orbitrap XL spectrometer with ESI ionization.

Experimental procedures

**9-(5-bromo-2,4,7-trimethyl-2,3-dihydro-1H-inden-1-ylidene)-9H-fluorene (1')**.

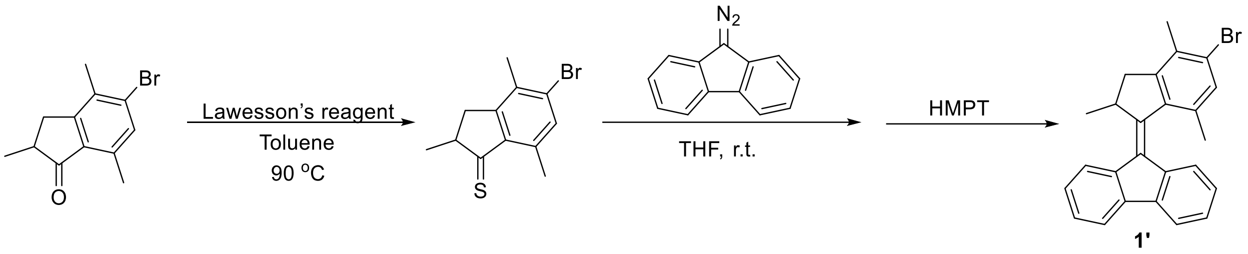


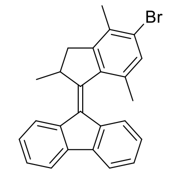


Lawesson's reagent (2.7 g, 6.4 mmol) and indanone[83] (800.0 mg, 3.2 mmol) were dissolved in dry toluene (30 mL) in a vial under $N_2$ atmosphere. The mixture was heated at 90 °C for 4 h under stirring. The reaction progress was followed by TLC (pentane:$CH_2Cl_2$ = 3:1). Subsequently, the mixture was poured onto the column to purify the thioketone by quick column chromatography ($SiO_2$, pentane:$CH_2Cl_2$ = 3:1). The green fraction was collected and concentrated under reduced pressure to yield the crude thioketone as a solid (680.0 mg, 2.5 mmol, 78%). In another vial with 9-diazo-9H-fluorene[44] (406.0 mg, 2.1 mmol) dissolved in THF under $N_2$ atmosphere, the prepared thioketone dissolved in THF (10 mL) was added to the solution and the resulting mixture was allowed to stir overnight. Next, HMPT (1.5 mL) was added, and the reaction mixture was allowed to stir for another 24 h. Next, the reaction mixture was poured into water, extracted with EtOAc. The organic layer was collected, washed with water, brine, dried over $Na_2SO_4$, filtrated and concentrated in vacuo. The crude product was purified by column chromatography ($SiO_2$, pentane) to afford motor **1'** as a yellow solid (640.0 mg, 1.6 mmol, 76%).

**$^1$H NMR** (400 MHz, $CDCl_3$) δ 7.90 – 7.83 (m, 1H), 7.83 – 7.77 (m, 1H), 7.74 (d, $J$ = 7.6 Hz, 1H), 7.42 – 7.27 (m, 5H), 7.13 (t, $J$ = 7.6 Hz, 1H), 4.14 (p, $J$ = 6.6 Hz, 1H), 3.27 (dd, $J$ = 14.6, 6.3 Hz, 1H), 2.63 (d, $J$ = 14.8 Hz, 1H), 2.37 (s, 3H), 2.22 (s, 3H), 1.32 (d, $J$ = 6.8 Hz, 3H).

**$^{13}$C NMR** (101 MHz, $CDCl_3$) δ 151.0, 147.0, 140.11, 139.7, 139.7, 139.4, 137.8, 136.2, 132.7, 131.8, 130.8, 127.5, 127.2, 127.1, 126.8, 126.1, 124.1, 123.8, 119.9, 119.4, 44.3, 41.1, 20.9, 19.1, 18.8.

HRMS spectrum was not obtained due to the ionization issue of the compound by either ESI or APCI method.

**1-(9H-fluoren-9-ylidene)-2,4,7-trimethyl-2,3-dihydro-1H-indene-5-carbaldehyde (1)**.

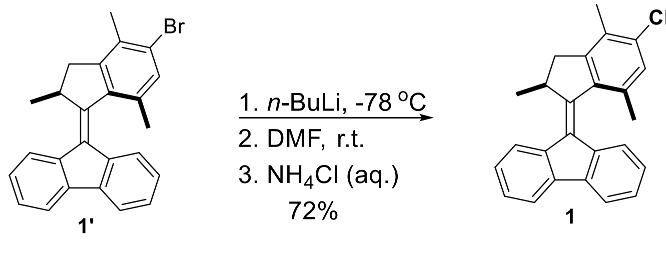

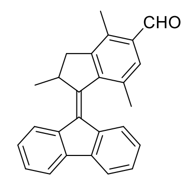

Under a $N_2$ atmosphere, 9-(5-bromo-2,4,7-trimethyl-2,3-dihydro-1H-inden-1-ylidene)-9H-fluorene (256.0 mg, 0.64 mmol) was dissolved in THF (20 mL) and cooled to -78 °C. N-butyllithium (1.6 M in hexane, 0.52 mL, 0.83 mmol) was slowly added to the solution and the mixture was allowed to stir for 15 min. Anhydrous DMF (1.0 mL) was then added, and the mixture was allowed to stir for 1 h at room temperature. The reaction was quenched by saturated aqueous $NH_4Cl$, and extracted with EtOAc. The organic layer was collected and washed with water, brine, dried over $Na_2SO_4$, filtrated and concentrated in vacuo. The crude product was purified by column chromatography ($SiO_2$, pentane:EtOAc = 5:1) to afford motor **1** as a yellow solid (160.0 mg, 0.46 mmol, 72%).

**$^1$H NMR** (400 MHz, $CDCl_3$) δ 10.33 (s, 1H), 7.92 – 7.84 (m, 1H), 7.83 – 7.76 (m, 1H), 7.73 (d, *J* = 7.6 Hz, 1H), 7.62 (s, 1H), 7.43 – 7.27 (m, 4H), 7.11 (t, *J* = 7.6 Hz, 1H), 4.19 (p, *J* = 6.7 Hz, 1H), 3.26 (dd, *J* = 15.1, 6.0 Hz, 1H), 2.70 (d, *J* = 15.0 Hz, 1H), 2.62 (s, 3H), 2.32 (s, 3H), 1.32 (d, *J* = 6.8 Hz, 4H).

**$^{13}$C NMR** (101 MHz, $CDCl_3$) δ 192.9, 150.1, 147.2, 145.7, 140.5, 140.1, 139.5, 137.7, 134.8, 134.4, 133.9, 133.5, 133.0, 128.1, 127.8, 127.3, 127.0, 124.4, 124.0, 120.0, 119.5, 44.0, 39.9, 21.1, 19.1, 14.9.

**HRMS** (APCI pos) calcd $C_{26}H_{23}O$ $[M+H]^+$ : 351.1743, found 351.1752.

**1-(9H-fluoren-9-ylidene)-2-methyl-2,3-dihydro-1H-cyclopenta[a]naphthalene-5-carbaldehyde (2).**

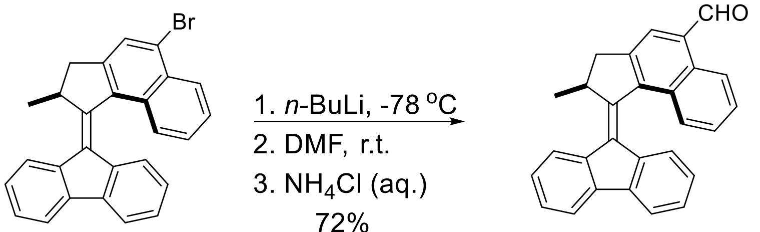


2'    2

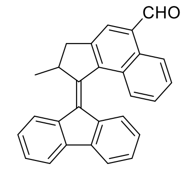

Under a $N_2$ atmosphere, 9-(5-bromo-2-methyl-2,3-dihydro-1H-cyclopenta[a]naphthalen-1-ylidene)-9H-fluorene[80] (800.00 mg, 1.89 mmol) was dissolved in THF (20 mL) and cooled to -78 °C. Then, n-butyllithium (1.6 M in hexane, 1.42 mL, 2.27 mmol) was slowly added to the solution and the mixture was allowed to stir for 15 min. Then anhydrous DMF (2.5 mL) was added, and the mixture was allowed to stir for 1 h at room temperature. The reaction was quenched by saturated aqueous $NH_4Cl$, and the mixture was extracted with EtOAc, washed with water, brine, dried over $Na_2SO_4$, filtrated and concentrated in vacuo. The crude product was purified by column chromatography ($SiO_2$, pentane:EtOAc = 1:1) to afford motor **2** as a yellow solid (530.0 mg, 1.37 mmol, 72%).

**$^1$H NMR** (500 MHz, $CD_2Cl_2$) δ 10.50 (s, 1H), 9.27 (d, *J* = 8.6 Hz, 1H), 8.11 (s, 1H), 8.02 – 7.98 (m, 1H), 7.93 (d, *J* = 8.5 Hz, 1H), 7.86 – 7.81 (m, 1H), 7.74 (d, *J* = 7.5 Hz, 1H), 7.66 (t, *J* = 7.7 Hz, 1H), 7.44 – 7.37 (m, 3H), 7.22 (t, *J* = 7.5 Hz, 1H), 6.75 (t, *J* = 7.6 Hz, 1H), 6.63 (d, *J* = 7.9 Hz, 1H), 4.42 – 4.35 (m, 1H), 3.65 (dd, *J* = 15.0, 5.7 Hz, 1H), 2.88 (d, *J* = 15.1 Hz, 1H), 1.40 (d, *J* = 6.8 Hz, 3H).

**$^{13}$C NMR** (101 MHz, $CDCl_3$) δ 193.07, 149.06, 145.31, 143.60, 140.73, 140.21, 139.58, 136.87, 133.94, 133.37, 132.54, 130.32, 130.25, 128.44, 128.06, 128.02, 127.94, 127.44, 127.38, 126.33, 126.10, 125.45, 124.64, 120.00, 119.32, 45.56, 41.63, 19.24.

**HRMS** (APCI pos) calcd $C_{28}H_{21}O$ $[M+H]^+$ : 373.1587, found 373.1588.

**9-bromo-4-(9H-fluoren-9-ylidene)-3-methyl-1,2,3,4-tetrahydrophenanthrene (3').**

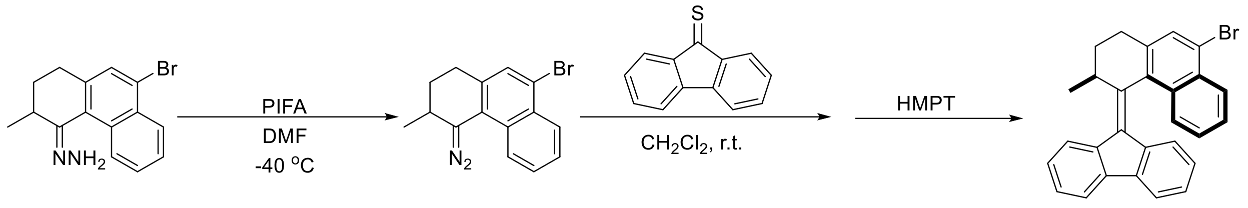


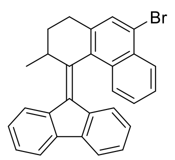


Lawesson's reagent (570.0 mg, 1.4 mmol) and 9H-fluoren-9-one[4] (360.0 mg, 2 mmol) were dissolved in dry toluene (15 mL) under $N_2$ atmosphere. The reaction mixture was heated at 90 °C and allowed to stir for 2 h upon which the mixture turned orange. The progress of the reaction was followed by TLC (pentane:$CH_2Cl_2$ = 3:1). Then the reaction mixture was poured directly on previously prepared silica column and purified by a quick column chromatography ($SiO_2$, pentane:$CH_2Cl_2$ = 3:1). The slightly orange fraction was collected and concentrated under reduced pressure to yield the crude thioketone as a brown solid (to prevent hydrolysis, the product was kept wet with $CH_2Cl_2$ and stored under $N_2$ atmosphere). In another two-neck flask under $N_2$ flow, hydrazone[63] (120.8 mg, 0.4 mmol) was dissolved in DMF (4 mL). This solution was cooled to -40 °C and bis(trifluoroacethoxy)iodobenzene (180.6 mg, 0.42 mmol) in DMF (3 mL) was added. The mixture was allowed to stir for 3 min until the color turned to pink, indicative of the *in situ* formation of the diazo compound. Then, a solution of 9H-fluoren-9-thione (78.4 mg, 0.4 mmol) in dry $CH_2Cl_2$ (5 mL) was added to the mixture. The reaction mixture was allowed to warm to room temprature and stir for 4 h. Next, HMPT (Tris(dimethylamino)phosphine) (0.4 mL) was added and the mixture was left for another 24 h. Subsequently, the mixture was poured into water and extracted with EtOAc. The organic layer was collected, washed with water, brine, dried over $Na_2SO_4$, filtrated and concentrated in vacuo. The crude product was purified by column chromatography ($SiO_2$, pentane:EtOAc = 50:1) to afford switch **3'** as a orange solid (165.7 mg, 0.38 mmol, 95%).

**$^1$H NMR** (400 MHz, $CDCl_3$) δ 8.29 (d, $J$ = 8.5 Hz, 1H), 8.13 – 8.06 (m, 1H), 7.95 (d, $J$ = 8.5 Hz, 1H), 7.81 (d, $J$ = 6.6 Hz, 2H), 7.64 (d, $J$ = 7.6 Hz, 1H), 7.52 – 7.38 (m, 3H), 7.26 (d, $J$ = 15.4 Hz, 2H), 7.08 (t, $J$ = 7.4 Hz, 1H), 6.58 (t, $J$ = 7.7 Hz, 1H), 5.96 (d, $J$ = 8.0 Hz, 1H), 4.33 (h, $J$ = 7.0 Hz, 1H), 2.73 (dt, $J$ = 14.3, 3.6 Hz, 1H), 2.57 (td, $J$ = 13.4, 5.2 Hz, 1H), 2.50 – 2.41 (m, 1H), 1.26 (d, $J$ = 6.9 Hz, 3H), 1.18 (td, $J$ = 12.5, 6.1 Hz, 1H).

**$^{13}$C NMR** (101 MHz, $CDCl_3$) δ 143.5, 141.1, 140.5, 139.7, 138.0, 137.8, 134.18, 133.9, 133.4, 130.8, 130.0, 127.8, 127.7, 127.5, 127.2, 127.2, 126.7, 126.6, 125.7, 125.4, 124.8, 123.5, 119.9, 119.1, 34.7, 31.0, 29.5, 21.0.

**HRMS** (APCI pos) calcd $C_{28}H_{22}BrS$ $[M+H]^+$ : 469.0620, found 469.0621.

**4-(9H-fluoren-9-ylidene)-3-methyl-1,2,3,4-tetrahydrophenanthrene-9-carbaldehyde (3).**

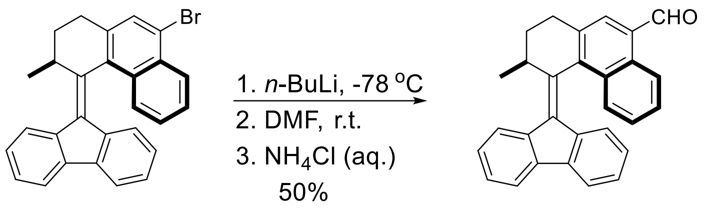


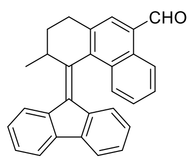


Under a $N_2$ atmosphere, 9-bromo-4-(9H-fluoren-9-ylidene)-3-methyl-1,2,3,4-tetrahydrophenanthrene (87.2 mg, 0.2 mmol) was dissolved in THF (5 mL) and the mixture was cooled to -78 °C. N-butyllithium (1.6 M in hexane, 0.25 mL, 0.4 mmol) was slowly added to the solution which was allowed to stir for 15 min. Next, anhydrous DMF (0.3 mL) was added, and the mixture was allowed to stir for 1 h at room temperature. The reaction was quenched by saturated aqueous $NH_4Cl$, and the mixture was extracted with EtOAc, washed with water, brine, dried over $Na_2SO_4$, filtrated and concentrated in vacuo. The crude product was purified by column chromatography ($SiO_2$, pentane:EtOAc = 5:1) to afford motor **3** as a yellow solid (38.6 mg, 0.1 mmol, 50%).

$^1$H NMR (400 MHz, $CDCl_3$) δ 10.52 (s, 1H), 9.28 (d, *J* = 8.6 Hz, 1H), 8.07 (dd, *J* = 15.4, 7.8 Hz, 2H), 7.98 (s, 1H), 7.84 – 7.76 (m, 1H), 7.65 (s, 2H), 7.43 (ddd, *J* = 6.5, 3.8, 1.7 Hz, 2H), 7.36 – 7.28 (m, 1H), 7.07 (t, *J* = 7.4 Hz, 1H), 6.54 (t, *J* = 7.7 Hz, 1H), 5.86 (d, *J* = 8.0 Hz, 1H), 4.38 (p, *J* = 7.2 Hz, 1H), 2.87 (dt, *J* = 14.2, 3.6 Hz, 1H), 2.64 (td, *J* = 13.5, 5.1 Hz, 1H), 2.58 – 2.45 (m, 1H), 1.28 (d, *J* = 6.9 Hz, 3H), 1.20 (dt, *J* = 12.4, 5.7 Hz, 1H).

**$^{13}$C NMR** (101 MHz, $CDCl_3$) δ 193.5, 142.9, 141.5, 141.3, 140.0, 139.0, 137.8, 137.5, 135.8, 135.3, 132.8, 131.0, 129.7, 128.2, 127.8, 127.5, 127.4, 126.8, 125.8, 125.6, 125.0, 124.9, 120.0, 119.2, 34.8, 31.3, 29.6, 21.0.

**HRMS** (APCI pos) calcd $C_{29}H_{23}O$ $[M+H]^+$ : 387.1743, found 387.1753.

**9-(9-bromo-3-methyl-2,3-dihydrophenanthren-4(1H)-ylidene)-9H-thioxanthene (4')**.

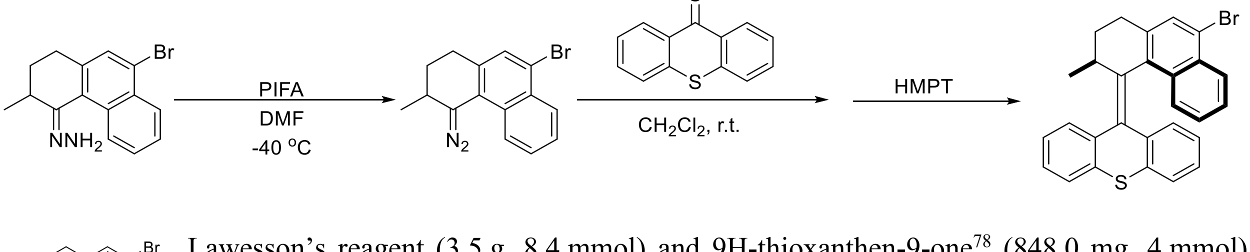


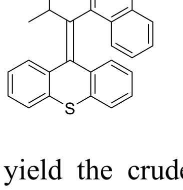


Lawesson's reagent (3.5 g, 8.4 mmol) and 9H-thioxanthen-9-one[78] (848.0 mg, 4 mmol) were dissolved in dry toluene (15 mL) in a vial under $N_2$ atmosphere. The mixture was heated at 90 °C and allowed to stir for 1 h. Then the mixture was poured directly onto the silica column and purified by a quick column chromatography ($SiO_2$, pentane:$CH_2Cl_2$ = 3:1). The thioketone fraction was collected and concentrated under reduced pressure to yield the crude thioketone as a dark brown solid. In another two-neck flask under $N_2$ atmosphere,

hydrazone[63] (151.0 mg, 0.5 mmol) was dissolved in DMF (10 mL). The mixture was cooled to -40 °C, and bis(trifluoroacethoxy)iodobenzene (236.0 mg, 0.55 mmol) in DMF (3 mL) was added to the stirred solution. The mixture was allowed to stir for 3 mins upon which the color turned to pink, indicative of the *in situ* formation of the diazo compound. A solution of the 9H-thioxanthene-9-thione (114.0 mg, 0.5 mmol) in dry DMF (5 mL) was added to the mixture. The mixture was allowed to warm to room temprature and stir for 24 h. Then, the mixture was poured into water and extracted with EtOAc. Then the crude was concentrated and purified by column chromatography ($SiO_2$, pentane) to afford white episulfide. The episulfide was dissolved in toluene, and HMPT (Tris(dimethylamino)phosphine) (0.4 mL) was added and the mixture was stirred at 65 °C for another 16 h. The mixture was dilluted with EtOAc, washed with water, brine, dried over $Na_2SO_4$, filtrated and concentrated in vacuo. The crude product was purified by column chromatography ($SiO_2$, pentane) to afford motor **4'** as a white solid (62.4 mg, 0.13 mmol, 25% two steps).

**$^1$H NMR** (400 MHz, $CD_2Cl_2$) δ 8.01 (d, $J$ = 8.5 Hz, 1H), 7.74 (s, 1H), 7.64 (td, $J$ = 7.5, 1.3 Hz, 2H), 7.54 (d, $J$ = 8.5 Hz, 1H), 7.41 – 7.33 (m, 2H), 7.32 – 7.20 (m, 2H), 7.03 (t, $J$ = 7.7 Hz, 1H), 6.77 (td, $J$ = 7.6, 1.4 Hz, 1H), 6.42 (td, $J$ = 7.5, 1.2 Hz, 1H), 6.32 (d, $J$ = 7.7 Hz, 1H), 3.90 (td, $J$ = 10.2, 5.2 Hz, 1H), 3.07 (ddd, $J$ = 15.1, 10.5, 7.7 Hz, 1H), 2.95 (ddd, $J$ = 15.1, 6.5, 2.9 Hz, 1H), 2.67 – 2.55 (m, 1H), 1.48 (dd, $J$ = 19.8, 3.5 Hz, 1H), 0.61 (d, $J$ = 6.8 Hz, 3H).

**$^{13}$C NMR** (101 MHz, $CDCl_3$) δ 139.4, 138.7, 138.2, 136.6, 136.1, 134.6, 134.2, 132.4, 131.4, 130.5, 129.8, 128.5, 128.4, 128.0, 126.9, 126.7 (2*CH), 126.3, 126.1, 126.1, 125.8, 125.7, 125.6, 122.0, 31.0, 30.8, 28.8, 21.8.

**HRMS** (APCI pos) calcd $C_{28}H_{22}BrS$ $[M+H]^+$ : 469.0620, found 469.0621.

**3-methyl-4-(9H-thioxanthen-9-ylidene)-1,2,3,4-tetrahydrophenanthrene-9-carbaldehyde (4)**.

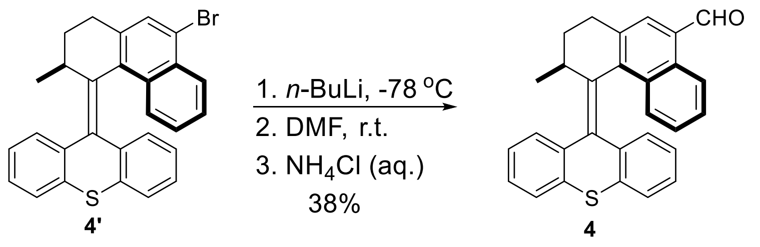


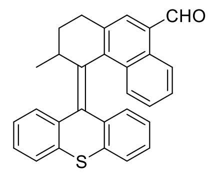


Under a $N_2$ atmosphere, 9-(9-bromo-3-methyl-2,3-dihydrophenanthren-4(1H)-ylidene)-9H-thioxanthene (64.2 mg, 0.13 mmol) was dissolved in THF (4 mL) and cooled to -78 °C. Then, n-butyllithium (1.6 M in hexane, 0.125 mL, 0.2 mmol) was slowly added to the solution and the mixture was allowed to stir for 15 mins. Next, anhydrous DMF (0.2 mL) was added and the mixture and allowed to stir for 1 h at room temperature. The reaction was quenched by saturated aqueous $NH_4Cl$, and the mixture was extracted with EtOAc, washed with water, brine, dried over $Na_2SO_4$, filtrated and concentrated in vacuo. The crude product was purified by column chromatography ($SiO_2$, pentane:EtOAc = 5:1) to afford motor **4** as a white solid (20.9 mg, 0.1 mmol, 38%).

**$^1$H NMR** (400 MHz, $CDCl_3$) δ 10.43 (s, 1H), 9.01 (d, $J$ = 8.6 Hz, 1H), 7.89 (s, 1H), 7.63 (dt, $J$ = 7.6, 3.6 Hz, 3H), 7.34 (ddd, $J$ = 18.0, 13.8, 7.5 Hz, 4H), 7.08 (t, $J$ = 7.7 Hz, 1H), 6.76 (t, $J$ = 7.6 Hz, 1H), 6.37 (t, $J$

= 7.5 Hz, 1H), 6.20 (d, $J$ = 7.8 Hz, 1H), 3.97 (td, $J$ = 7.7, 3.7 Hz, 1H), 3.19 – 3.06 (m, 2H), 2.66 (dtd, $J$ = 11.7, 8.0, 3.0 Hz, 1H), 1.34 – 1.26 (m, 1H), 0.66 (d, $J$ = 6.8 Hz, 3H).

**$^{13}$C NMR** (101 MHz, $CDCl_3$) δ 193.4, 142.3, 138.3, 138.2, 138.0, 136.2, 136.0, 135.6, 134.6, 133.4, 130.6, 130.2, 129.6, 128.4, 128.3, 128.0, 127.5, 126.9, 126.9, 126.4(2*CH), 126.3, 125.8, 125.7, 124.1, 31.0, 30.8, 28.9, 21.9.

**HRMS** (APCI pos) calcd $C_{29}H_{23}OS$ $[M+H]^+$ : 419.1464, found 419.1501.

**9-(5-bromo-2-methyl-2,3-dihydro-1H-cyclopenta[a]naphthalen-1-ylidene)-9H-thioxanthene (5').**

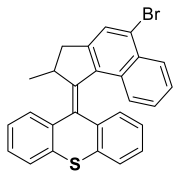


The compound was synthesized by a reported procedure as a yellow solid.[84]

**$^{1}$H NMR** (400 MHz, $CDCl_3$) δ 8.11 (dd, $J$ = 8.6, 1.2 Hz, 1H), 7.82 – 7.75 (m, 2H), 7.60 (ddd, $J$ = 14.9, 7.8, 1.3 Hz, 2H), 7.34 (td, $J$ = 7.6, 1.3 Hz, 1H), 7.26 – 7.20 (m, 2H), 7.00 (td, $J$ = 7.6, 1.5 Hz, 1H), 6.93 (dd, $J$ = 8.5, 1.2 Hz, 1H), 6.82 (ddd, $J$ = 8.3, 6.7, 1.3 Hz, 1H), 6.69 (dd, $J$ = 7.7, 1.4 Hz, 1H), 6.61 (td, $J$ = 7.5, 1.2 Hz, 1H), 4.31 (p, $J$ = 6.7 Hz, 1H), 3.65 (dd, $J$ = 15.6, 6.2 Hz, 1H), 2.61 (d, $J$ = 15.6 Hz, 1H), 0.79 (d, $J$ = 6.8 Hz, 4H).

**$^{13}$C NMR** (101 MHz, $CDCl_3$) δ 146.1, 145.3, 140.1, 137.8, 135.8, 135.8, 135.6, 131.1, 129.9, 129.4, 128.7, 128.1, 127.9, 127.7, 127.7, 127.0, 126.7, 126.6, 126.6, 126.4, 126.3, 125.7, 125.6, 124.4, 39.7, 38.0, 19.5.

**HRMS** (APCI pos) calcd $C_{27}H_{20}BrS$ $[M+H]^+$ : 455.0464, found 455.0437.

**2-methyl-1-(9H-thioxanthen-9-ylidene)-2,3-dihydro-1H-cyclopenta[a]naphthalene-5-carbaldehyde (5)**.

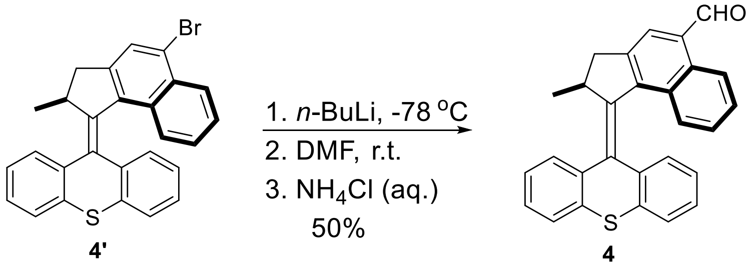


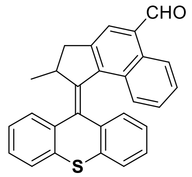


Under a $N_2$ atmosphere, 9-(5-bromo-2-methyl-2,3-dihydro-1H-cyclopenta[a]naphthalen-1-ylidene)-9H-thioxanthene (91.0 mg, 0.2 mmol) was dissolved in THF (10 mL) and cooled to -78 °C. Then, n-butyllithium (1.6M in hexane, 0.16 mL, 0.25 mmol) was slowly added to the solution and the mixture was allowed to stir for 15 min. Next, anhydrous DMF (0.25 mL) was added, and the mixture was allowed to stir for 1 h at room temperature. The reaction was quenched by saturated aqueous $NH_4Cl$. The mixture was extracted with EtOAc, washed with water, brine, dried over $Na_2SO_4$, filtrated and concentrated in vacuo. The crude product was purified by column chromatography ($SiO_2$, pentane:EtOAc = 5:1) to afford motor **5** as a yellow solid (20.9 mg, 0.084 mmol, 42%).

**$^1$H NMR** (400 MHz, $CDCl_3$) δ 10.41 (s, 1H), 9.04 (d, *J* = 8.5 Hz, 1H), 7.95 (s, 1H), 7.81 (d, *J* = 7.8 Hz, 1H), 7.70 – 7.49 (m, 2H), 7.35 (tdd, *J* = 6.6, 5.1, 1.3 Hz, 2H), 7.26 (d, *J* = 7.4 Hz, 1H), 7.07 – 6.97 (m, 2H), 6.87 (ddd, *J* = 8.4, 6.8, 1.3 Hz, 1H), 6.66 – 6.55 (m, 2H), 4.38 (p, *J* = 6.7 Hz, 1H), 3.73 (dd, *J* = 15.5, 6.2 Hz, 1H), 2.73 (d, *J* = 15.5 Hz, 1H), 0.82 (d, *J* = 6.9 Hz, 3H).

**$^{13}$C NMR** (101 MHz, $CDCl_3$) δ 193.0, 145.1, 144.1, 143.1, 139.5, 137.3, 135.7, 135.5, 133.6, 132.3, 131.9, 130.6, 129.3, 128.8, 128.0, 127.7, 127.5, 127.3, 126.9, 126.6, 126.6, 126.6, 126.5, 125.5, 124.5, 39.5, 37.8, 19.5.

**HRMS** (APCI pos) calcd $C_{28}H_{21}OS$ $[M+H]^+$ : 405.1308, found 405.1314.

## THI processes of motors

Solutions of motors **1**, **2** and **4** were irradiated at appropriate wavelength until no further changes were observed in the UV-Vis spectra, that is, the photostationary state was reached. The kinetics of the thermal step of the rotary cycle of molecular motors in the range of temperatures was followed with UV-Vis spectroscopy and fitted with an exponential decay $A = A_1\exp(-k^{-1}t) + C$ using Origin software. The Eyring plot method was used to calculate the Gibbs free energy of activation ($\Delta^{\ddagger}G$(20 °C)), rate constants ($k$) and half-lives ($t_{1/2}$) at room temperature (20 °C). Uncertainties in $\Delta^{\ddagger}G$ at room temperature are provided as 95% confidence intervals.

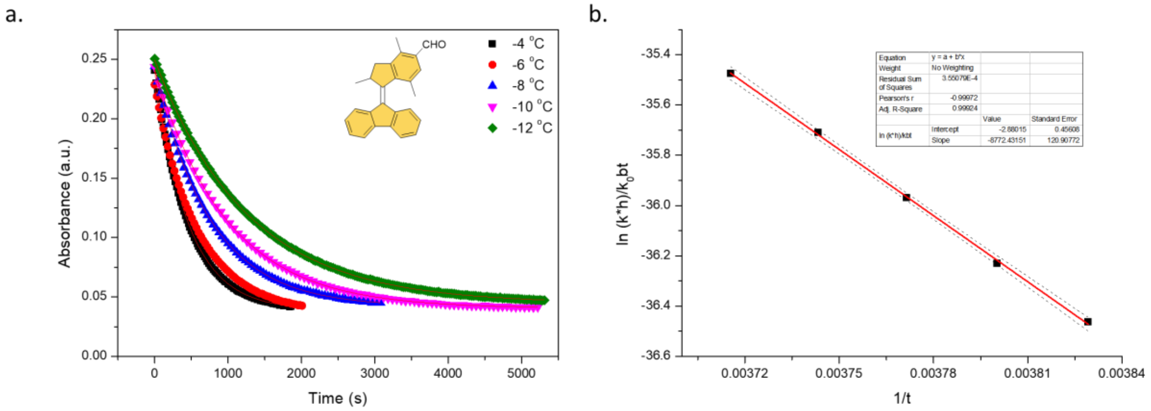


**Figure S1. Thermal isomerization of motor 1. a)** Kinetic study for the thermal isomerization step of motor **1** from metastable isomer to stable isomer in $CH_2Cl_2$; b) Eyring plots of THI isomerization for motor **1**. Dashed lines indicate 95% confidence intervals.

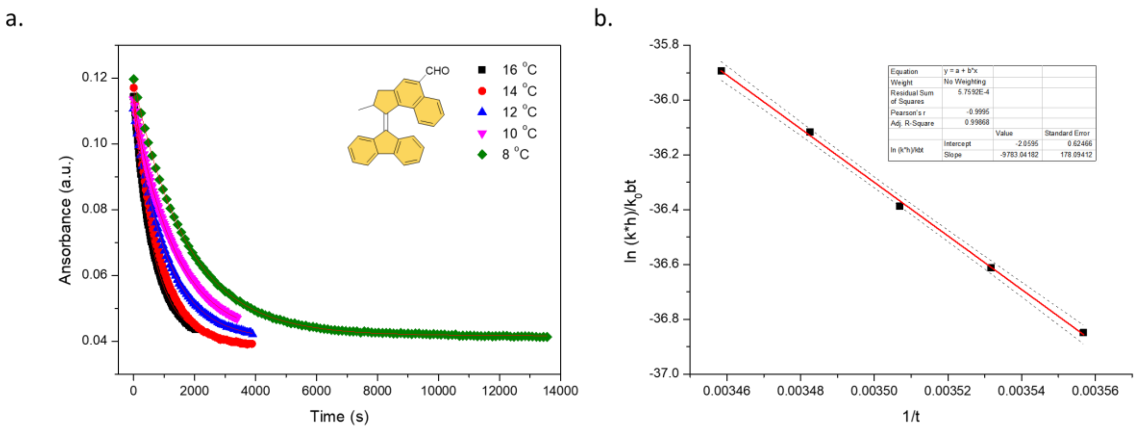


**Figure S2. Thermal isomerization of motor 2. a)** Kinetic study for the thermal step of motor **2** from metastable isomer to stable isomer in $CH_2Cl_2$; b) Eyring plots of THI isomerization for motor **2**. Dashed lines indicate 95% confidence intervals.

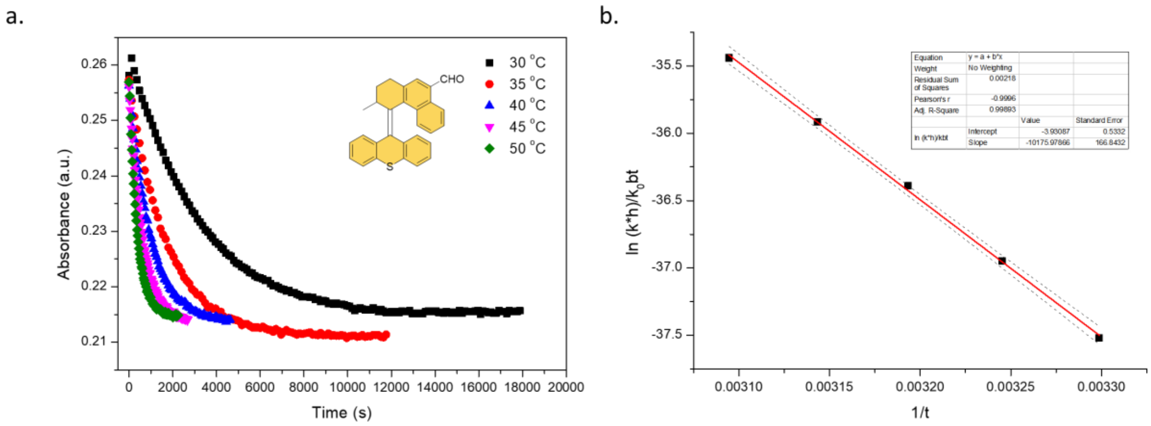


**Figure S3. Thermal isomerization of motor 4.** a) Kinetic study for the thermal step of motor **4** from metastable isomer to stable isomer in THF; b) Eyring plots of THI isomerization of for motor **4**. Dashed lines indicate 95% confidence intervals.

## Photoisomerization of motors and switch recorded via UV-Vis spectroscopy

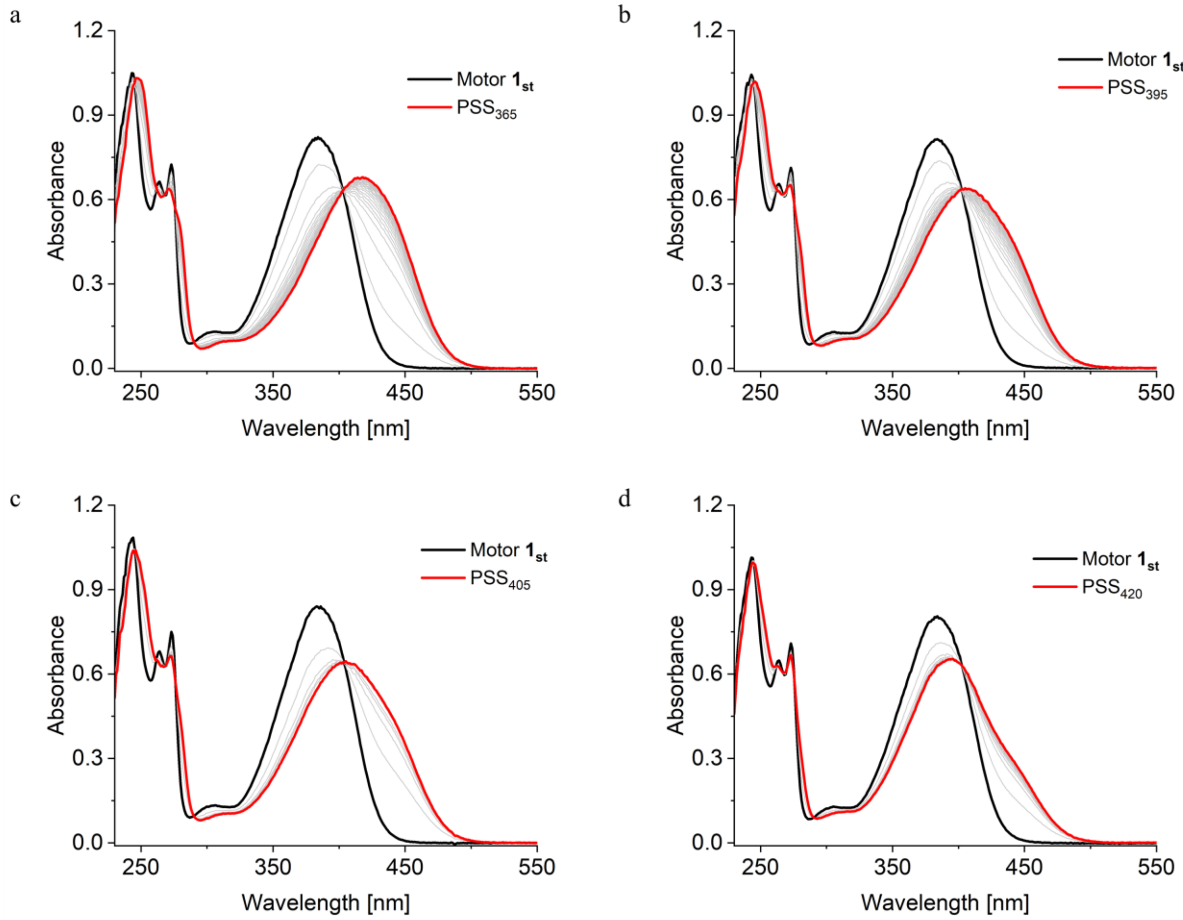


**Figure S4. Photochemical isomerization of motor 1 in solution.** Changes in the UV-Vis spectra of motor $\mathbf{1}_{st}$ ($CH_2Cl_2$, 0 °C) upon irradiation at a) 365nm; b) 395 nm; c) 405nm and d) 420nm.

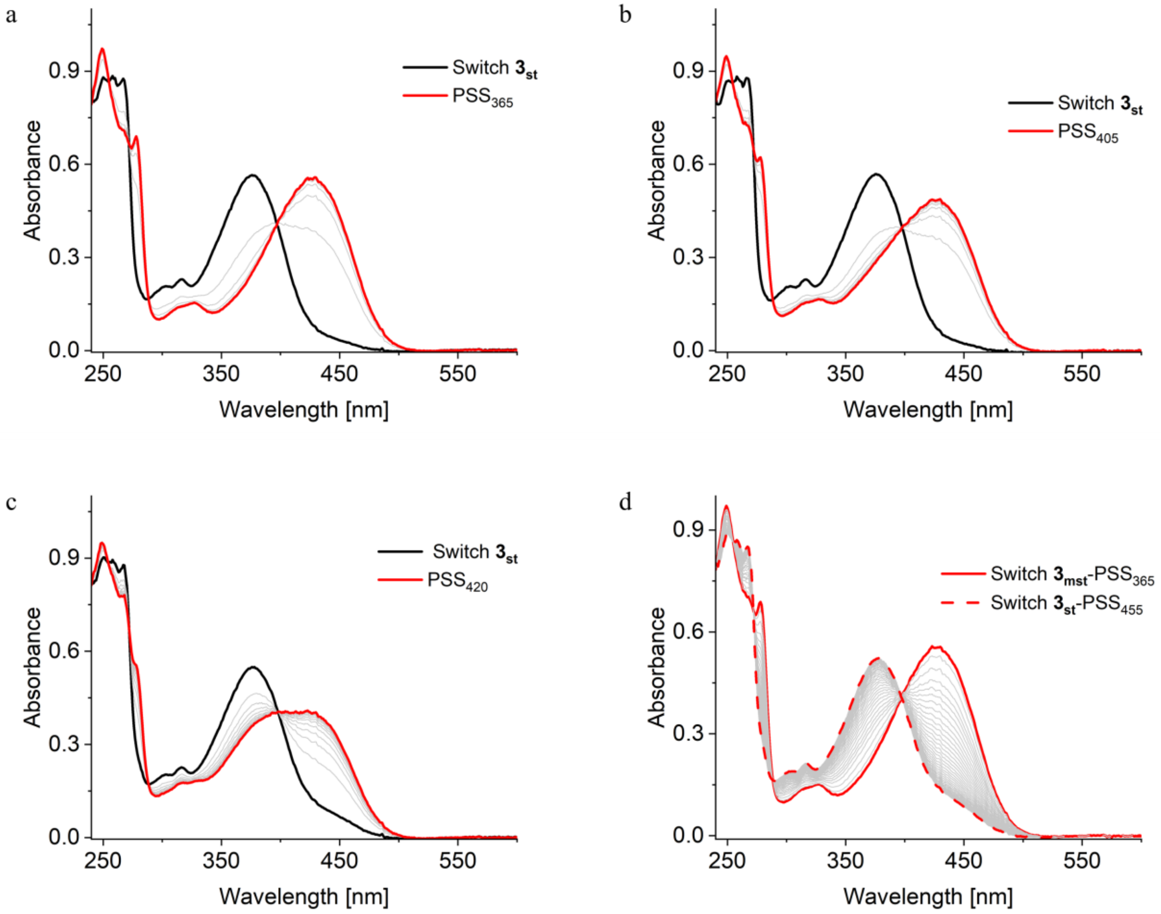


**Figure S5 Photochemical isomerization of motor 3 in solution.** Photoisomerization of switch $\mathbf{3_{st}}$. Changes in the UV-Vis spectra of motor $\mathbf{3_{st}}$ ($CH_2Cl_2$, 0 °C) upon irradiation at. a) 365 nm b) 405 nm; c) 420 nm and d) 455 nm for back isomerization from $\mathbf{3_{mst}}$ to $\mathbf{3_{st}}$.

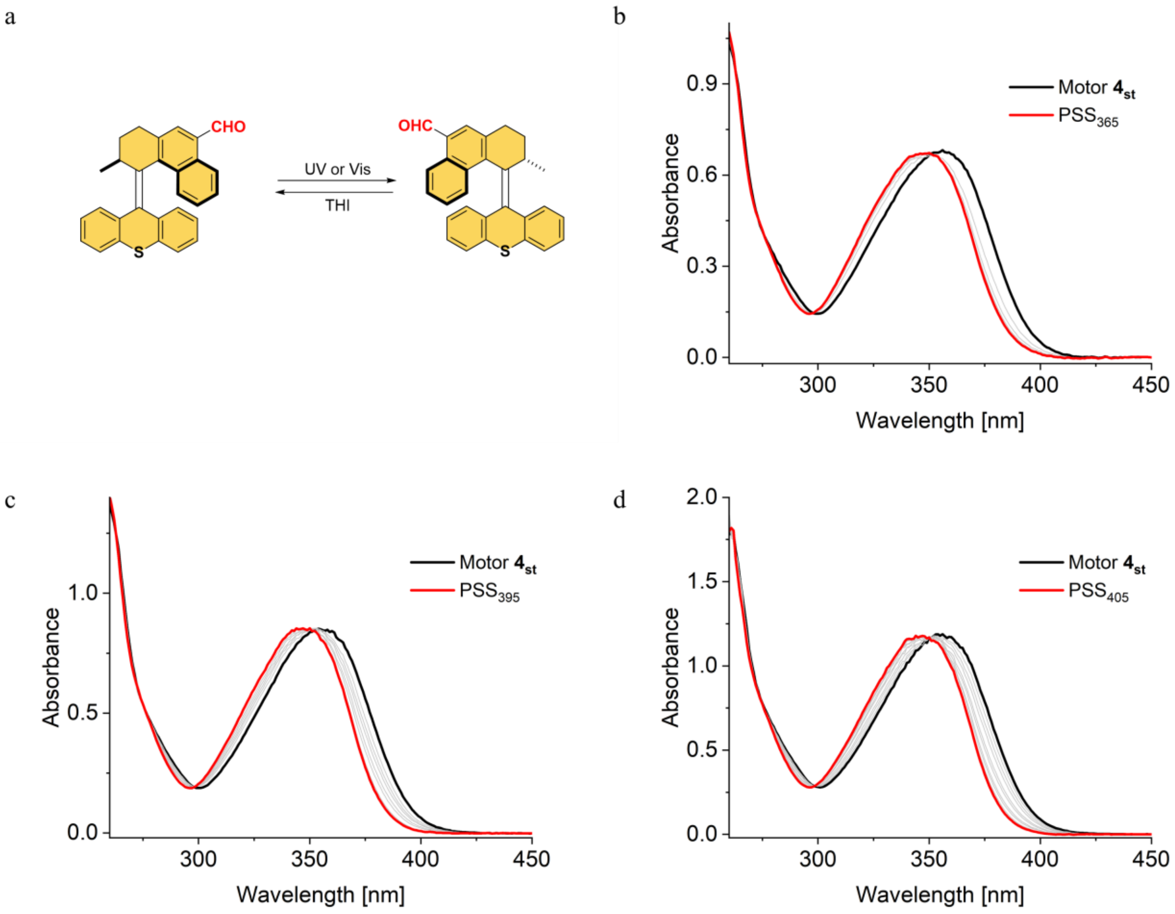


**Figure S6. Photochemical isomerization of motor 4 in solution.** a) Molecular rotation of motor $\mathbf{4}_{\mathbf{st}}$. Changes in the UV-Vis spectra of motor $\mathbf{4}_{\mathbf{st}}$ ($CH_2Cl_2$, 0 °C) upon irradiation at b) 365nm; c) 395 nm and d) 405 nm.

NMR irradiation studies of motors

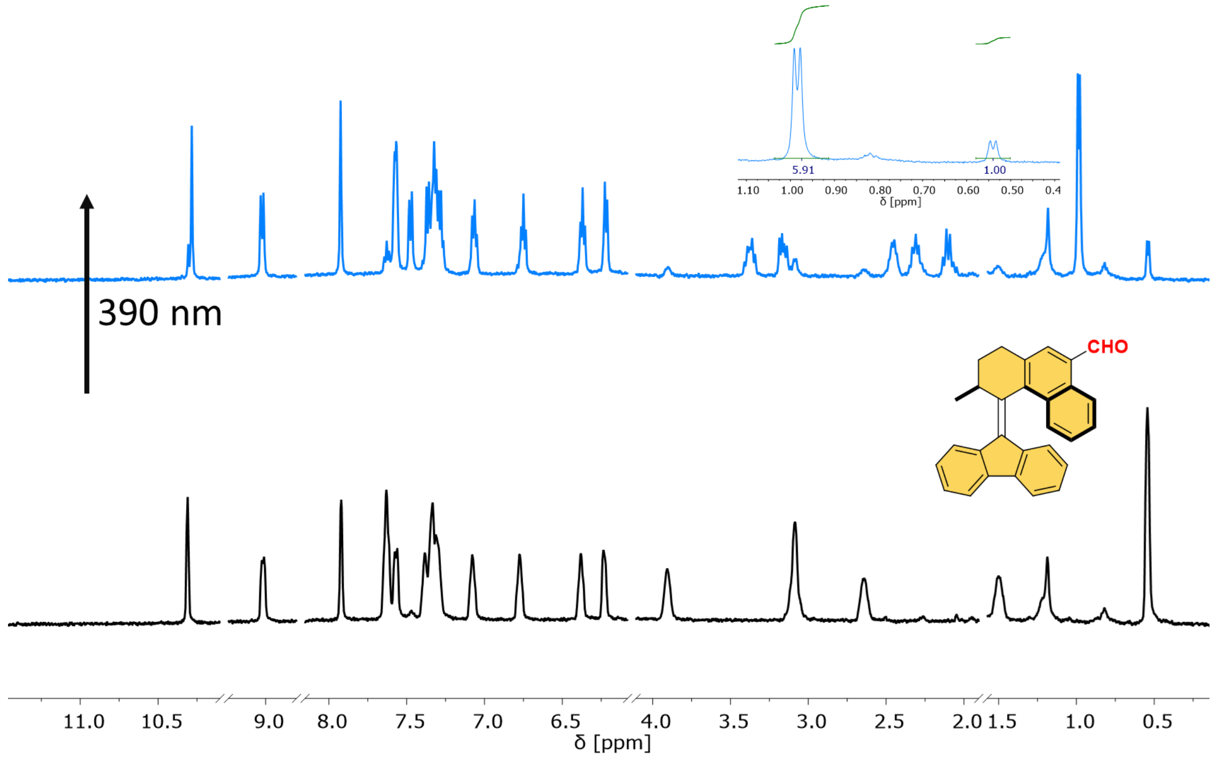


**Figure S7. NMR study of isomerization of bistable switch 3.** Changes in the $^1$H NMR spectra of bistable switch **3$_{st}$** in $CD_2Cl_2$ at -50 °C upon irradiation at 390 nm (black spectra to light blue spectra). A PSS ratio of 14:86 (metastable:stable) was calculated by integration of the resonances characteristic of the respective isomers.

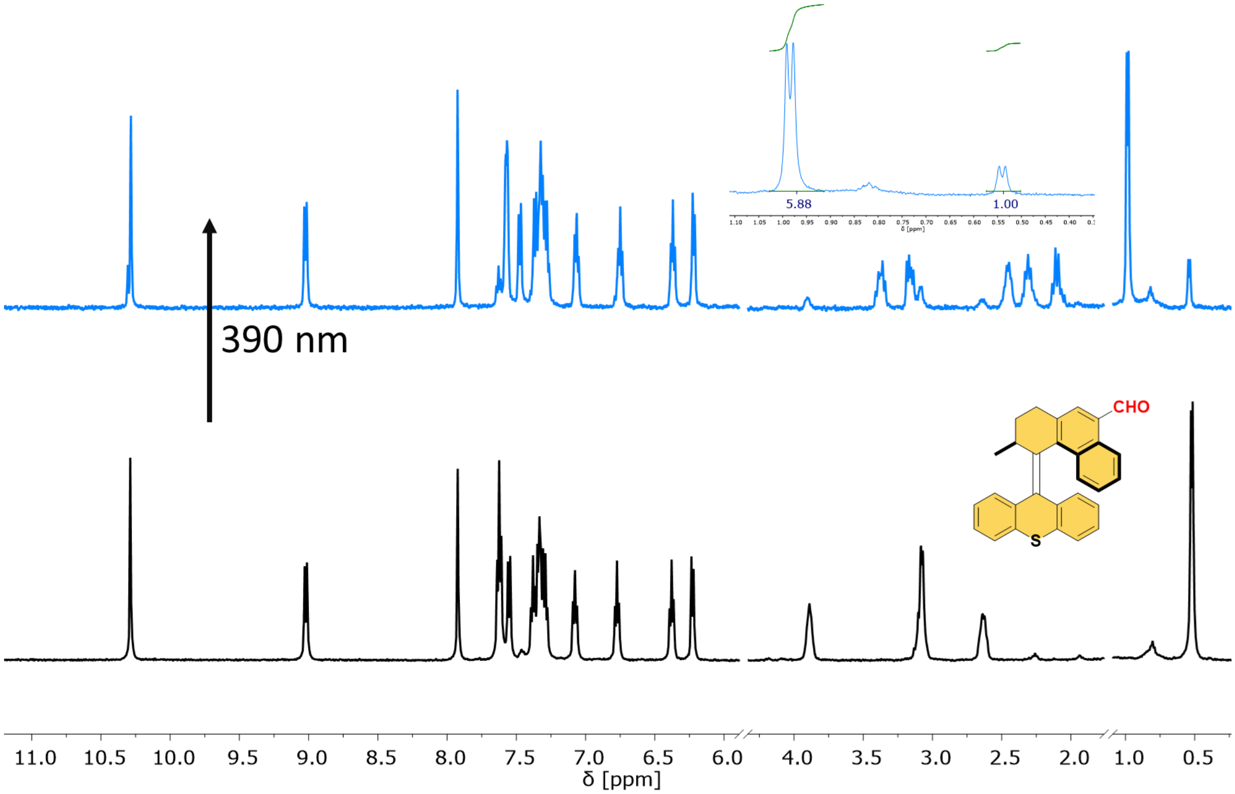


**Figure S8. NMR study of isomerization of motor 4.** Changes in the $^1$H NMR spectra of motor $\mathbf{4_{st}}$ in $CD_2Cl_2$ at -60 °C upon irradiation at 390 nm (black spectra to light blue spectra). A PSS ratio 15:85 (metastable:stable) was calculated by integration of the resonances characteristic of the respective isomers.

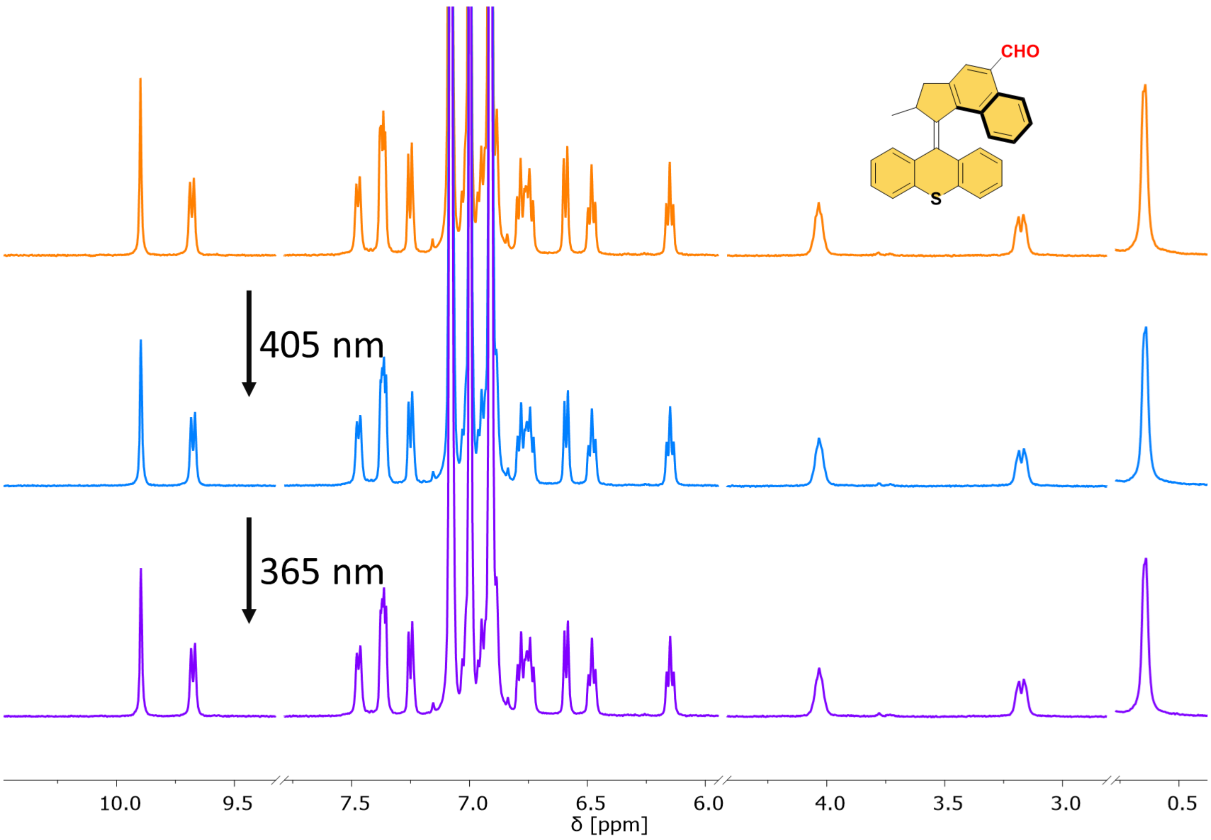


**Figure S9. NMR study of isomerization of motor 5.** Changes in the $^{1}$H NMR spectra of motor $\mathbf{5_{st}}$ in $d_8$-toluene at -80 °C. No spectral changes were observed from orange (initial) to light blue (405 nm irradiation) to purple spectra (365 nm irradiation).

<u>Quantum yield determination</u>

Ferrioxalate Chemical Actinometry

A modification of a standard protocol was applied for the determination of the photon flux.[85,86] An aqueous $H_2SO_4$ solution (0.05 M) containing freshly recrystallized $K_3[Fe(C_2O_4)_3]$ (41 mM, 2.0 mL, 1 cm quartz cuvette) was irradiated at 20 °C for a given period of time in the dark with a 365 nm LED. The solution was then diluted with 1.0 mL of an aqueous $H_2SO_4$ solution (0.5 M) containing phenanthroline (1 g/L) and NaOAc (122.5 g/L) and left to react for 10 min. The absorption at $\lambda$= 510 nm was measured and compared to an identically prepared non-irradiated sample. The concentration of $[Fe(phenanthroline)_3]^{2+}$ complex was calculated using its molar absorptivity ($\varepsilon$ = 11100 $M^{-1}$ $cm^{-1}$) and considering the dilution. The quantity of $Fe^{2+}$ ions expressed in mol was plotted versus time (expressed in seconds, s) and the slope, obtained by linear fitting the data points to the equation y = ax + b, equals the rate of formation of the $Fe^{2+}$ ion at the given wavelength. This rate can be converted into the photon flux (I) by dividing it by the quantum yield of the $[Fe(C_2O_4)_3]^{2+}$ complex at the wavelength of interest ($\Phi^{365nm}$ = 1.21, $\Phi^{390nm}$ = 1.28, $\Phi^{420nm}$ =1.24, $\Phi^{445nm}$ =1.18) and by the probability of photon absorption of the $Fe^{3+}$ complex (approximated to 1 as in all cases we were working in total absorption regime). The obtained molar photon fluxes were $I^{365nm} = 4.7\cdot10^{-5}$ mmol $s^{-1}$, $I^{390nm} = 3.85\cdot10^{-5}$ mmol $s^{-1}$, $I^{420nm} = 3.26\cdot10^{-5}$ mmol $s^{-1}$, $I^{445nm} = 2.60\cdot10^{-5}$ mmol $s^{-1}$.

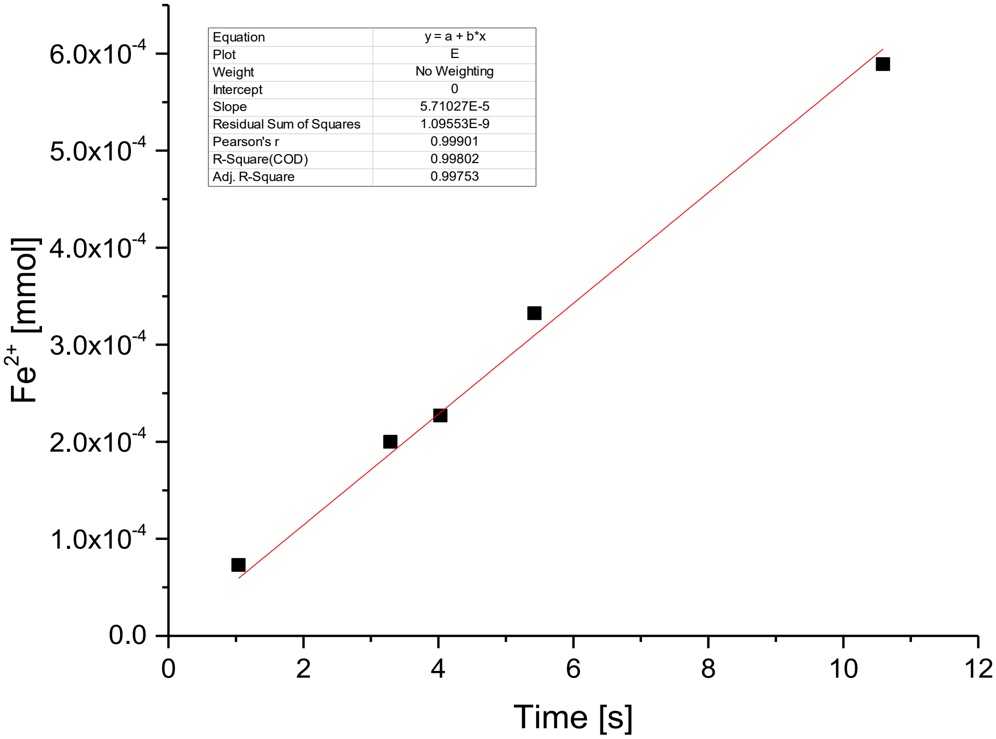


**Figure S10. Determination of photon flux at 365 nm.** Linear fitting of the $Fe^{2+}$ moles generated upon irradiation of the $[Fe(C_2O_4)_3]^{2+}$ complex upon irradiation at 365 nm with different irradiation times.

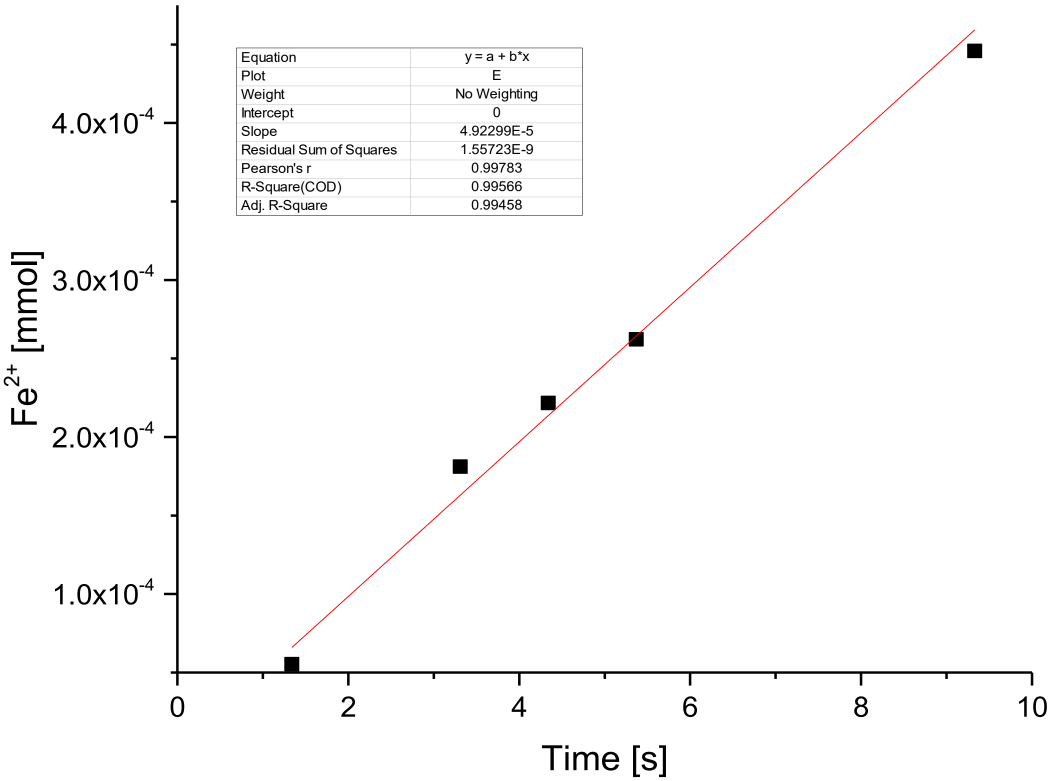


**Figure S11. Determination of photon flux at 390 nm.** Linear fitting of the $Fe^{2+}$ moles generated upon irradiation of the $[Fe(C_2O_4)_3]^{2+}$ complex upon irradiation at 390 nm with different irradiation times.

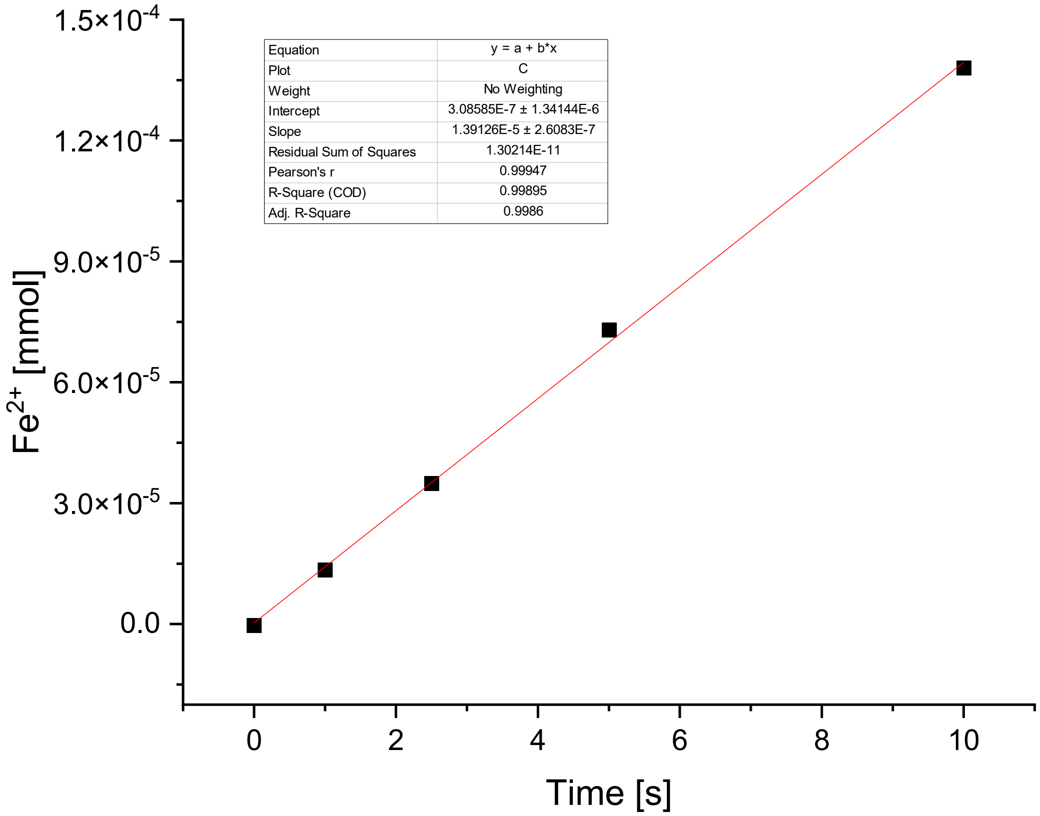


**Figure S12. Determination of photon flux at 420 nm.** Linear fitting of the $Fe^{2+}$ moles generated upon irradiation of the $[Fe(C_2O_4)_3]^{2+}$ complex upon irradiation at 420 nm with different irradiation times.

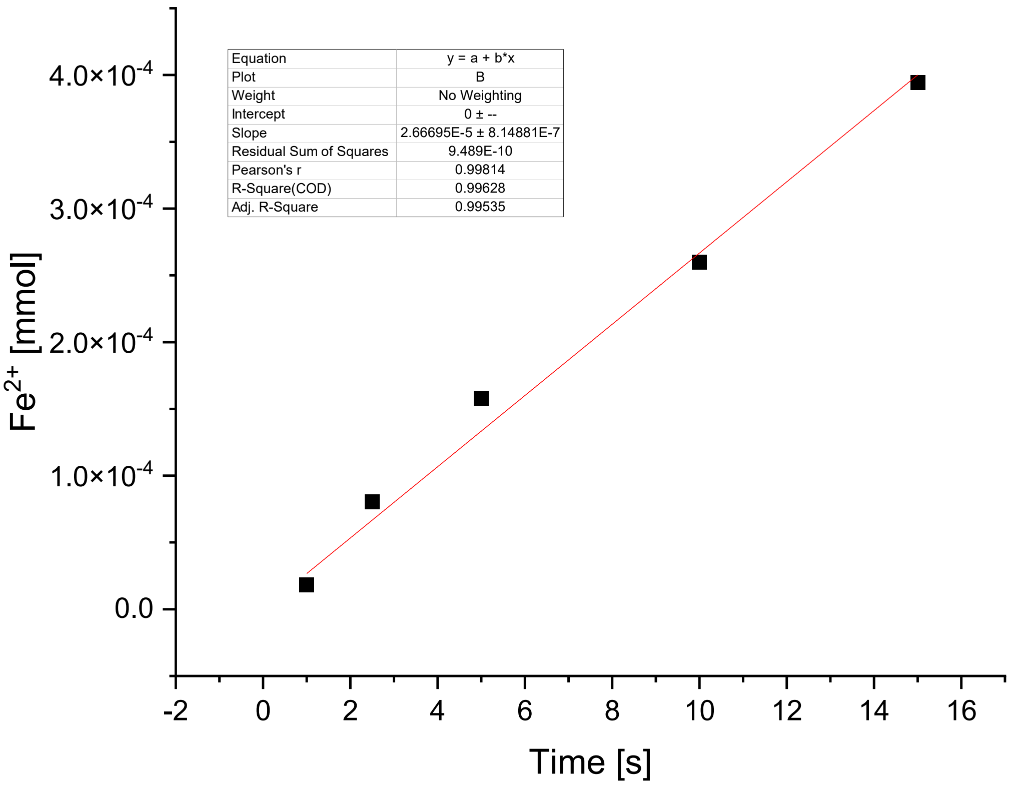


**Figure S13. Determination of photon flux at 455 nm.** Linear fitting of the $Fe^{2+}$ moles generated upon irradiation of the $[Fe(C_2O_4)_3]^{2+}$ complex upon irradiation at 445 nm with different irradiation times.

Solutions of the stable state of a specific motor (S) in dichloromethane (40–50 µM, to obtain an absorbance of ca. 1 at the wavelength of irradiation) were irradiated with a LED of a selected wavelength (*i.e.* 390, 365, 300 nm) to lead to the formation of the metastable state (M). The spectra were collected following the evolution of the absorption at the wavelength of irradiation; baseline corrections were carried out to correct for baseline drifting, after which the data subsequently fitted using COPASI 4.29[88] following the same approach developed by Stranius & Börjesson.[12] Equiv. 14 in the original article was used to determine both approximated QYs ($QY_{SM}$ for the formation of the metastable state form the stable and $QY_{MS}$ for the opposite photochemical reaction). The kinetic constant $k_{MS'}$ related to the thermal formation of the next stable state via THI was considered in the equation for the analysis of the 2nd generation motor. $I$ is the photon flux, previously determined with ferrioxalate actinometry, $N_A$ the Avogadro number, $V$ the total volume of the irradiated solution (2 mL) and $\beta$ the fractions of photons absorbed by either the stable or the metastable state. The decay was fitted by the ODE solver present in COPASI, using a Levenberg-Marquardt algorithm with randomized initial conditions. To obtain physically sound results, the boundaries for the QYs values were fixed between $1 \cdot 10^{-6}$ and 1. The measurements were triplicated and averaged to afford the values presented in Table 2 and 3.

$$\frac{d[S]}{dt} = -\frac{QY_{SM} \cdot I \cdot \beta_S(t)}{N_A \cdot V} + \frac{QY_{MS} \cdot I \cdot \beta_M(t)}{N_A \cdot V} + k_{MS'}[MS]$$

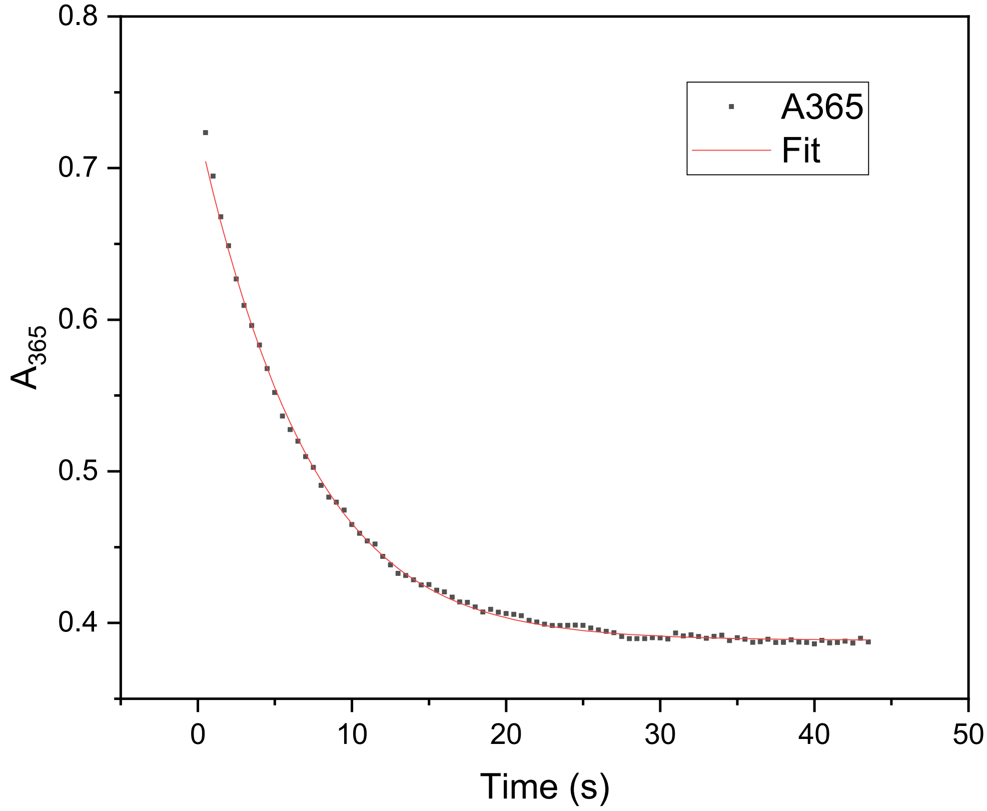


**Figure S14. Kinetic data for photochemical isomerization of motor 1.** Evolution of the absorbance at 365 nm upon irradiation of the motor $\mathbf{1_{st}}$ at 365 nm (black squares). The red line represents the fit obtained with the ODE solver from COPASI.

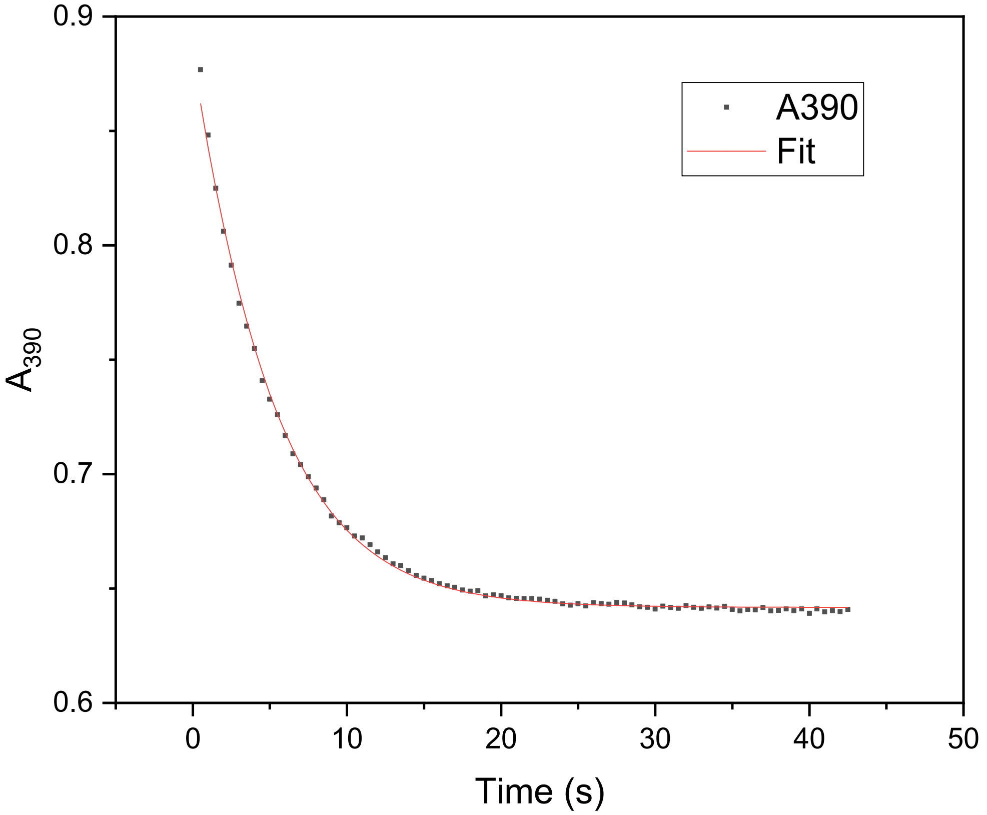


**Figure S15. Kinetic data for photochemical isomerization of motor 1.** Evolution of the absorbance at 390 nm upon irradiation of the motor $\mathbf{1}_{st}$ at 390 nm (black squares). The red line represents the fit obtained with the ODE solver from COPASI.

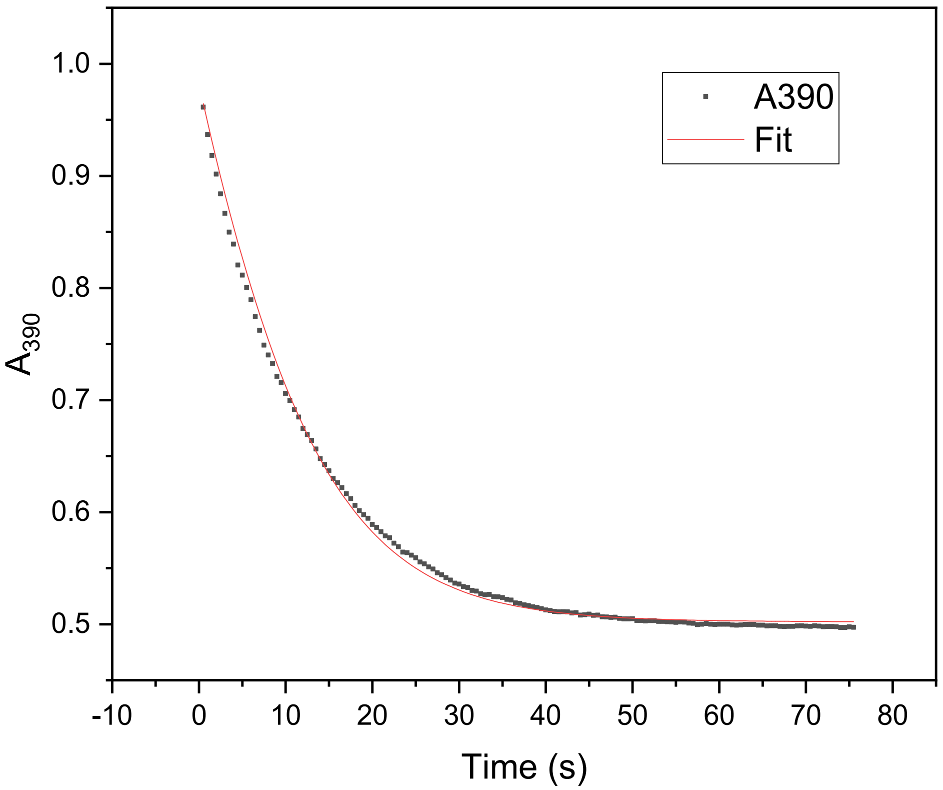


**Figure S16. Kinetic data for photochemical isomerization of motor 2.** Evolution of the absorbance at 390 nm upon irradiation of the motor $\mathbf{2}_{\mathbf{st}}$ at 390 nm (black squares). The red line represents the fit obtained with the ODE solver from COPASI.

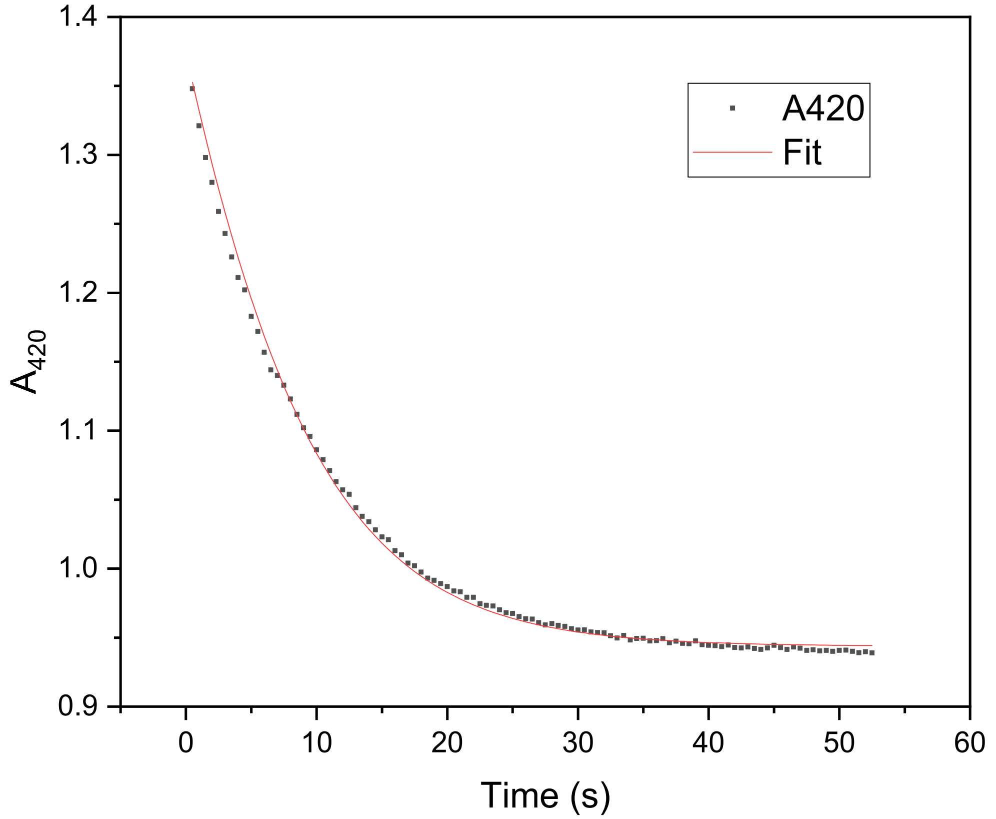


**Figure S17. Kinetic data for photochemical isomerization of motor 2.** Evolution of the absorbance at 420 nm upon irradiation of the motor $\mathbf{2_{st}}$ at 420 nm (black squares). The red line represents the fit obtained with the ODE solver from COPASI.

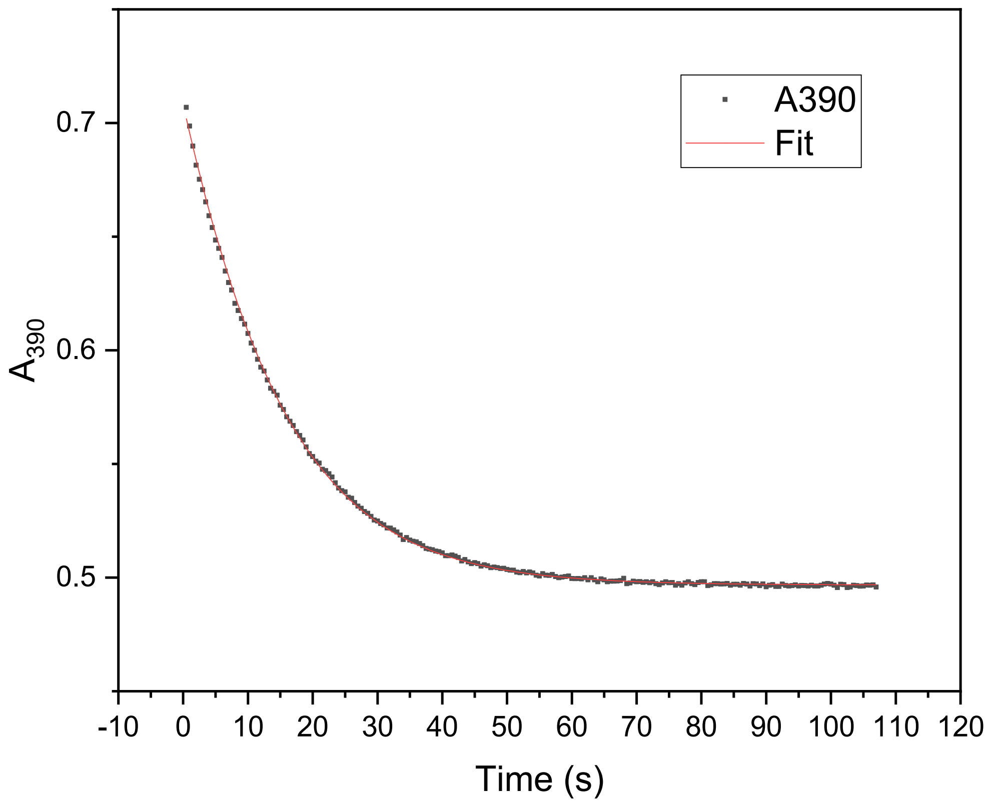


**Figure S18. Kinetic data for photochemical isomerization of bistable switch 3.** Evolution of the absorbance at 390 nm upon irradiation of the motor $\mathbf{3}_{st}$ at 390 nm (black squares). The red line represents the fit obtained with the ODE solver from COPASI.

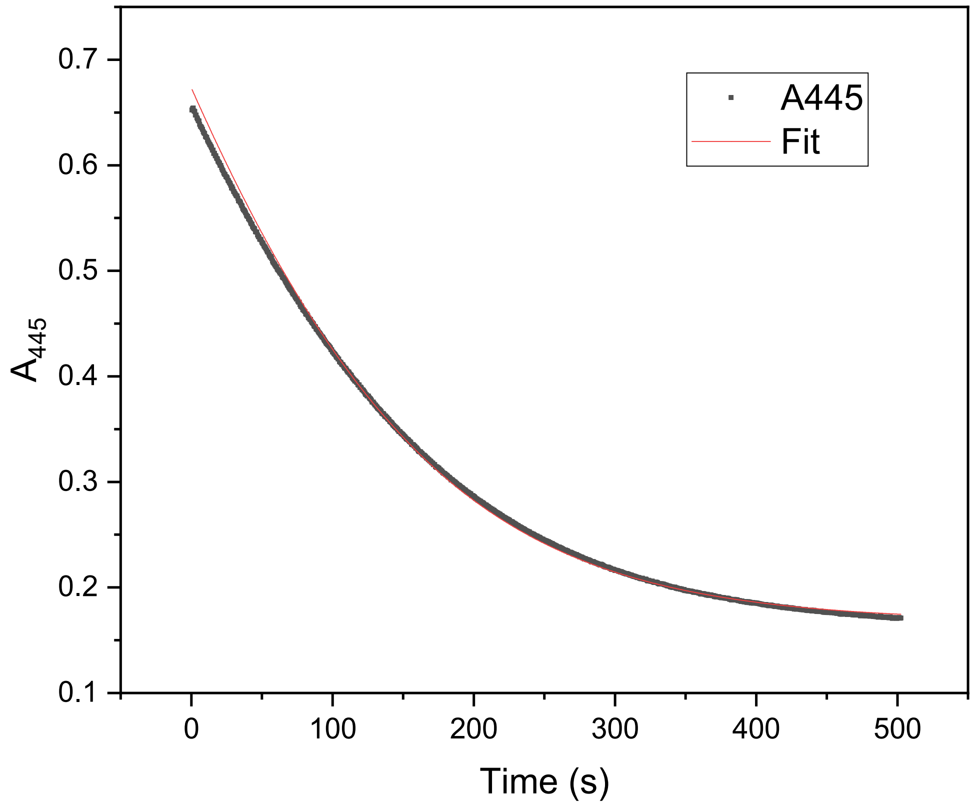


**Figure S19. Kinetic data for photochemical isomerization of bistable switch 3.** Evolution of the absorbance at 445 nm upon irradiation of the motor $\mathbf{3_{st}}$ at 445 nm (black squares). The red line represents the fit obtained with the ODE solver from COPASI.

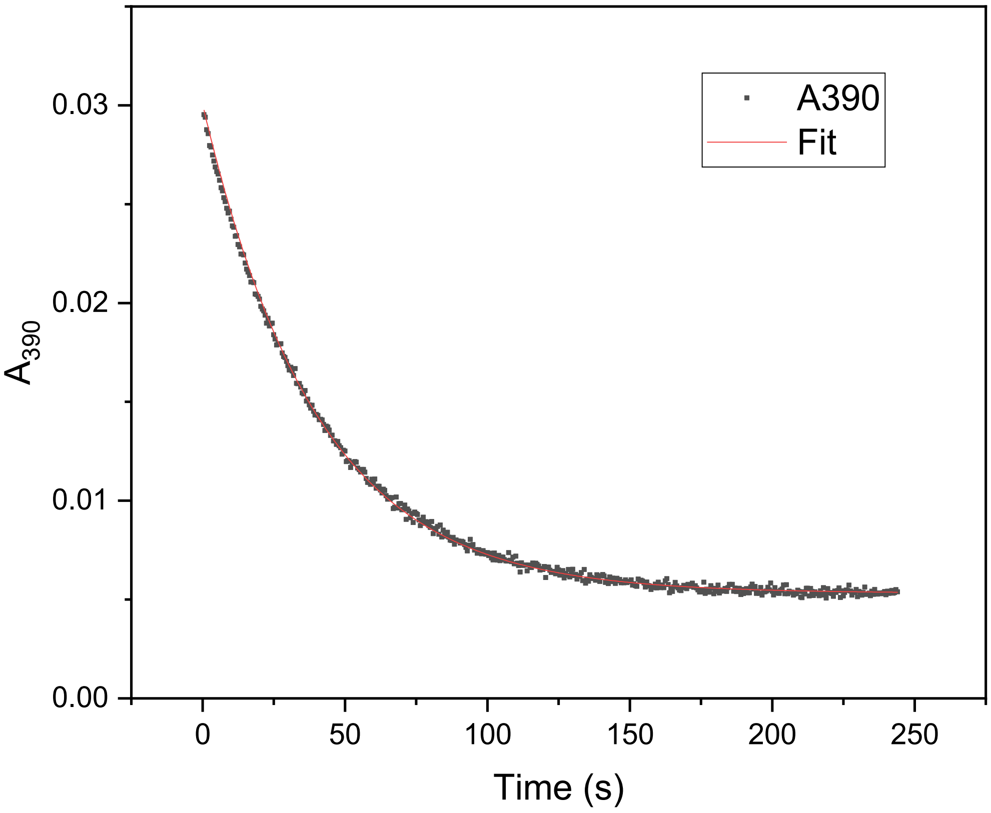


**Figure S20. Kinetic data for photochemical isomerization of motor 4.** Evolution of the absorbance at 390 nm upon irradiation of the motor $\mathbf{4_{st}}$ at 390 nm (black squares). The red line represents the fit obtained with the ODE solver from COPASI.

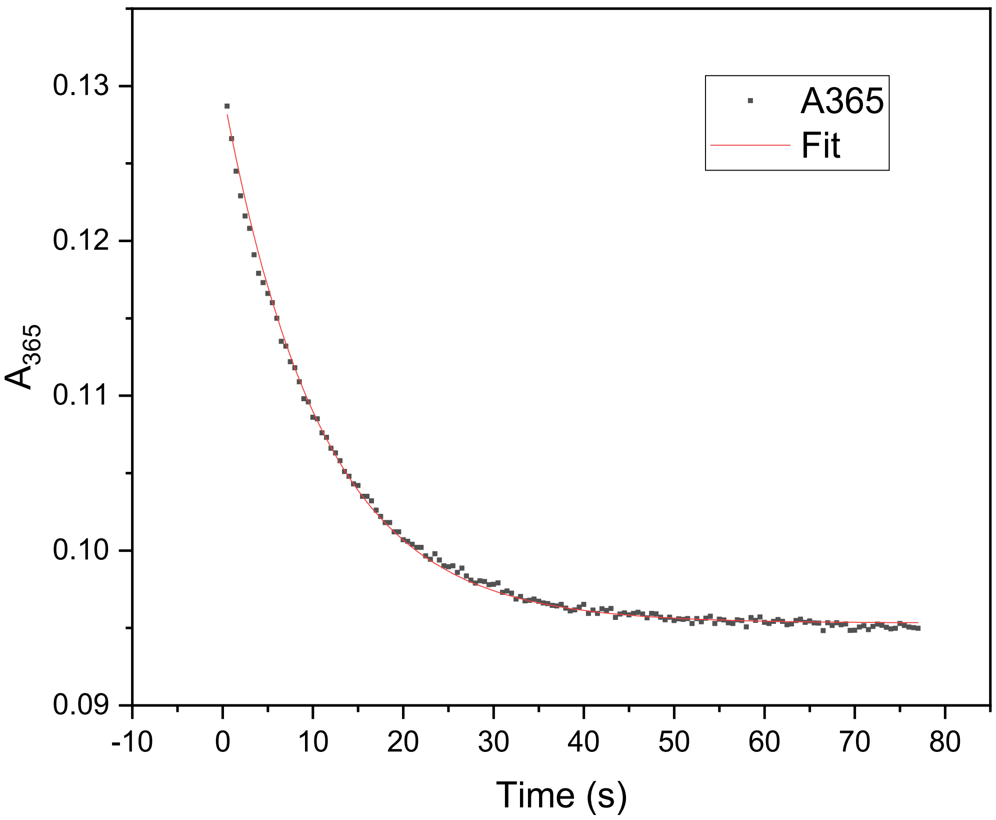


**Figure S21. Kinetic data for photochemical isomerization of motor 4.** Evolution of the absorbance at 365 nm upon irradiation of the motor $\mathbf{4_{st}}$ at 365 nm (black squares). The red line represents the fit obtained with the ODE solver from COPASI.

CD and UV spectra of enantiomerically pure motor

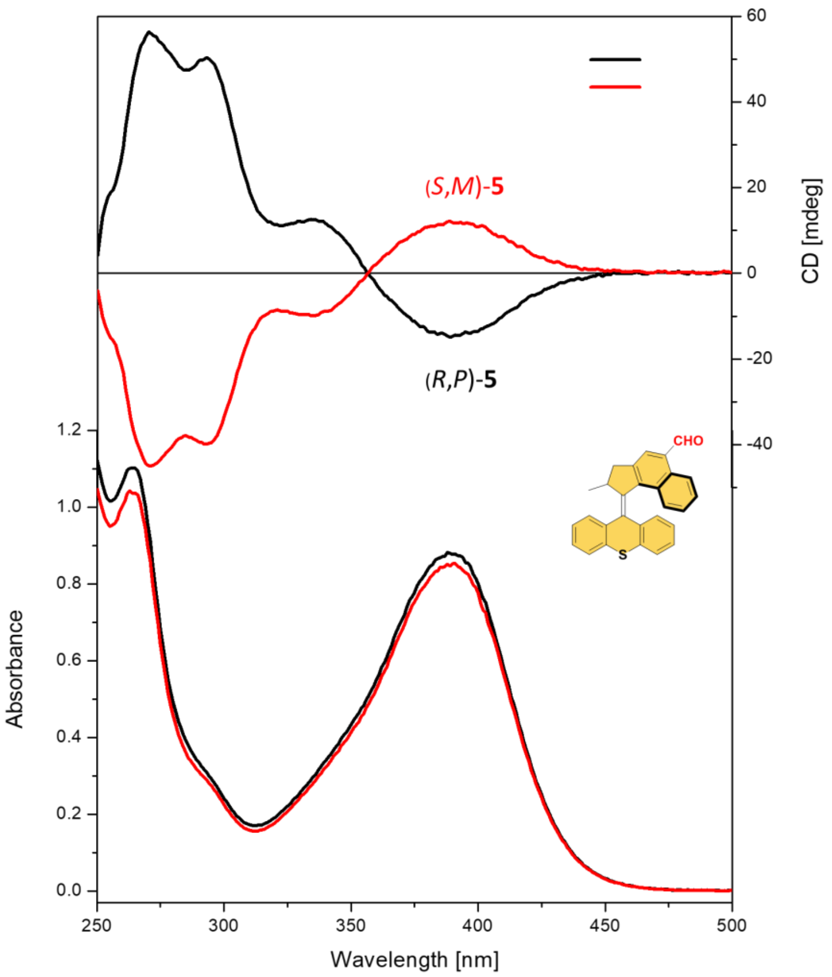


**Figure S22. Steady-state spectra of motor 5.** UV and CD spectra of (*S*,*M*) and (*R*,*P*) motor **5**$_{\mathbf{st}}$.

## Transient absorption spectroscopy

Nanosecond transient absorption spectra were recorded with an in-house assembled setup. A different excitation wavelength (390 – 420 nm) was used depending on the absorption spectrum of the sample. For motor **1**$_{st}$, **2**$_{st}$, and **2'**$_{st}$, a flow setup was used in which the solution was passed through a flow cuvette during the measurements. The excitation wavelength was generated using a tunable Nd:YAG-laser system (NT342B, Ekspla) comprising the pump laser (NL300) with harmonics generators (SHG, THG) producing 355 nm to pump an optical parametric oscillator (OPO) with SHG connected in a single device. The laser system was operated at a repetition rate of 10 Hz with a pulse length of 5 ns. The probe light running at 20 Hz was generated by a high-stability short arc xenon flash lamp (FX-1160, Excelitas Technologies) using a modified PS302 controller (EG&G). Using a 50/50 beam splitter, the probe light was split equally into a signal beam and a reference beam and focused (bi-convex lens 75mm) on the entrance slit of a spectrograph (SpectraPro-150, Princeton Instruments) with a grating of 150 ln/mm, blaze at 500 nm. The probe beam (A = 1 mm$^2$) was passed through the sample cell and orthogonally overlapped with the excitation beam on a 1 mm × 1 cm area. The excitation energy was recorded by measuring the excitation power at the back of an empty sample holder. In order to correct for fluctuations in the flash lamp spectral intensity, the reference was used to normalize the signal. Both beams were recorded simultaneously using a gated intensified CCD camera (PI-MAX3, Princeton Instruments) which has an adjustable gate of minimal 2.9 ns, normally a gate of 20 ns and software binning is used to improve the dynamic range and signal to noise ratio. Two delay generators (DG535 and DG645, Stanford Research Systems, Inc.) were used to trigger the excitation and to change the delay of the flash lamp together with the gate of the camera during the experiment. The setup was controlled by an in-house written Labview program. The transient data was treated via global analysis using Glotaran 1.5.1[89]

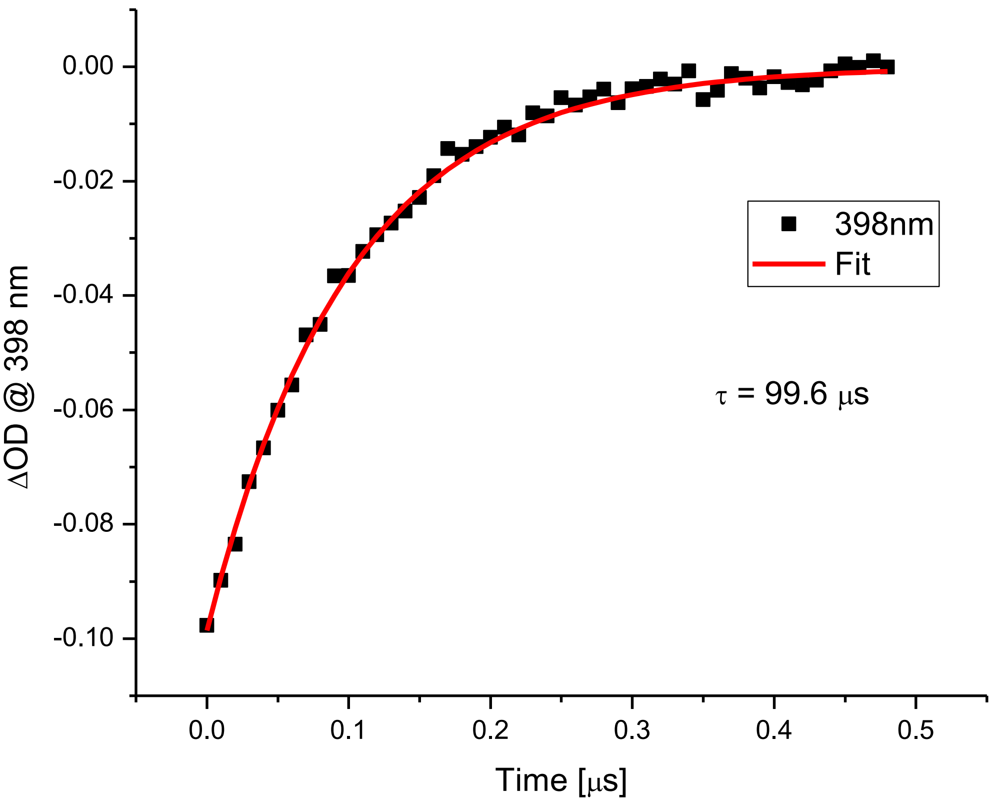


**Figure S23. Transient spectroscopy of motor 5.** Evolution of the absorption at 398 nm during the irradiation of Motor **5**$_{st}$ in DCM at 390 nm. The red line represents the fit obtained from Origin.

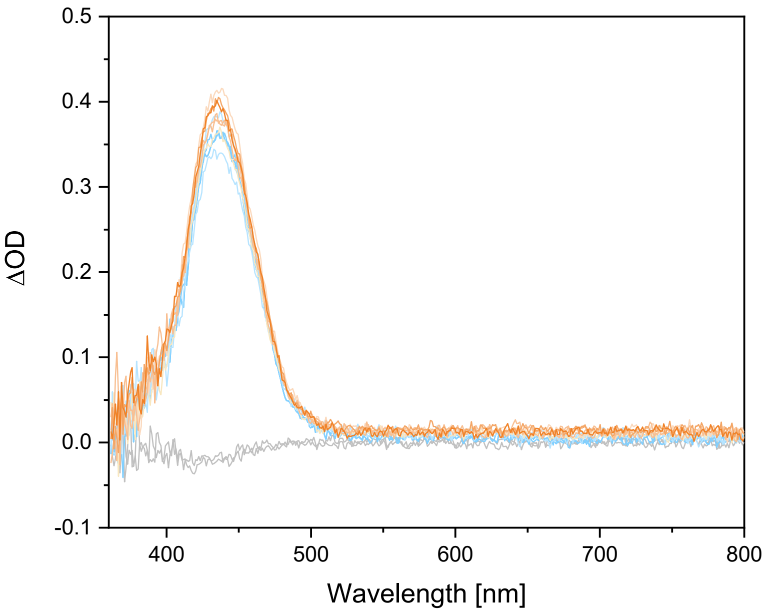


**Figure S24. Transient absorption of motor $1_{st}$ in $CH_2Cl_2$ at room temperature.** The sample was irradiated with a 410 nm light pulse upon which the spectrum was recorded in steps of 2 ns increasing delay until 20 ns. Baseline spectra before the laser pulse are shown in grey, and spectra after the laser pulse start from blue progressing to orange. An - on the time scale of the experiment - instantaneous appearance of an induced absorption band is observed corresponding to the absorption of the generated photoisomer.

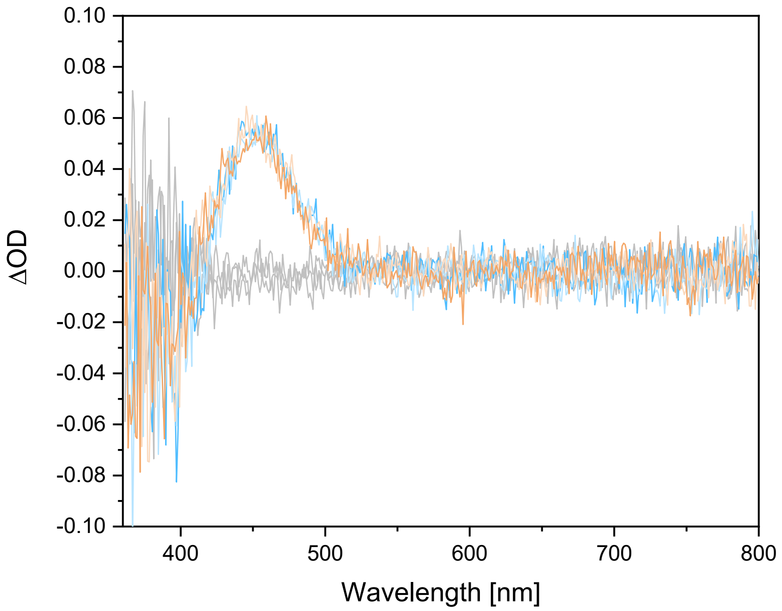


**Figure S25. Transient absorption of motor 2'$_{st}$ in $CH_2Cl_2$ at room temperature.** The sample was irradiated with a 395 nm light pulse upon which the spectrum was recorded in steps of 2 ns increasing delay until 8 ns. Baseline spectra before the laser pulse are shown in grey, and spectra after the laser pulse start from blue progressing to orange. An - on the time scale of the experiment - instantaneous appearance of an induced absorption band is observed corresponding to the absorption of the generated photoisomer.

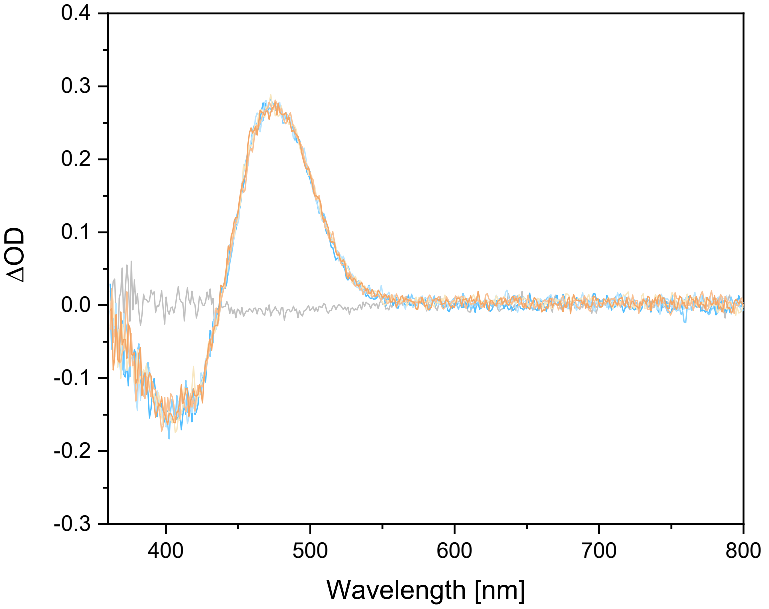


**Figure S26. Transient absorption of motor 2st in $CH_2Cl_2$ at room temperature.** The sample was irradiated with a 420 nm light pulse upon which the spectrum was recorded in steps of 2 ns increasing delay until 12 ns. Baseline spectra before the laser pulse are shown in grey, and spectra after the laser pulse start from blue progressing to orange. A bleach is observed in the absorption band of the initial isomer as well as an - on the time scale of the experiment - instantaneous appearance of an induced absorption band corresponding to the absorption of the generated photoisomer.

## X-ray structural analysis

Crystals motor **$1_{st}$** and motor **$2_{st}$** were obtained by slow evaporation from a concentrated solution in $CH_2Cl_2$. A single crystal was mounted on a cryoloop and analyzed on a Bruker-AXS D8 Venture diffractometer, using MoKα radiation (λ = 0.71073 Å). The data collection was done at room temperature under ambient conditions. The Bruker APEX4 software suite was used for data collection and processing., and a multi-scan absorption correction was applied using SADABS (SADABS-2016/2).[90] The structure was solved using SHELXT.[91] Subsequent refinement was done using SHELXL[92] in the OLEX2 software package.[93] Hydrogen atoms were generated by geometrical considerations and refined using a riding model.

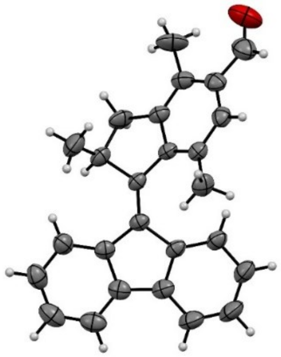

**Figure S27. Front-view ORTEP image of motor $1_{st}$.** Ellipsoids are drawn at 50% probability.

Crystallographic data for motor **$1_{st}$**

| | |
|---|---|
| Empirical formula | $C_{26}H_{22}O$ |
| Formula weight | 350.43 |
| Temperature/K | 293 |
| Crystal system | monoclinic |
| Space group | $P2_1/c$ |
| a/Å | 9.515(3) |
| b/Å | 11.800(3) |
| c/Å | 17.102(5) |
| α/° | 90 |
| β/° | 101.413(12) |
| γ/° | 90 |
| Volume/Å$^3$ | 1882.2(9) |
| Z | 4 |
| $\rho_{calc}$g/cm$^3$ | 1.237 |
| μ/mm$^{-1}$ | 0.073 |
| F(000) | 744.0 |
| Crystal size/mm$^3$ | 0.348 × 0.303 × 0.172 |
| Radiation | MoKα (λ = 0.71073) |
| 2Θ range for data collection/° | 5.718 to 59.146 |
| Index ranges | $-13 \leq h \leq 13$, $-16 \leq k \leq 16$, $-23 \leq l \leq 23$ |
| Reflections collected | 103833 |
| Independent reflections | 5268 [$R_{int}$ = 0.1712, $R_{sigma}$ = 0.0433] |
| Data/restraints/parameters | 5268/0/247 |
| Goodness-of-fit on F$^2$ | 1.194 |

| | |
|---|---|
| Final R indexes [I>=2σ (I)] | $R_1$ = 0.1141, $wR_2$ = 0.2113 |
| Final R indexes [all data] | $R_1$ = 0.1630, $wR_2$ = 0.2402 |
| Largest diff. peak/hole / e Å$^{-3}$ | 0.38/-0.30 |

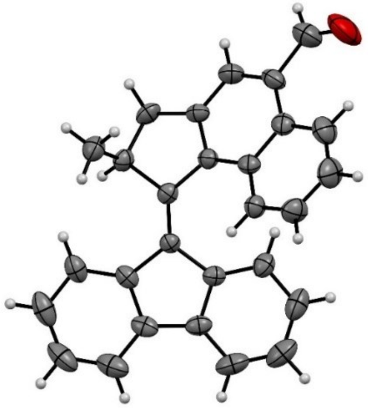

**Figure S28**. **Front-view ORTEP image of motor 2.** Ellipsoids are drawn at 50% probability.

<u>Crystallographic data for motor **2<sub></sub>**</u>

| | |
|---|---|
| Empirical formula | $C_{28}H_{20}O$ |
| Formula weight | 372.44 |
| Temperature/K | 293 |
| Crystal system | orthorhombic |
| Space group | Pbca |
| a/Å | 7.813(4) |
| b/Å | 20.156(10) |
| c/Å | 24.632(12) |
| α/° | 90 |
| β/° | 90 |
| γ/° | 90 |
| Volume/Å$^3$ | 3879(3) |
| Z | 8 |
| $\rho_{calc}$g/cm$^3$ | 1.276 |
| μ/mm$^{-1}$ | 0.076 |
| F(000) | 1568.0 |
| Crystal size/mm$^3$ | 0.206 × 0.157 × 0.026 |
| Radiation | MoKα (λ = 0.71073) |
| 2Θ range for data collection/° | 5.222 to 54.206 |
| Index ranges | $-10 \leq h \leq 10$, $-25 \leq k \leq 25$, $-31 \leq l \leq 31$ |
| Reflections collected | 118088 |
| Independent reflections | 4259 [$R_{int}$ = 0.2313, $R_{sigma}$ = 0.0396] |
| Data/restraints/parameters | 4259/0/263 |
| Goodness-of-fit on $F^2$ | 1.229 |
| Final R indexes [I>=2σ (I)] | $R_1$ = 0.1208, $wR_2$ = 0.1927 |
| Final R indexes [all data] | $R_1$ = 0.1683, $wR_2$ = 0.2159 |
| Largest diff. peak/hole / e Å$^{-3}$ | 0.28/-0.29 |

The following A- and B- type ALERTS were generated:

Alert level B

RINTA01_ALERT_3_B The value of Rint is greater than 0.18 Rint given 0.231

Response: The quality of the obtained crystals was rather low. Several attempts were made to crystallize the compound, but no crystals of better quality could be obtained.

PLAT020_ALERT_3_B The Value of Rint is Greater Than 0.12 ......... 0.231 Report

Response: The quality of the obtained crystals was rather low. Several attempts were made to crystallize the compound, but no crystals of better quality could be obtained.

## Computational analysis

Computational analysis was used to optimise the thermal steps of the molecular motors. Geometry optimisations were performed at the r$^2$SCAN-3c level of theory[94] using the ORCA 5.0.4 software.[95] Solvation was included in all calculations using the CPCM[96] model with parameters for $CH_2Cl_2$ as the solvent. The stationary points were confirmed using frequency calculations and evaluation of the number of imaginary frequencies (0 for minima and 1 for transition states). TD-DFT was performed at a ωB97X-D3/def2-TZVPP (performed with TDA) level of theory.[97-99] The XYZ coordinates of all optimised structures can be found in a separate additional file.

For motor **3**, two main pathways from the metastable isomer **3$_m$** to the stable isomer **3$_s$** were considered: a thermal helix inversion (THI) and a thermal *E/Z* isomerization (TEZI). The TEZI transition state was calculated using broken-symmetry DFT. Motor **4** behaved similarly to previously reported structural analogues.[100] The thermal relaxation from the metastable isomer **4$_m$** to the stable isomer **4$_s$** occurs via multiple steps, a ring flip of the lower half and the upper half slipping over the lower half. Hence, two possible thermal helix inversion pathways were considered in which these two steps happen in a different order. In pathway A, the upper half slips first past the lower half and then a ring flip in the lower half takes place. In pathway B, a lower half ring flip is followed by the upper half slipping over the lower half. The slippage of the upper half over the lower half was found to be the rate-determining step in both pathways (transition states TS2 and TS5 for pathways A and B, respectively).

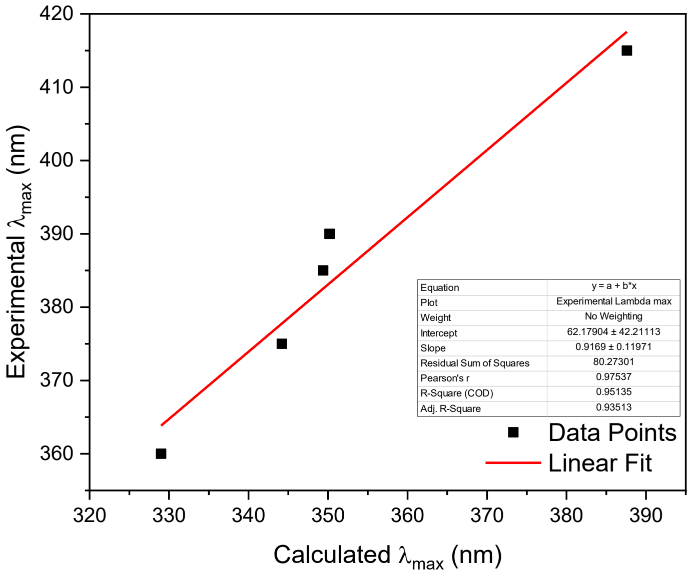


**Figure S29. Comparison of experimental and calculated excitation wavelengths for synthesized compounds.** Linear correction of calculated $\lambda_{max}$ (TD-DFT (ωB97X-D3/def2-TZVPP//r$^2$SCAN-3c/CPCM($CH_2Cl_2$)) using the calculated and experimental $\lambda_{max}$ values.

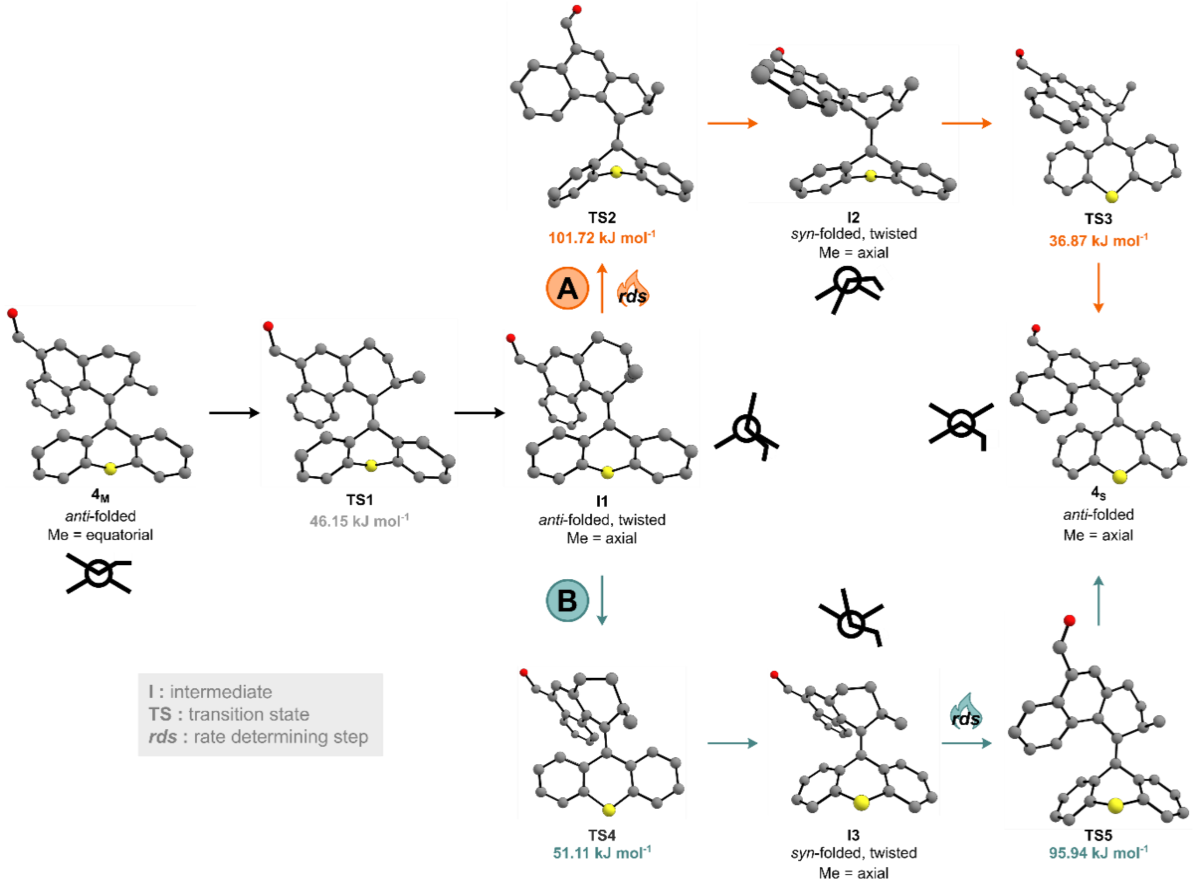


**Figure S30. Computational study of thermal isomerization pathway of motor 4.** Thermal conversion from metastable **4**$_{\mathbf{M}}$ to stable **4**$_{\mathbf{S}}$ via pathways A (top, orange) and B (bottom, blue). With all intermediates a schematic top view of the molecule along the C-C double bond is drawn. The rate-determining ring flip in the upper half is indicated with rds. The energies are referenced to **4**$_{\mathbf{M}}$.

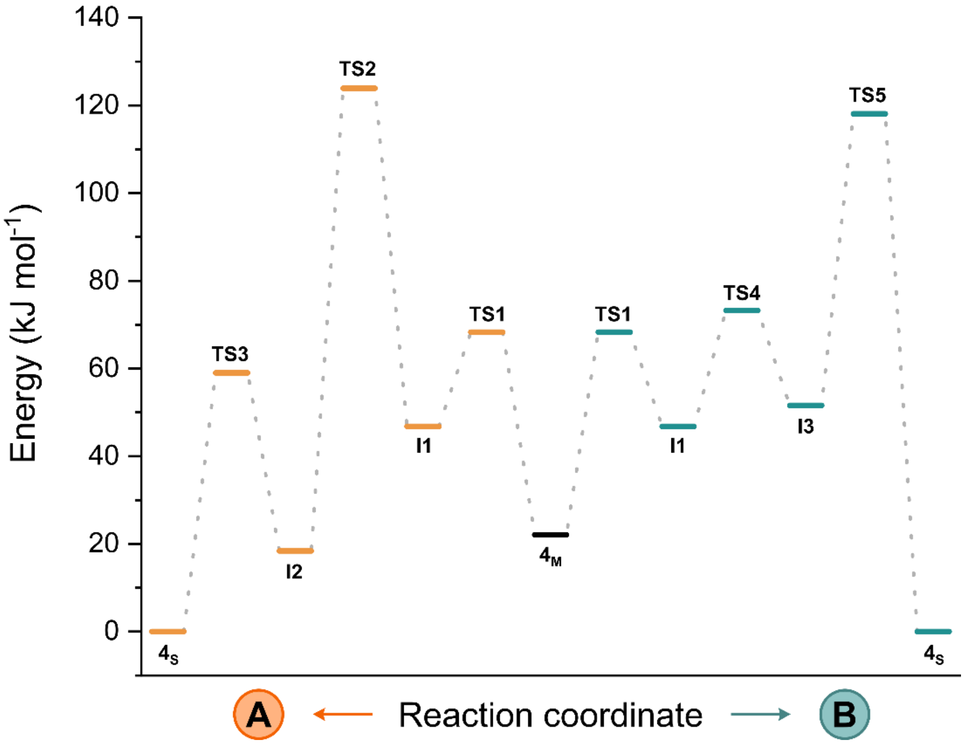


**Figure S31. Computational study of thermal isomerization pathway of motor 4.** Energy profile along the reaction coordinate for the thermal relaxation from metastable $\mathbf{4_M}$ to stable $\mathbf{4_S}$.

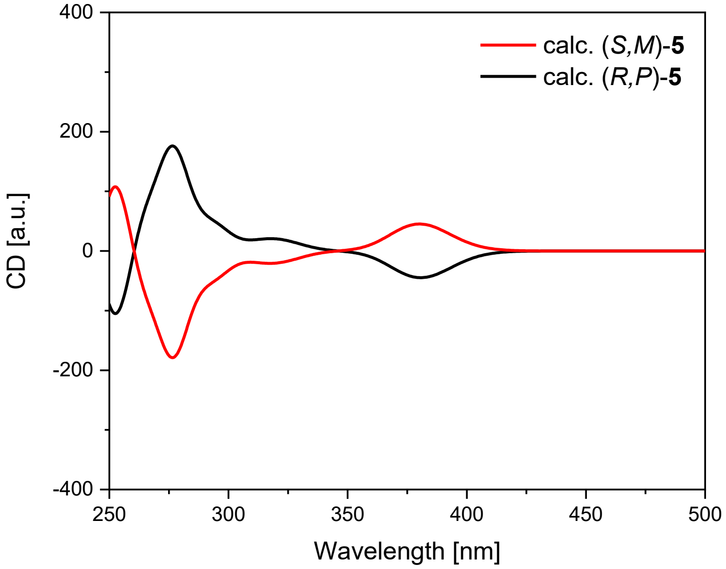


**Figure S32. Theoretical ECD spectra of the enantiomers of motor 5.** The calculated spectra with TD-DFT were shifted by 30 nm (with TDA, applying Gaussian shapes (line width = 0.3 eV) to 30 discrete transitions).

In the second part of the computational investigation the focus was set on providing rationale for the improved performance of the formylated motors on the grounds of the in-depth excited-state properties analysis. To this end, three methodological workflows were followed.

Firstly, the TD-DFT UV-Vis absorption calculations were performed with the Gaussian 16 software[101] at the CAM-B3LYP/def2-TZVPP level of theory,[102] including TDA and BJ-D3[103] dispersion correction, and using the CPCM model with parameters for the $CH_2Cl_2$ as solvent. In this part, ground-state molecular structures optimized with the r$^2$SCAN-3c methods were employed. Comparison of the results obtained for the formylated motors and their unsubstituted parent systems revealed lowering of the HOMO and LUMO energies of the formylated derivatives, with the LUMO stabilization being systematically larger in all compounds thanks to the more extended system of the conjugated $\pi$ bonds. Eventually, the energy of the $S_0 \rightarrow S_1$ transition, dominated by the HOMO $\rightarrow$ LUMO excitation, goes down leading to the observed redshift of the lowest band in the experimental UV-Vis absorption spectra.

Secondly, a TD-DFT CAM-B3LYP/def2-SVP[97] level of theory including TDA and the BJ-D3 dispersion correction was used to perform unrestricted structural optimizations, and to obtain adiabatic potential energy profiles along the rotor rotation dihedral angle for Motor 2 and its unsubstituted analog, in the lowest excited singlet state ($S_1$). This part of calculations has been also run with the Gaussian 16 software package.

Finally, we employed a multi-reference semi-empirical electronic structure method, ODM2/MRCI,[104] to perform 2D adiabatic potential energy surface scans in the $S_0$ and in the $S_1$ state for Motor 2 and its unsubstituted parent system. These calculations were followed by unrestricted structural optimizations of the $S_0$ and $S_1$ minima and of the minimum-energy $S_1/S_0$ conical intersection points. The orbital active space in multi-reference calculations contained 12 electrons distributed in 12 orbitals, with the molecular orbitals optimized for the lowest-energy open-shell configuration. Three electronic-configuration determinants were employed in the MRCI procedure which included single and double excitations (CISD): the one used for the orbital optimization, and the two closed-shell configurations that can be built therefrom. The semi-empirical calculations were performed with the MNDO2020 code.[105]

The excited-state TD-DFT and semi-empirical calculations performed for Motor 2 and its parent molecule reveal close resemblance of the two. In both systems the $S_1$ state has a $\pi\pi^*$ nature and characterizes with an extended flat potential energy surface between the Franck-Condon regions of the respective stable (S) and metastable (M) forms and the conical intersection (CI) region. TD-DFT predicts three very shallow local excited-state minima: two for the formylated system (S*-min and M*-min) and one for the unsubstituted motor molecule (S*-min), with the remaining M* optimization of the latter leading directly to the CI vicinity. At the same time, the semi-empirical calculations yielded only one $S_1$ minimum per system, positioned in both molecules close to the CI area. Energy-wise, both theoretical methods predict the unsubstituted motor to possess a slightly higher kinetic energy excess at the CI region. Moreover, the 2D potential energy scans show a slightly wider low-$S_1/S_0$-gap area for the unsubstituted motor which could altogether open a way for a more efficient through-seam nonradiative relaxation in this system, as compared to the formylated derivative. The optimized minimum-energy conical intersection points, determined at the ODM2/MRCI level, lie about 100-150 meV (MECI-1) and about 400 meV (MECI-2) above the central $S_1$ minima, in all cases remaining energetically accessible to the systems after their excitation to the lowest absorbing state at respective Franck-Condon regions.

Eventually, given the overall strong resemblance of the studied bare and formylated 2$^{nd}$ generation motors, we predict the observed favorable properties of the latter to be likely due to a combination of more subtle electronic-structure effects, which might require a higher-level and more extensive theoretical investigation to be unequivocally characterized.

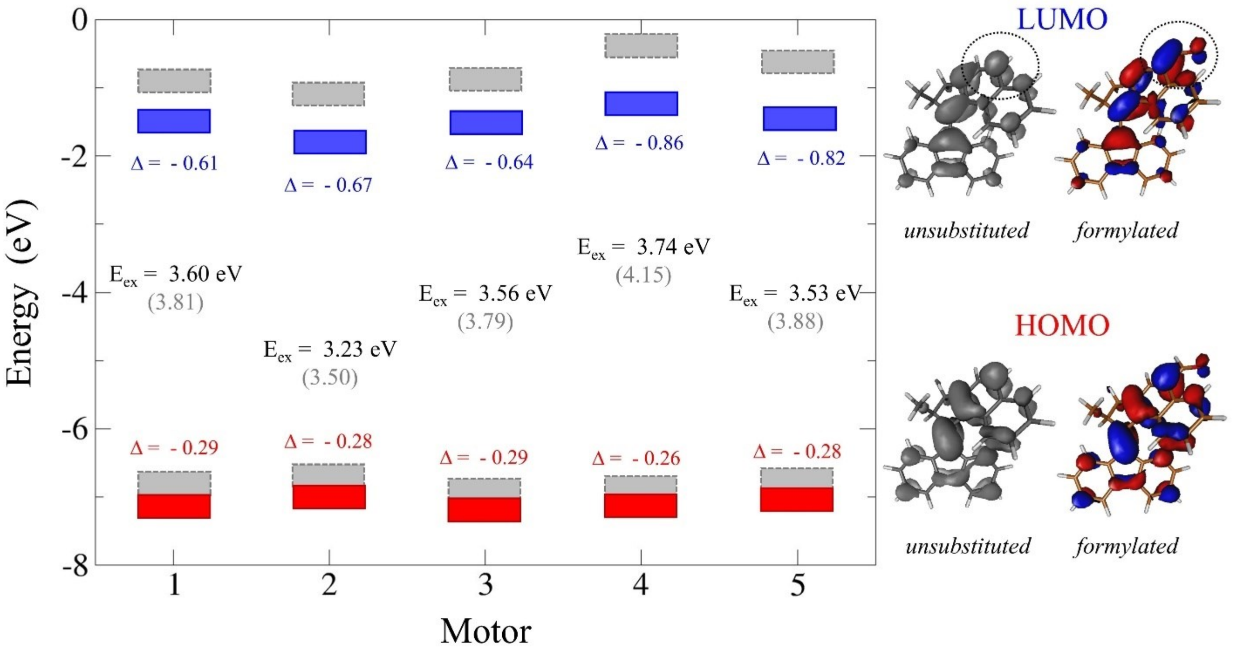


**Figure S33. Frontier orbitals of stable isomers of formylated motors.** HOMO (red) and LUMO (blue) orbital energies and the $S_0 \rightarrow S_1$ absorption energies (black) determined at the TDA TD-DFT CAM-B3LYP/def2-TZVPP level of theory, with the BJ-D3 dispersion correction, and with the $CH_2Cl_2$ solvent included with the CPCM model, calculated at stable (S) molecular structures of all studied formylated motors optimized with the $r^2$SCAN-3c method. All corresponding data for the unsubstituted precursors are shown in gray. The red/blue Δ values mark the orbital energy change due to formylation.

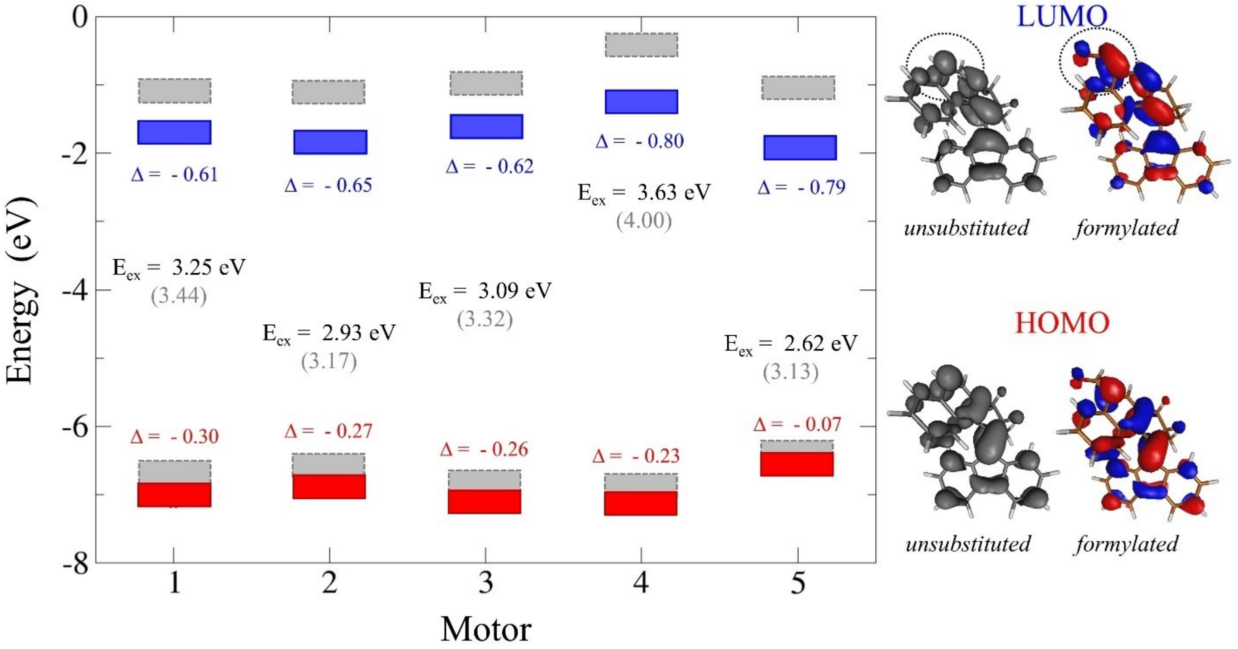


**Figure S34. Frontier orbitals of metastable isomers formylated motors.** HOMO (red) and LUMO (blue) orbital energies and the $S_0 \rightarrow S_1$ absorption energies (black) determined at the TDA TD-DFT CAM-B3LYP/def2-TZVPP level of theory, with the BJ-D3 dispersion correction, and with the $CH_2Cl_2$ solvent included with the CPCM model, calculated at metastable (M) molecular structures of all studied formylated motors optimized with the $r^2$SCAN-3c method. All corresponding data for the unsubstituted precursors are shown in gray. The red/blue Δ values mark the orbital energy change due to formylation.

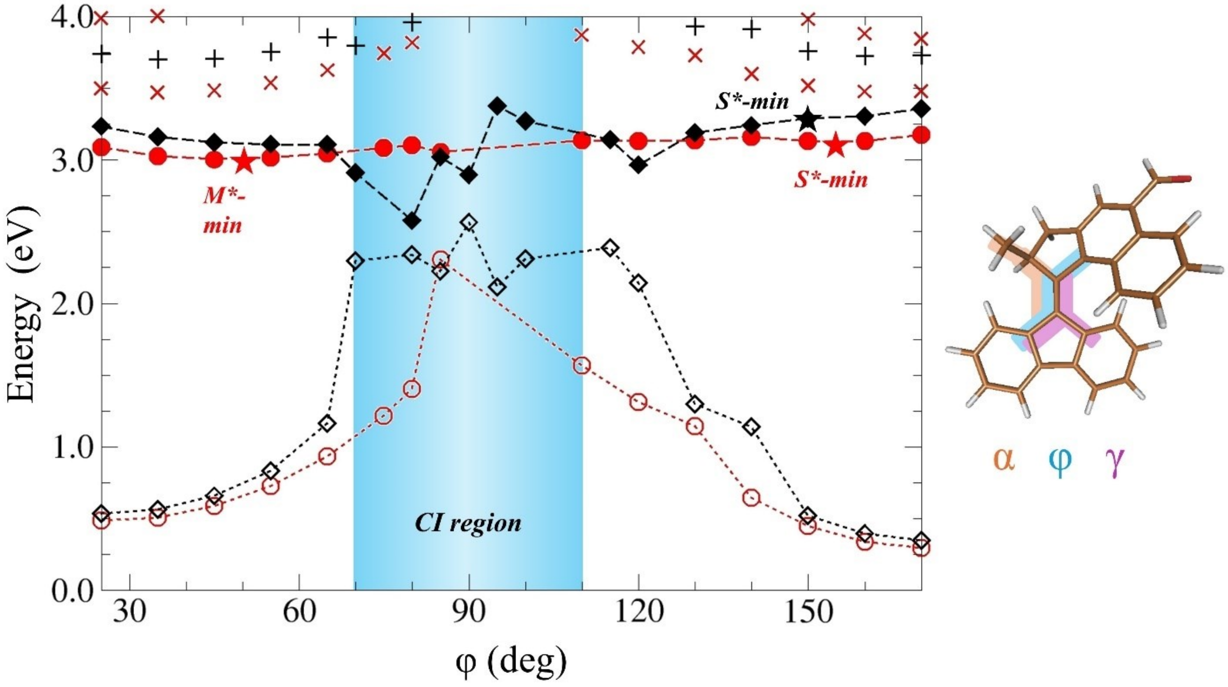


**Figure S35. Adiabatic potential energy profiles**. The profiles were optimized for the $S_1$ state, determined for motor **2** and its unsubstituted parent molecule at the TDA TD-DFT CAM-B3LYP/def2-SVP level of theory with the BJ-D3 dispersion correction. Red/black symbols mark data corresponding to the formylated/unformylated system, respectively. Empty symbols with dotted lines show vertical energies of the $S_0$ state, full symbols with dashed lines mark adiabatic energies of the $S_1$ state, and 'x' and cross symbols depict vertical energies of the higher excited states below 4.0 eV (up to $S_3$). All energies calculated referred to the respective S form energy, as determined at the CAM-B3LYP/def2-SVP level of theory at the optimized molecular structure. Full stars show positions of the excited-state minima determined at the TD-DFT level. On the right, in the inset: definition of dihedral angles used for analysis of the excited-state theoretical calculations. α – axial/equatorial conformation of the methyl group; φ – rotor rotation about the central CC bond; γ – pyramidization of the central stator carbon atom.

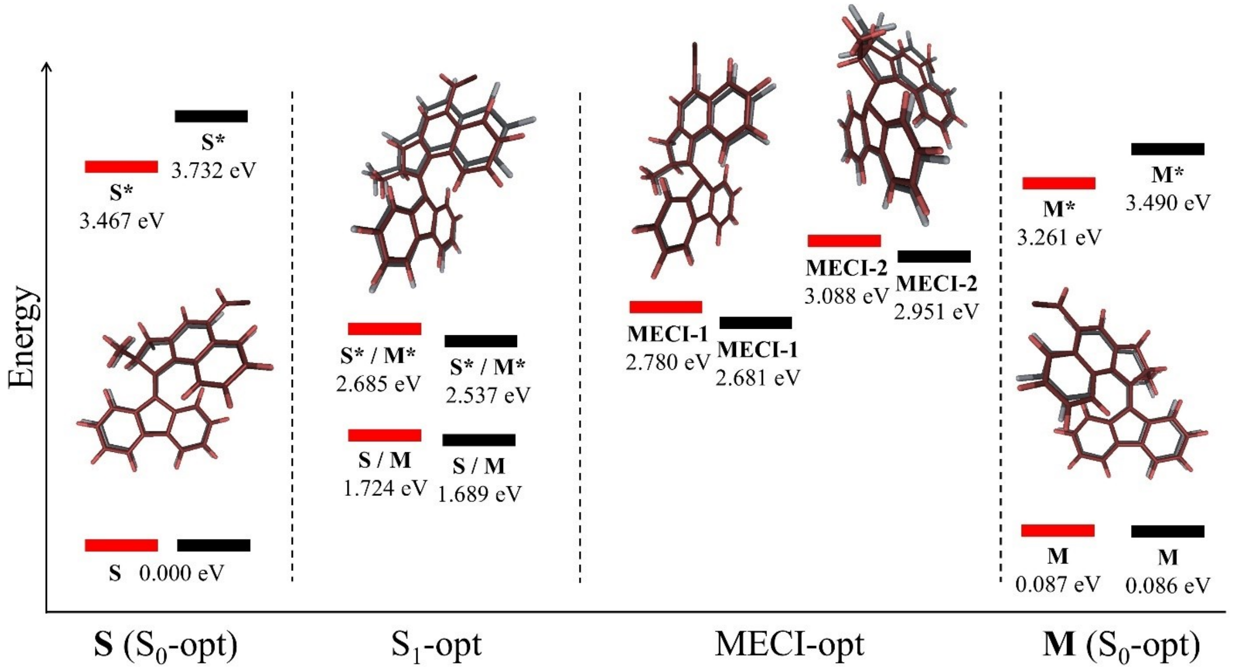


**Figure S36. Energy profile of photochemical isomerization.** Excited- (S*, M*) and ground-state (S, M) energies at stationary geometries determined for motor **2** (red) and its unsubstituted analog (black) at the ODM2/MRCI level of theory. In the inset, overlapped optimized molecular structures.

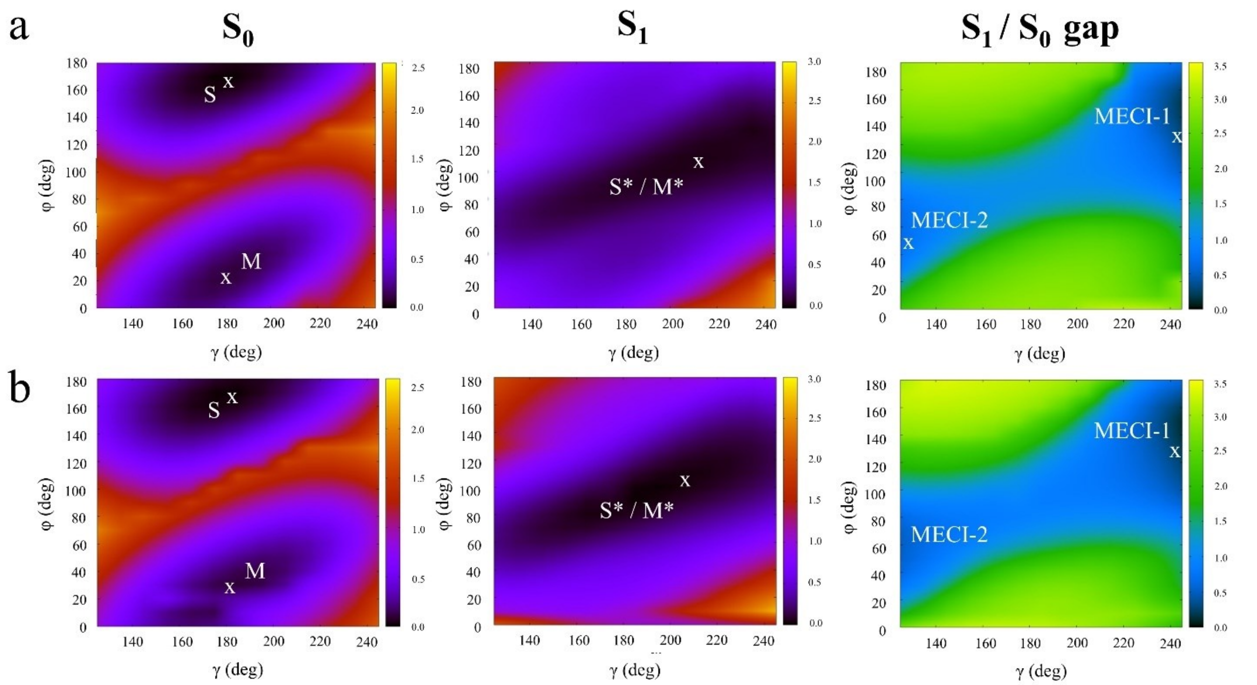


**Figure S37. Adiabatic 2D potential energy scans**. Scans were performed along rotor rotation (φ) and central stator carbon atom pyramidization (γ) internal coordinates, determined at the ODM2/MRCI level of theory for motor **2** (a) and its unsubstituted parent system (b). On the left: ground-state energies obtained in the relaxed $S_0$ scan; in the center: lowest excited-state energies obtained in the relaxed $S_1$ scan; on the right: $S_1/S_0$ energy gap determined along the $S_1$-optimized potential energy surface scan. The 'x' marks show positions of respective stationary structures.

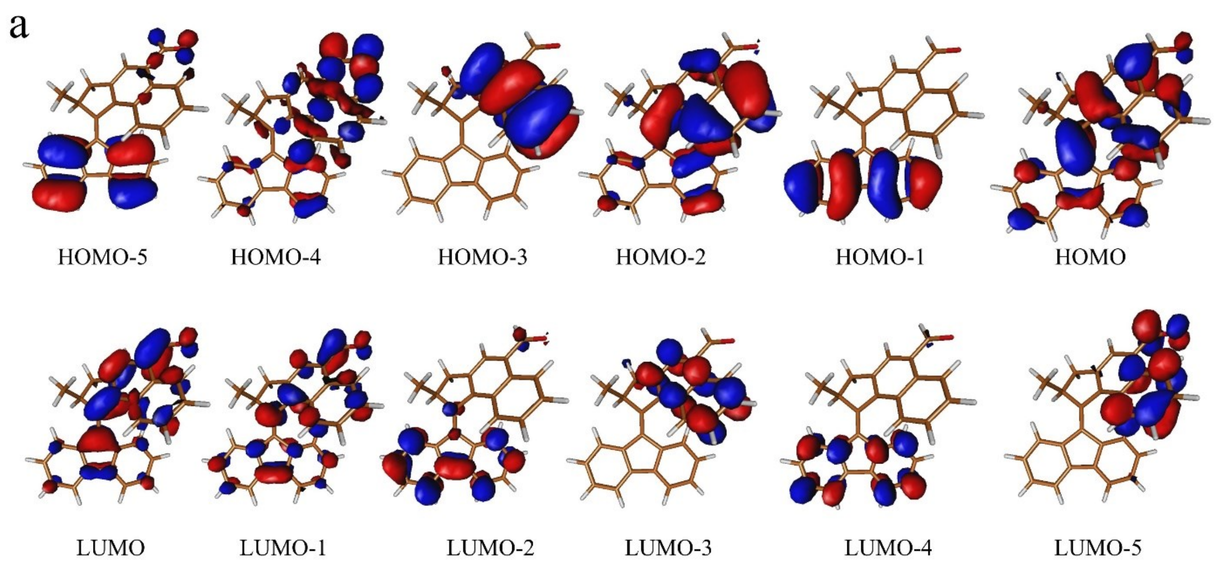


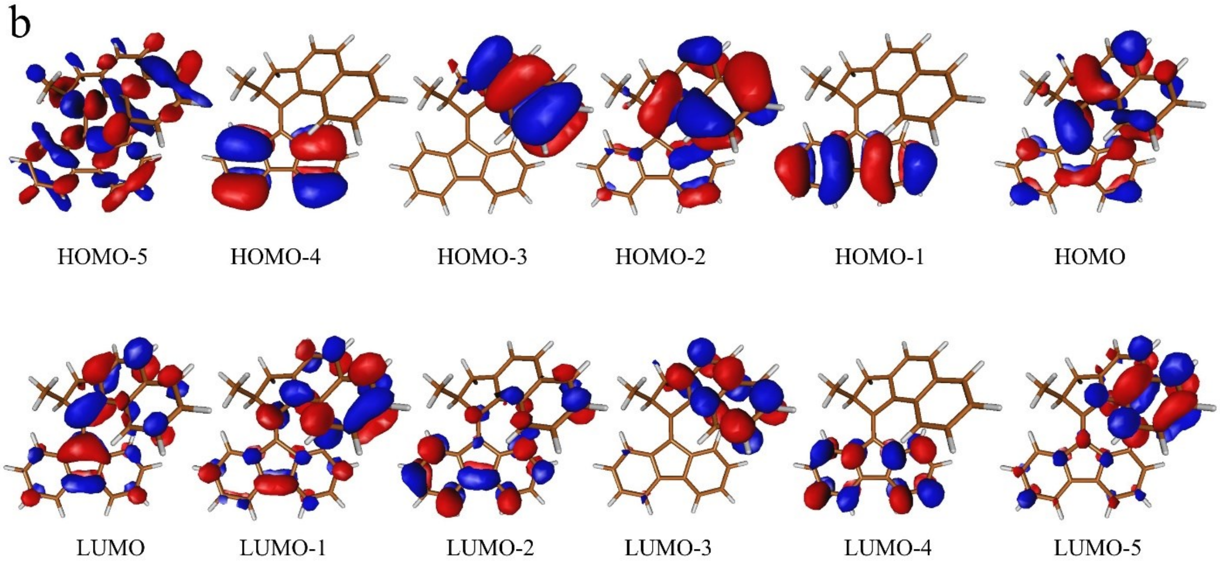


**Figure S38. Molecular orbital active spaces.** Depiction of the active spaces employed in the ODM2/MRCI calculations for motor **2** (a), and its unsubstituted analog (b).

NMR spectra of fresh and aged motor samples

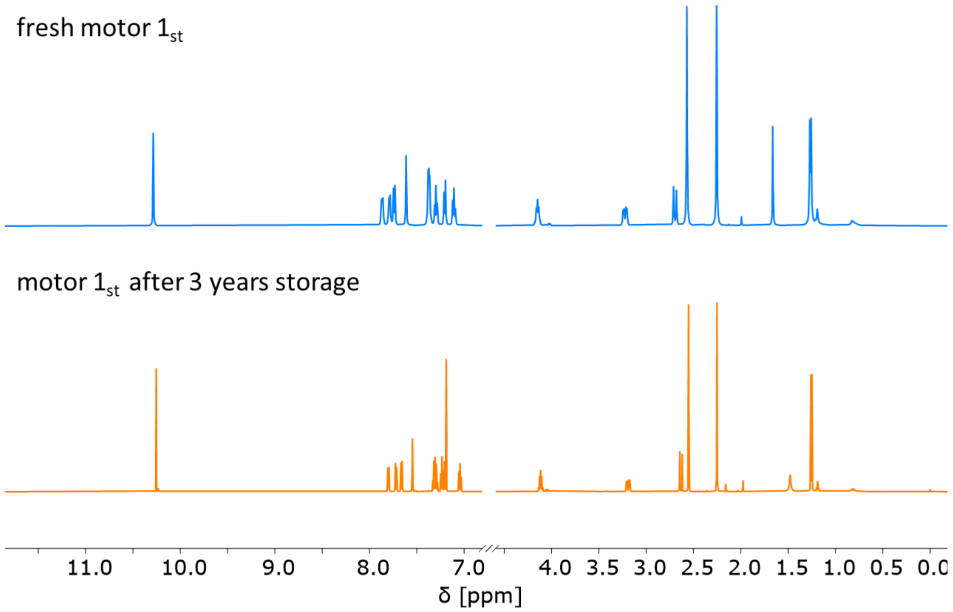


**Figure S39. Stability of motor 1.** Comparison of $^{1}H$ NMR spectra of fresh prepared motor **1st** (room temperature, $CD_2Cl_2$) and the sample after 3 years storage under ambient condition (room temperature, $CDCl_3$).

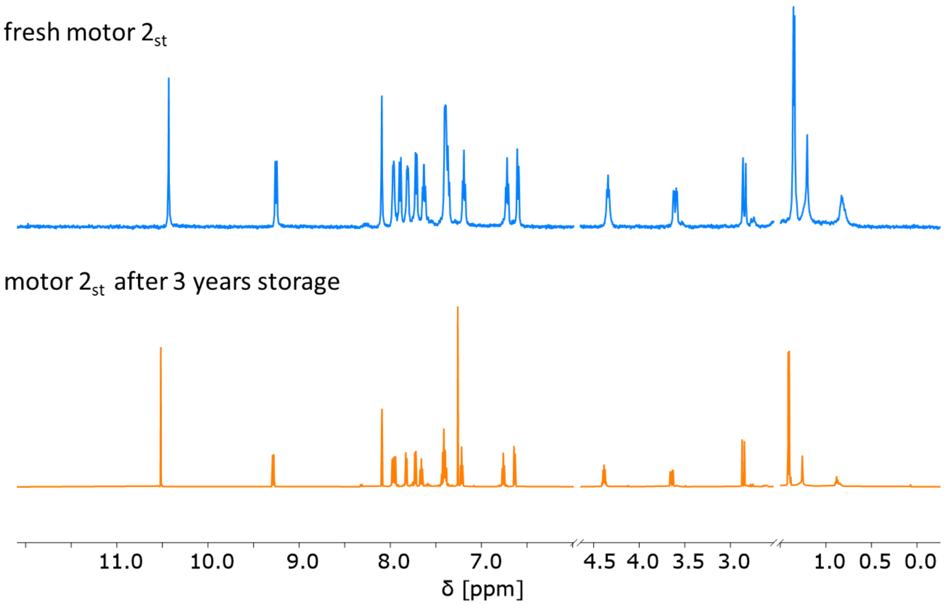


**Figure S40. Stability of motor 2.**Comparison of $^{1}H$ NMR spectra of fresh prepared motor **2st** (room temperature, $CD_2Cl_2$) and the sample after 3 years storage under ambient condition (room temperature, $CDCl_3$).

<u>HRMS spectra</u>

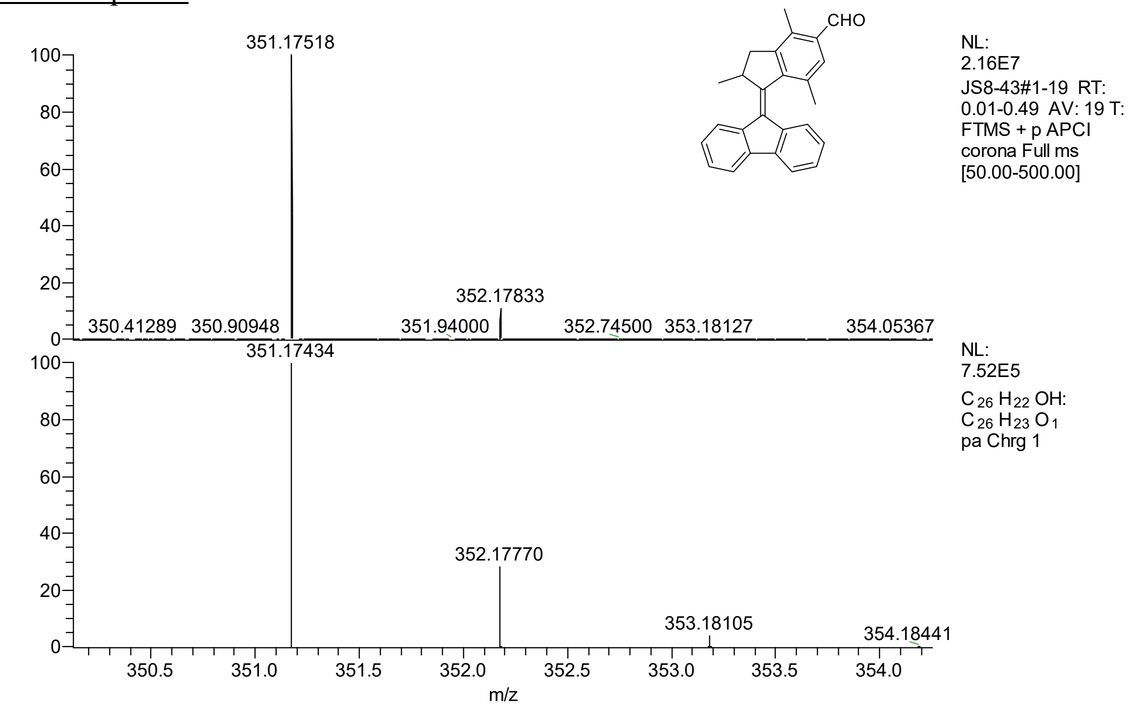


HRMS (APCI) spectra of **1st** (top:measured, bottom: calcd.).

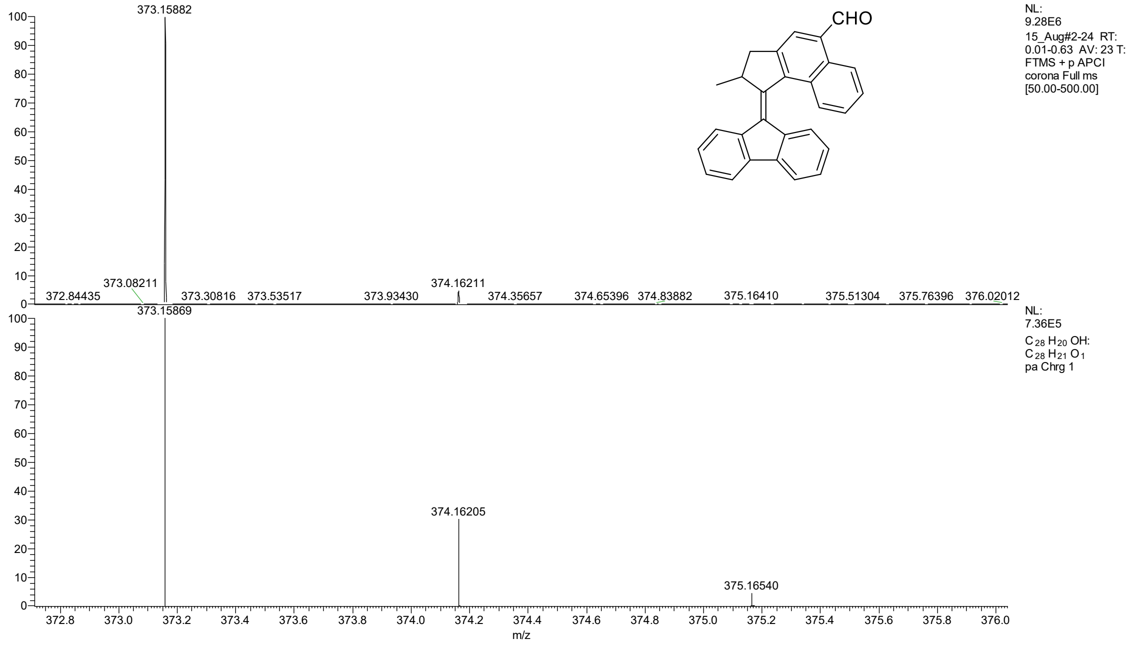


HRMS (APCI) spectra of **2st** (top:measured, bottom: calcd.).

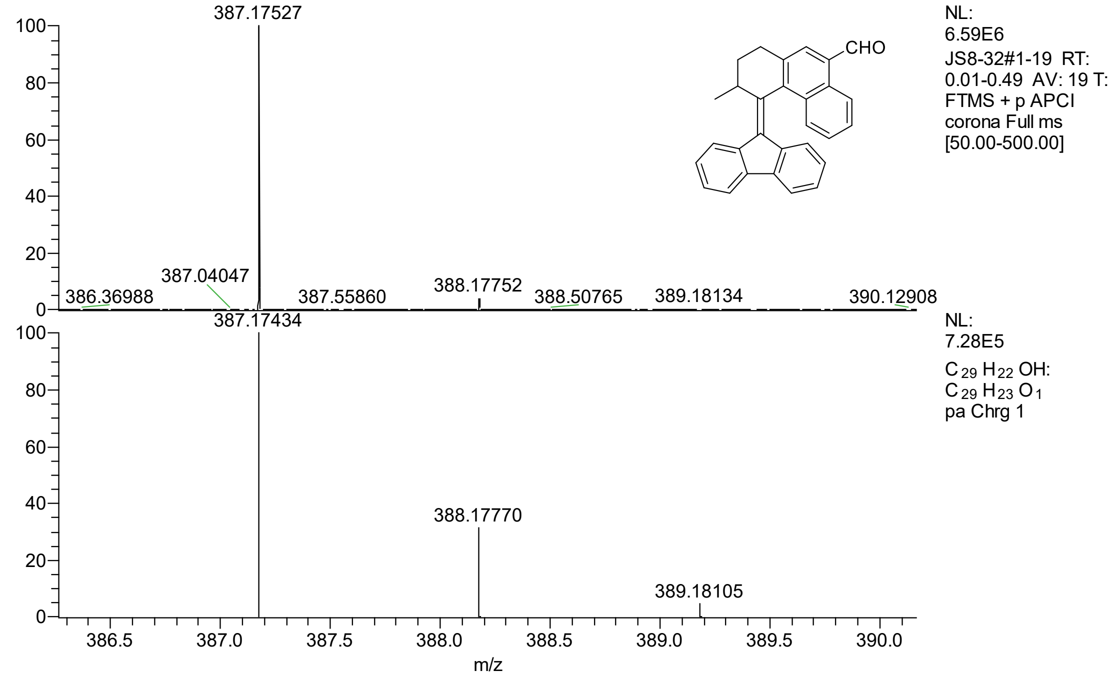


HRMS (APCI) spectra of **3st** (top:measured, bottom: calcd.).

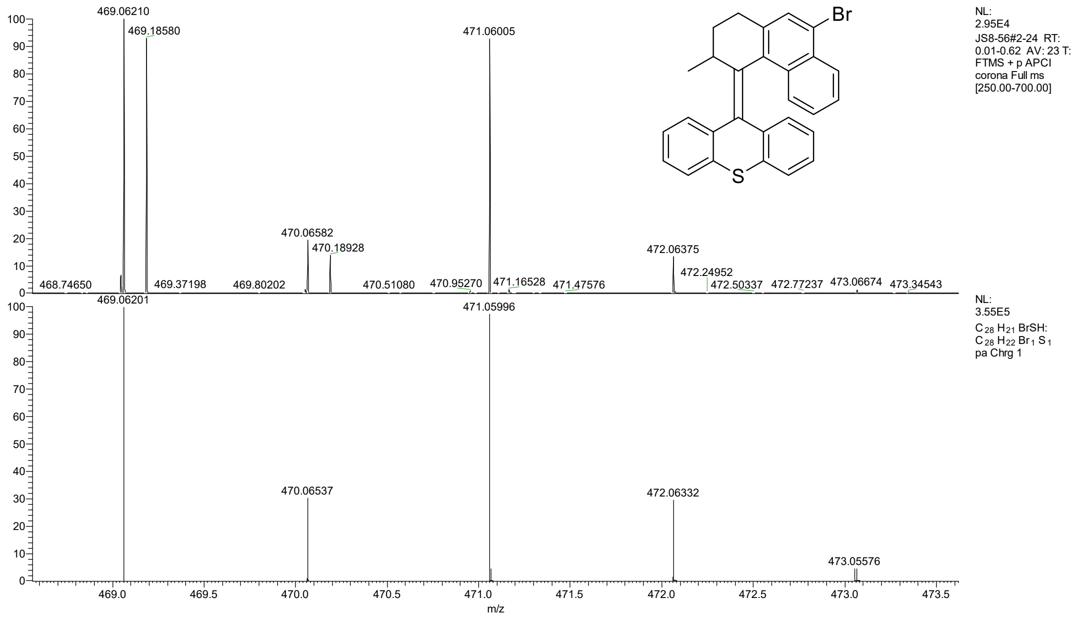


HRMS (APCI) spectra of **4’st** (top:measured, bottom: calcd.).

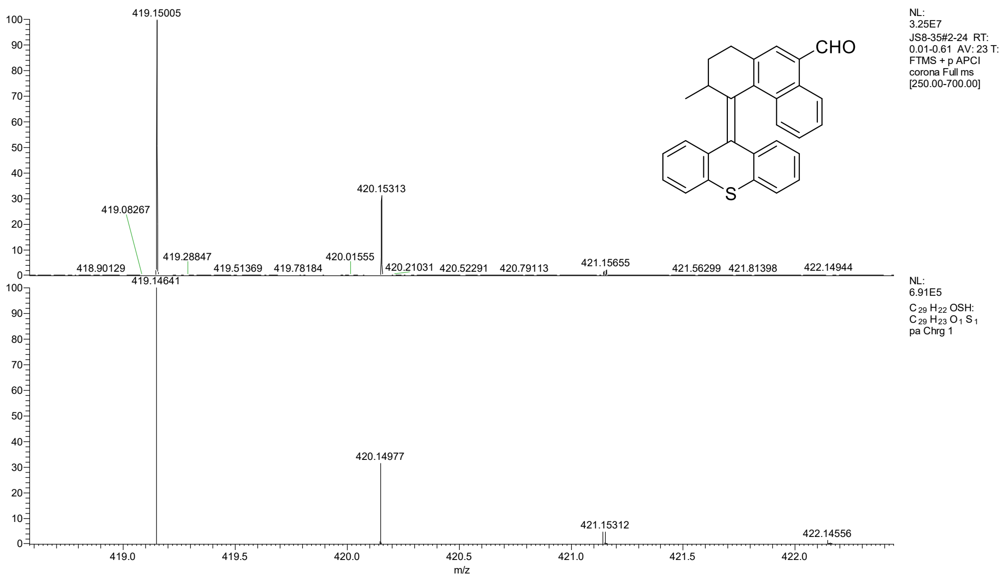


HRMS (APCI) spectra of **4st** (top:measured, bottom: calcd.).

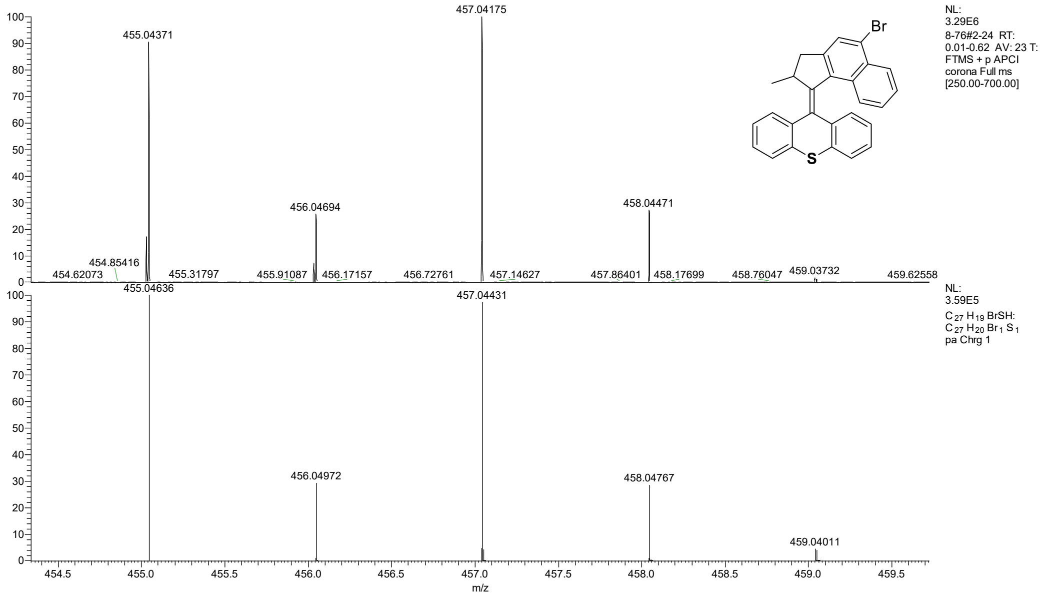


HRMS (APCI) spectra of **5'st** (top:measured, bottom: calcd.).

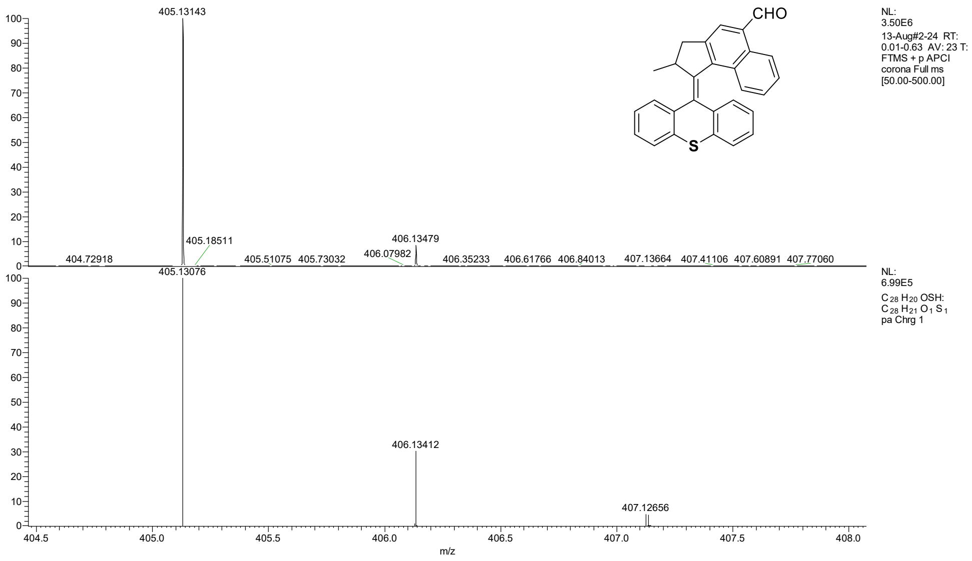


HRMS (APCI) spectra of **5st** (top:measured, bottom: calcd.).

# NMR data of compounds

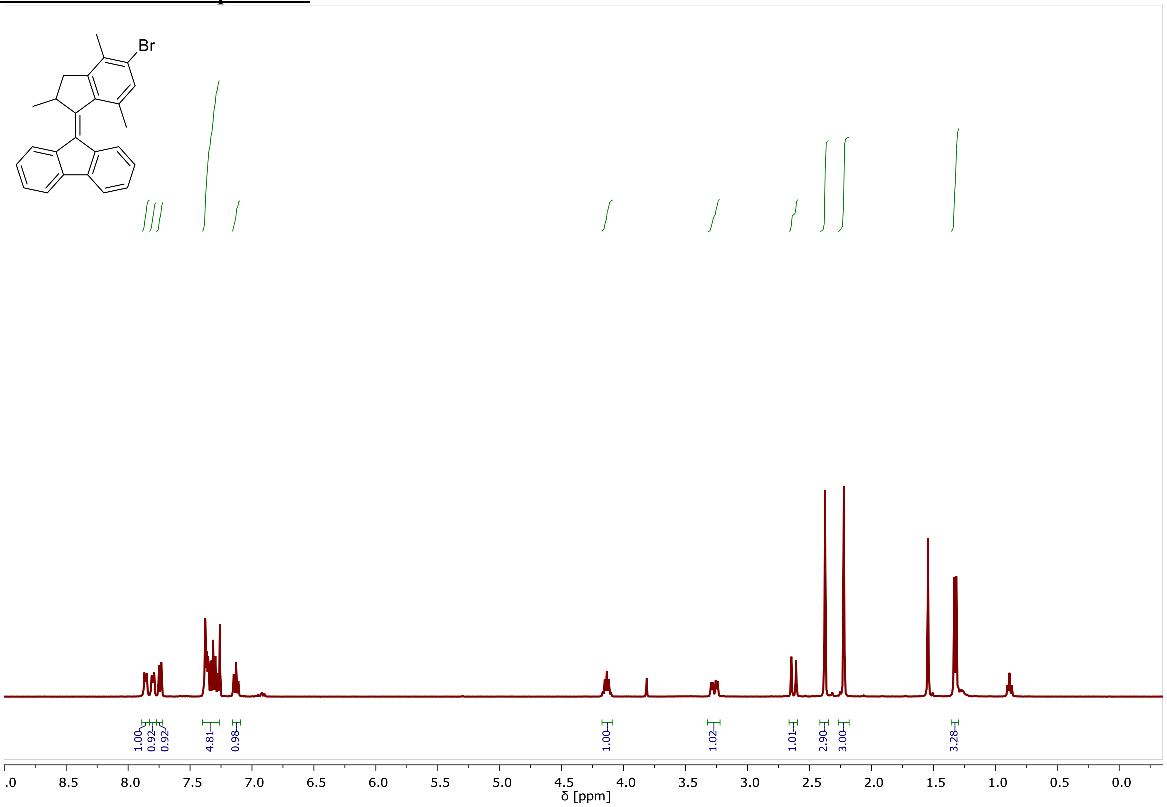


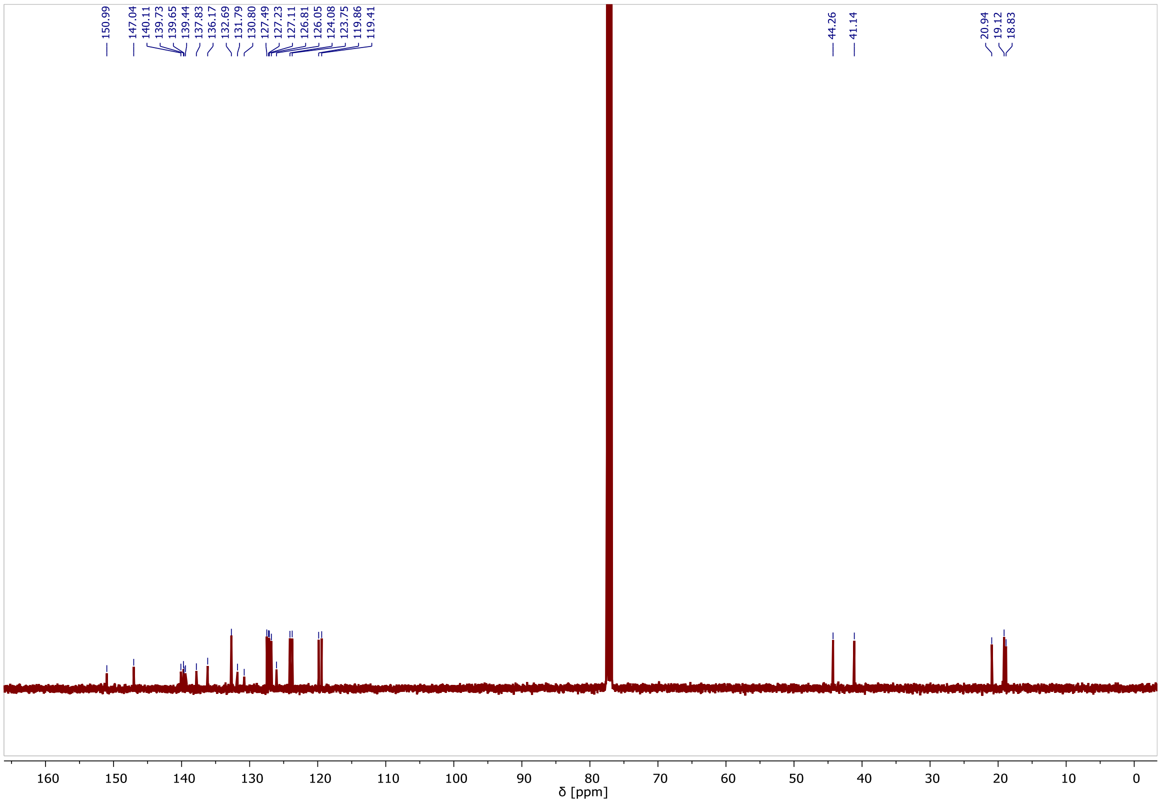


$^{1}H$ and $^{13}C$ NMR of compound **1’.**

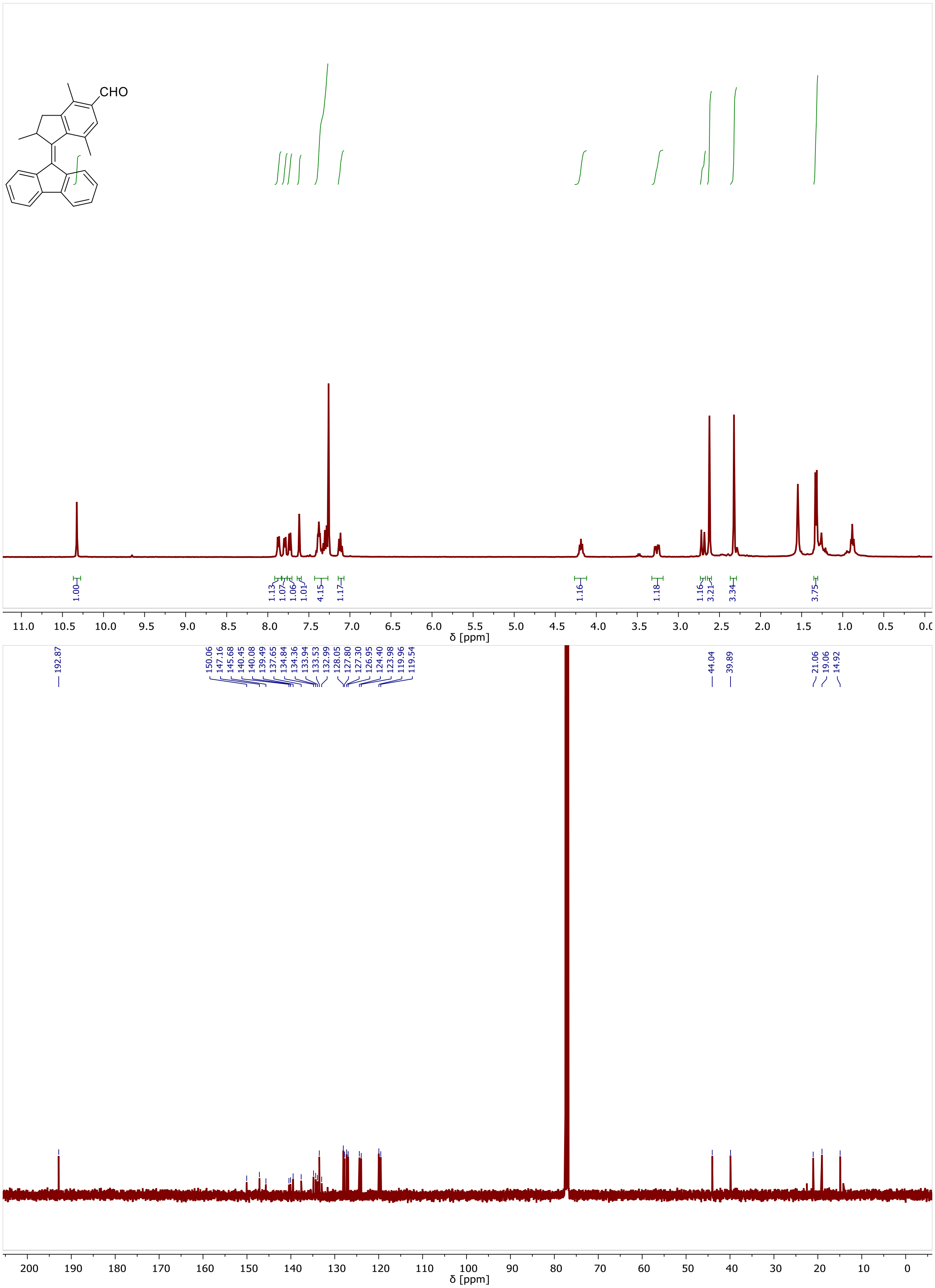


$^{1}$H and $^{13}$C NMR of compound **1.**

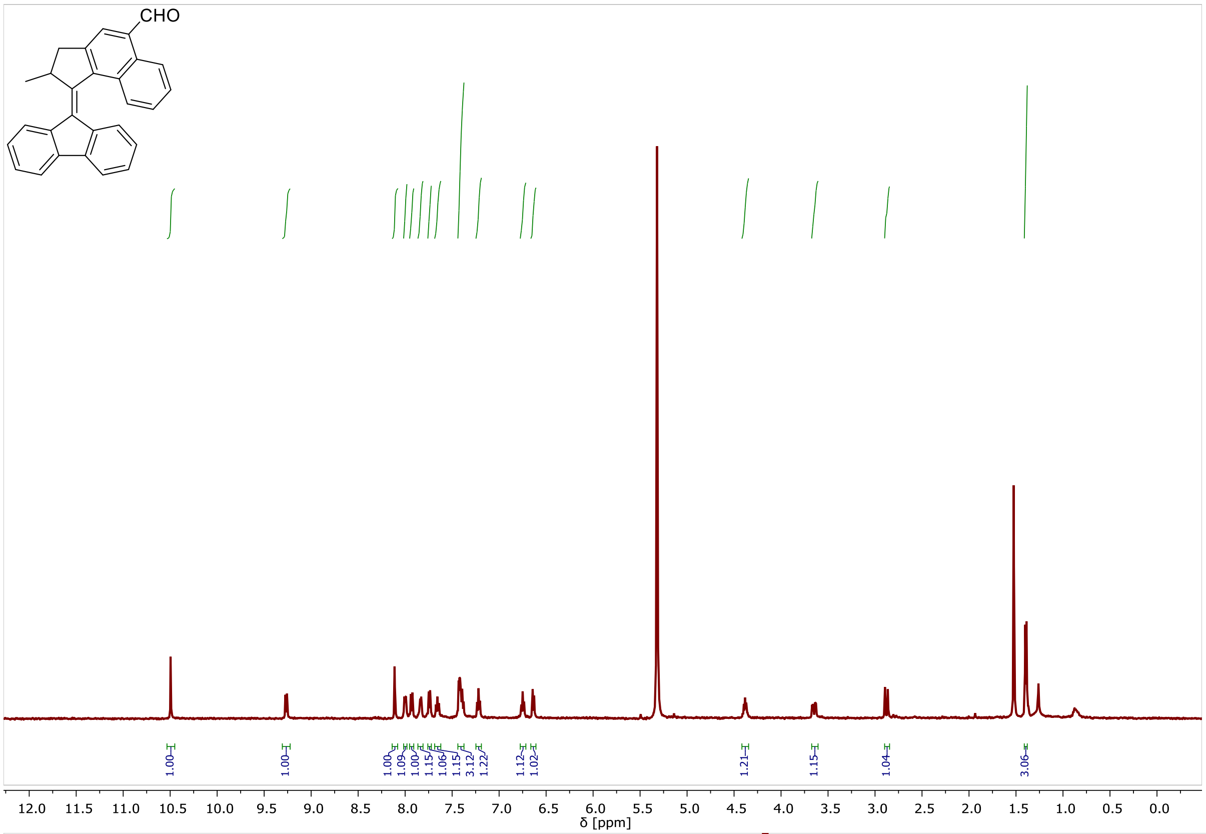


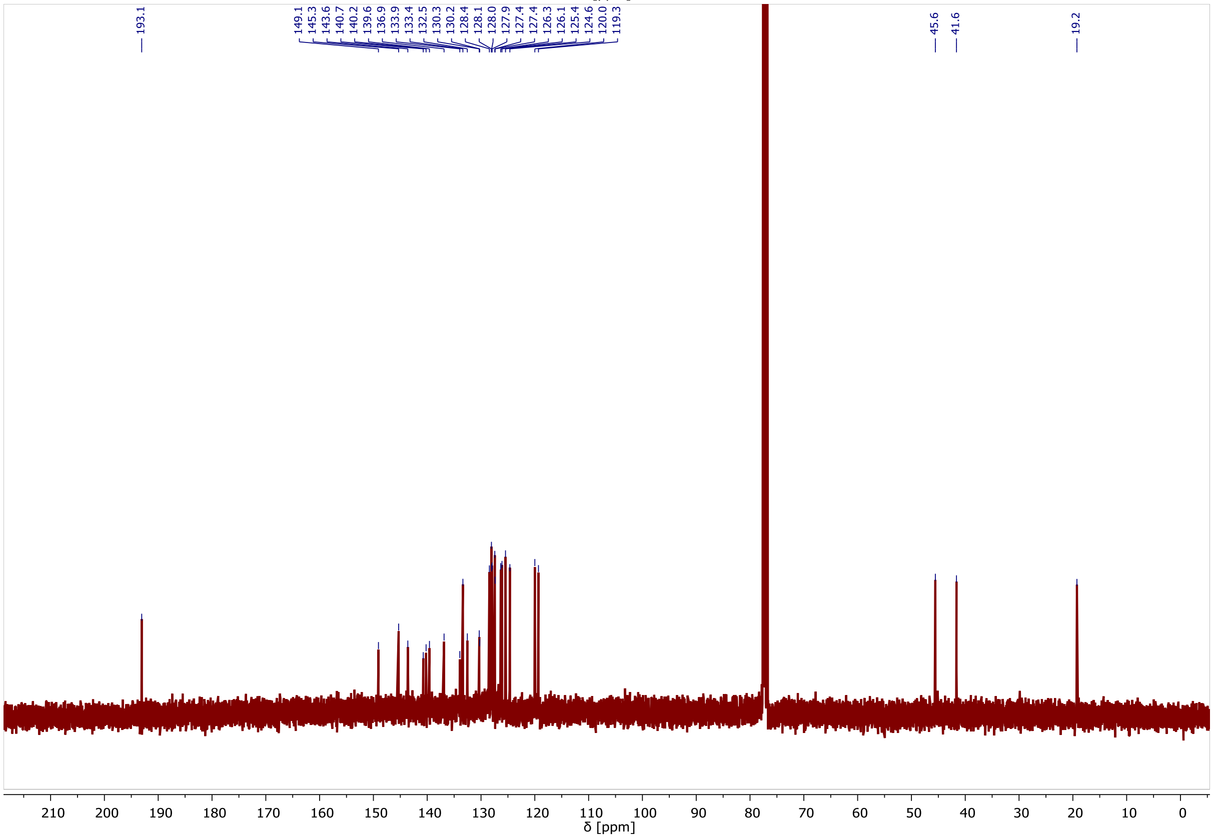


$^{1}$H and $^{13}$C NMR of compound **2.**

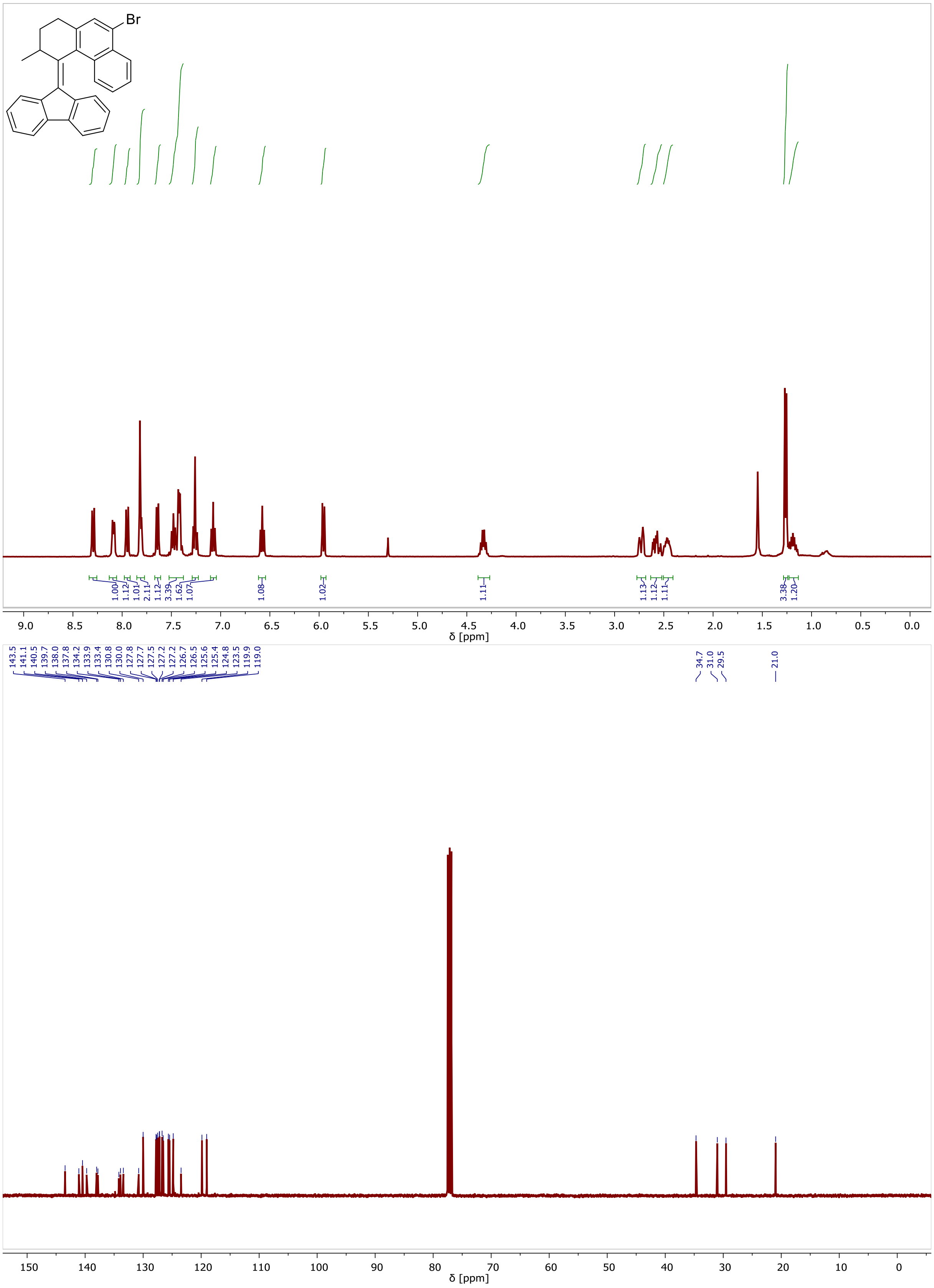

$^{1}$H and $^{13}$C NMR of compound **3’.**

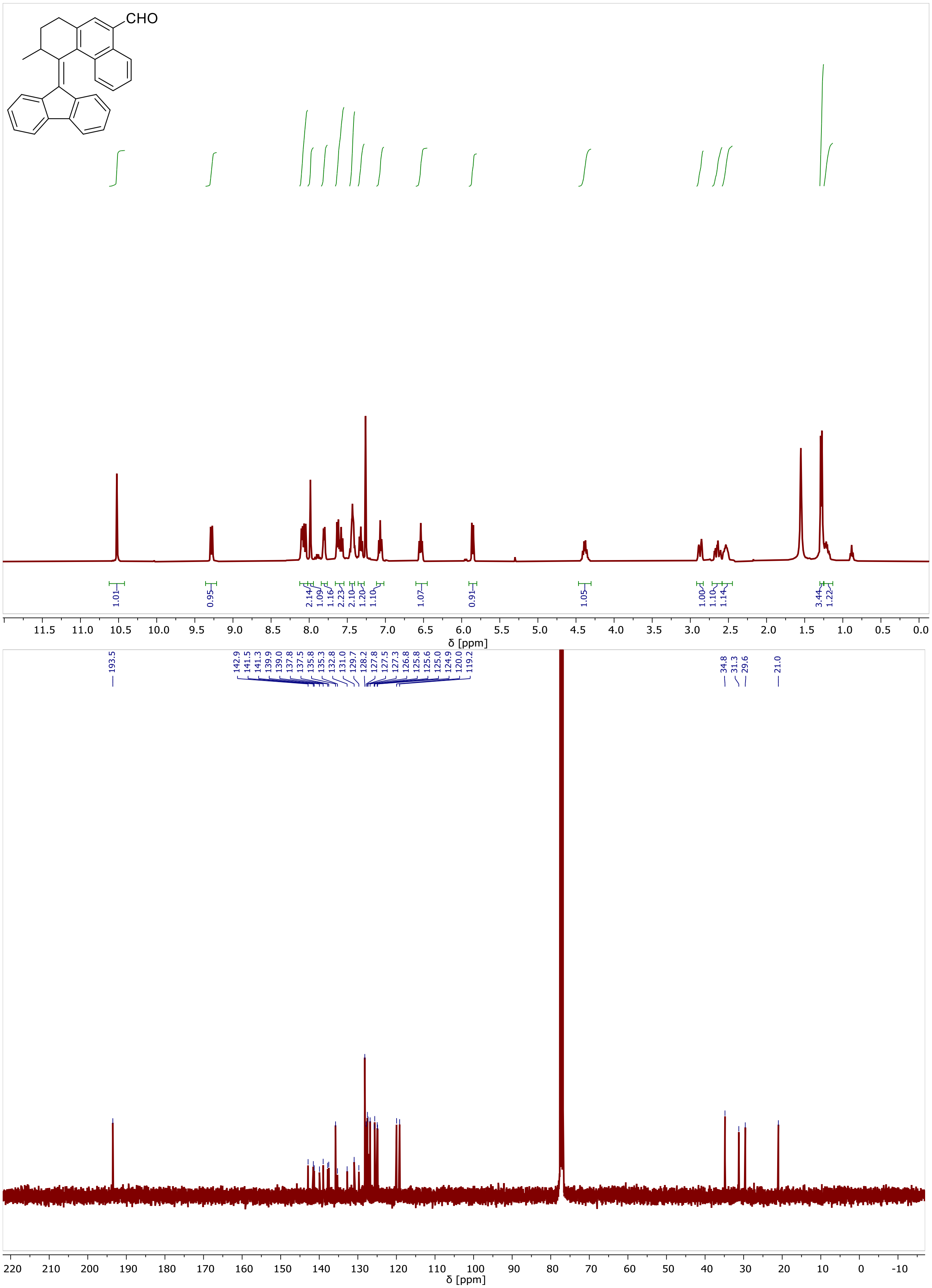


$^1$H and $^{13}$C NMR of compound **3.**

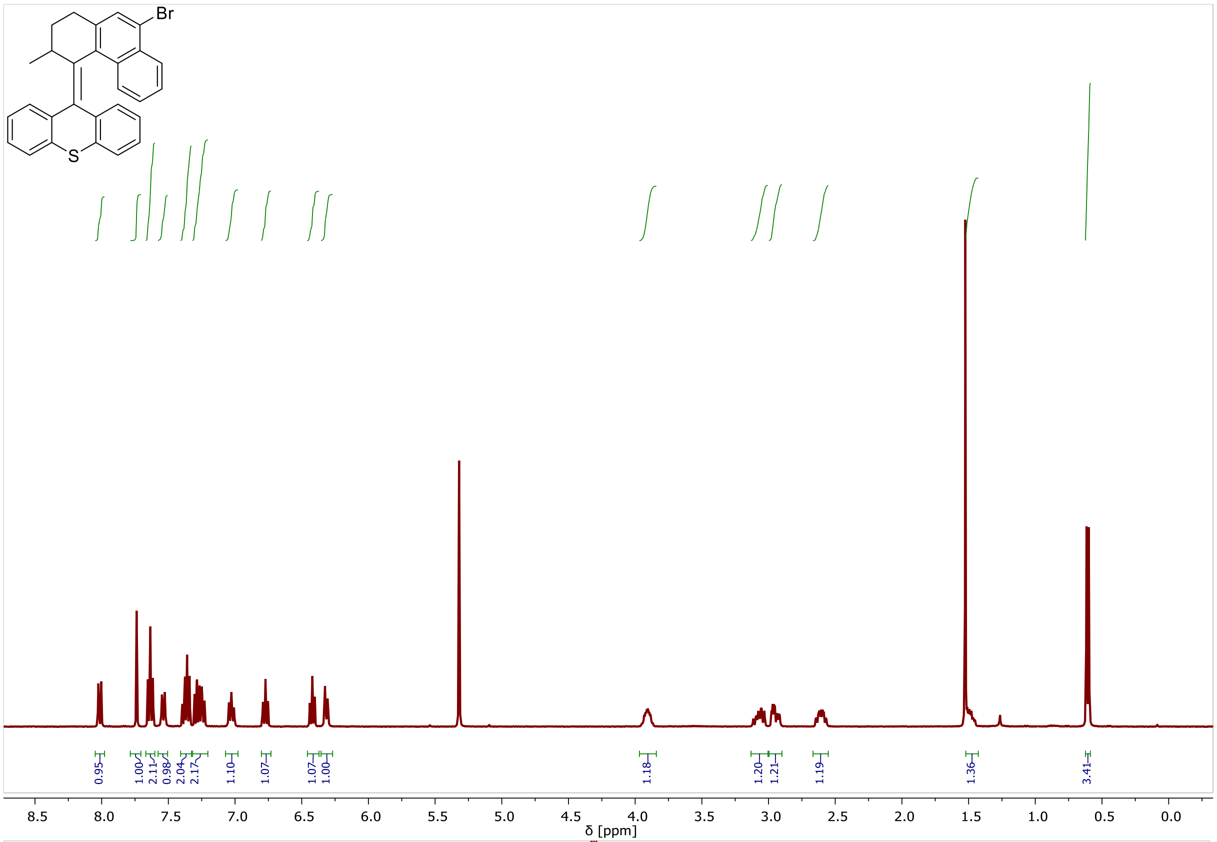

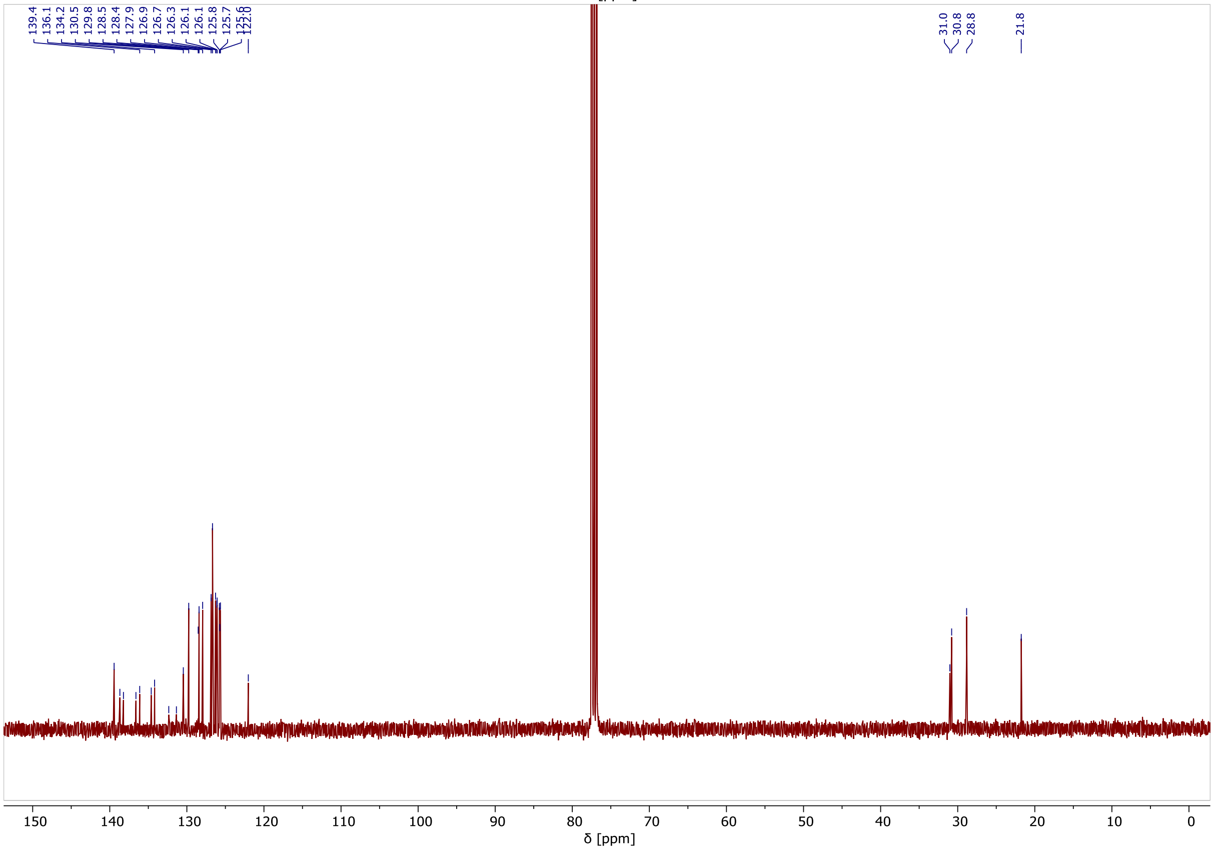

$^{1}$H and $^{13}$C NMR of compound **4’.**

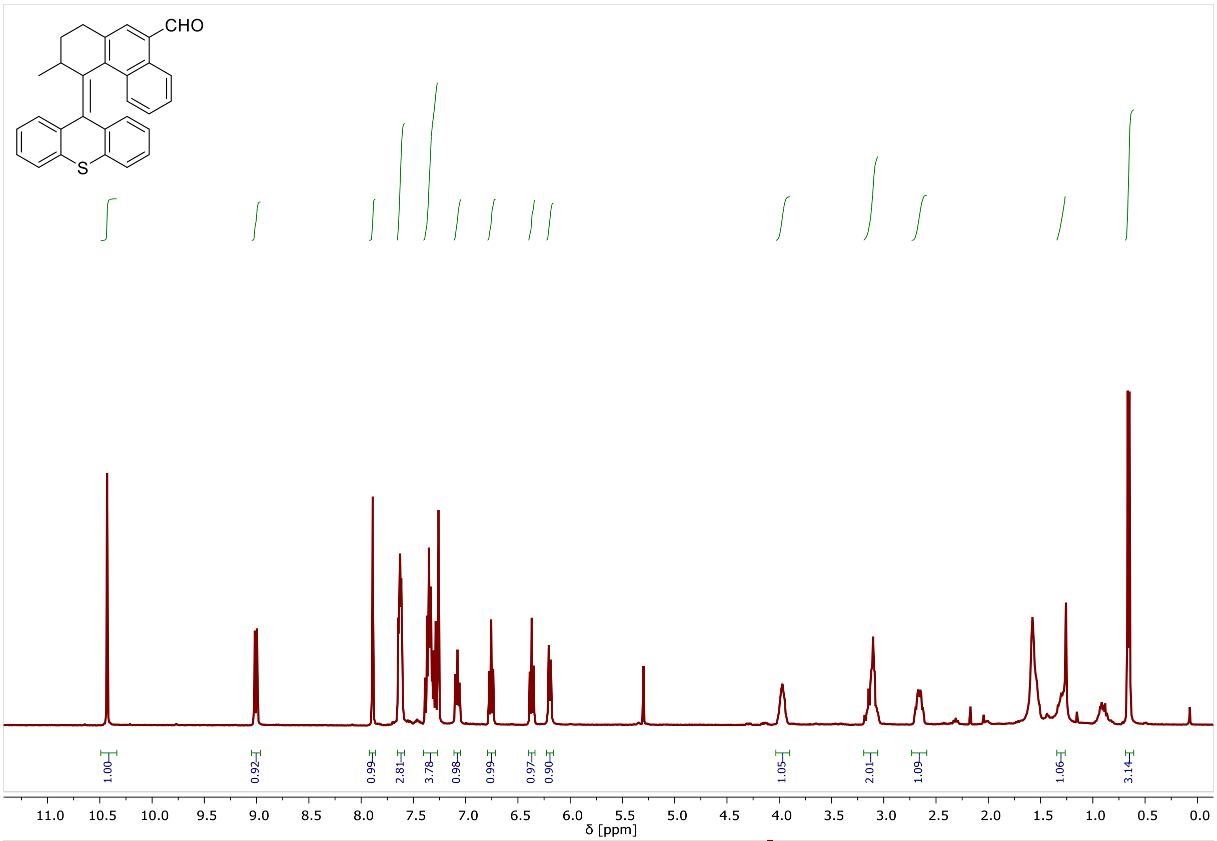


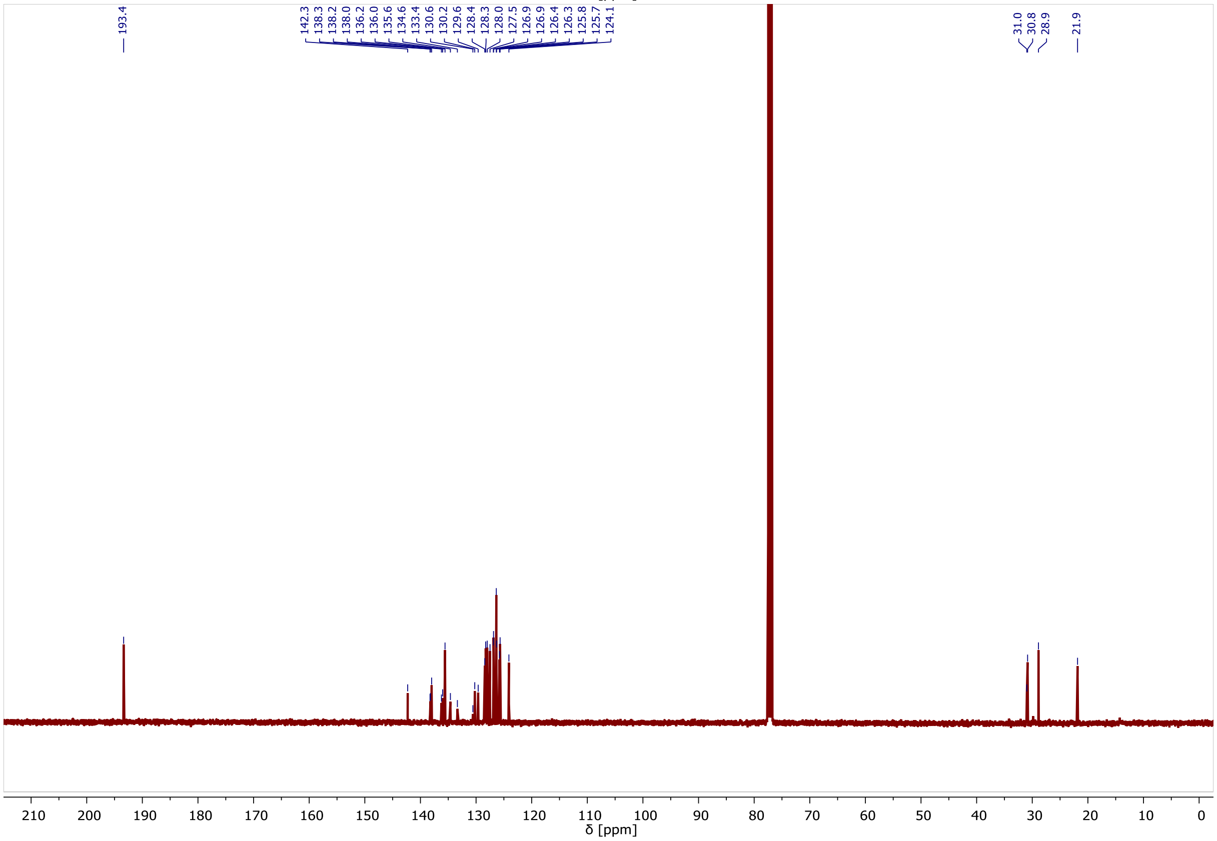


$^1$H and $^{13}$C NMR of compound **4.**

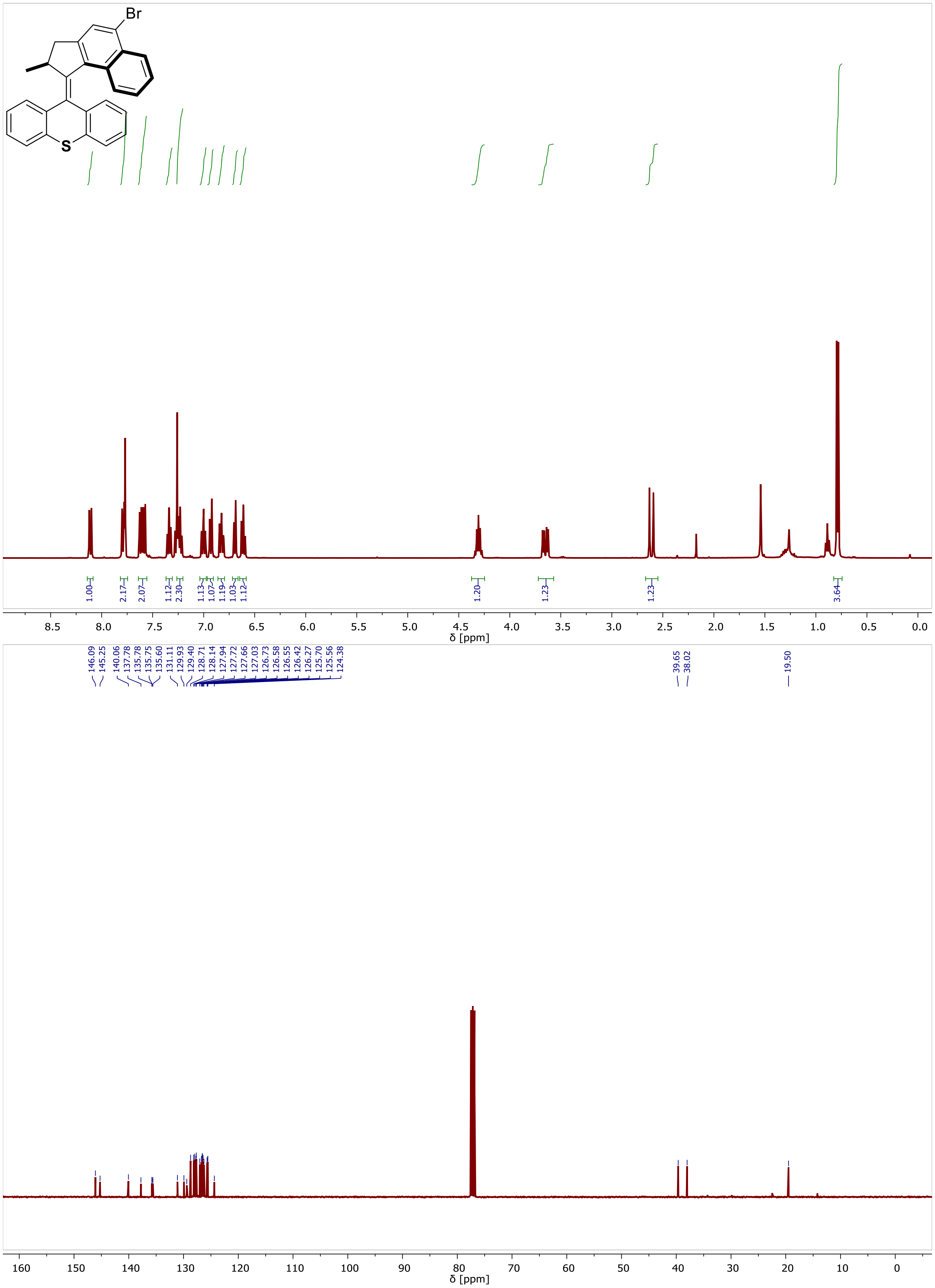


$^{1}H$ and $^{13}C$ NMR of compound **5’.**

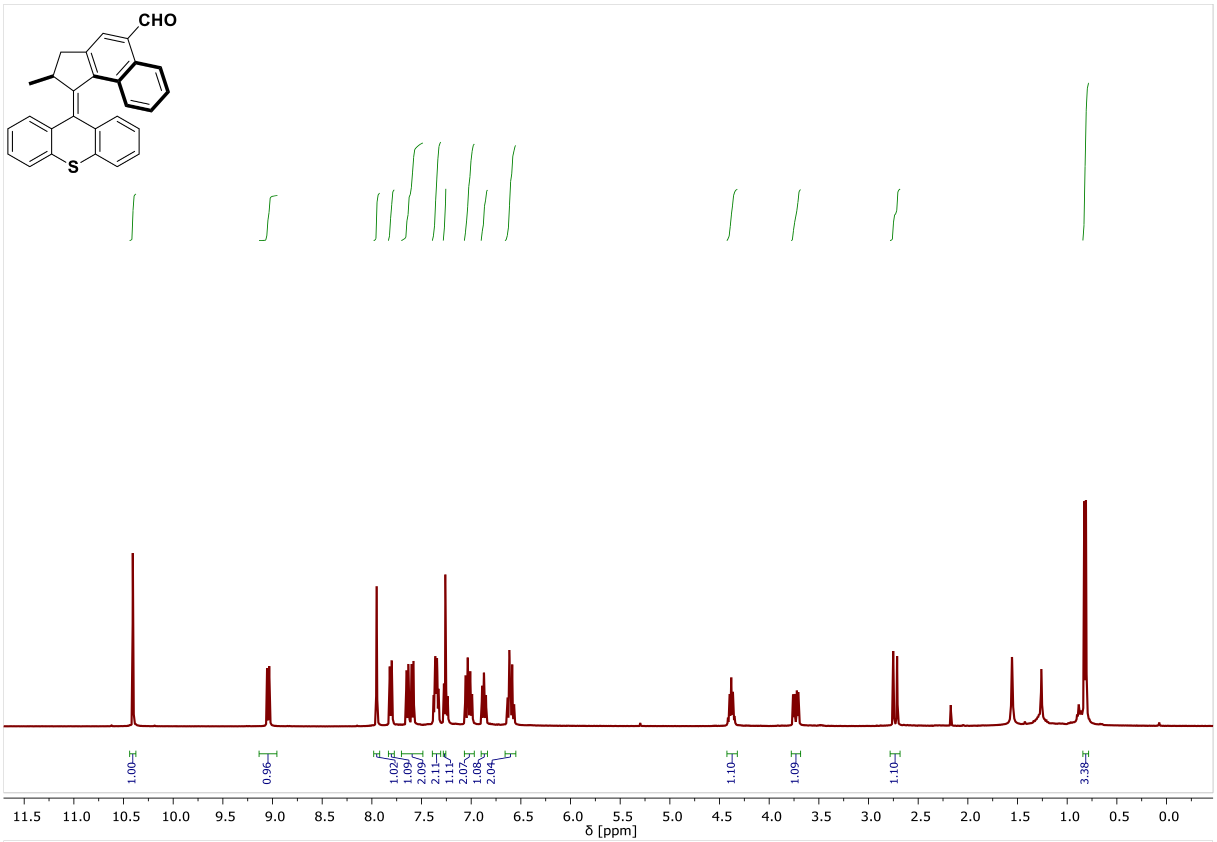


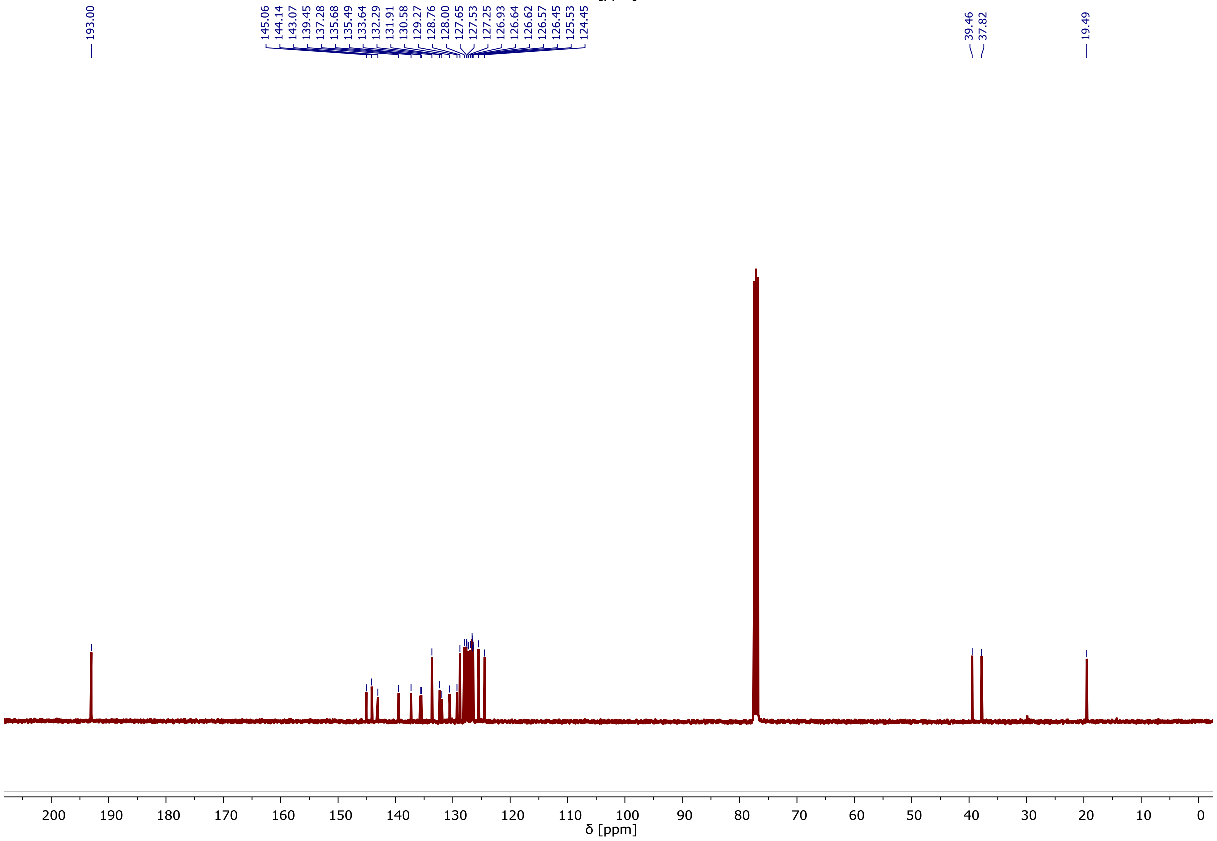


$^{1}$H and $^{13}$C NMR of compound **5.**

**Table S1.** Presentative examples with detailed information for the visible-light-driven molecular motors.

| Motors[a] | Φ st→mst [%] | Φ mst→st [%] | [nm] | PSS (metastable/stable) | Half life $t_{1/2}$ (20 °C) (min) |
|---|---|---|---|---|---|
| **Oxindole motors**[52] | < 3.0 | < 0.7 | 420 | < 70:30 | - |
| **Push-pull 2nd generation motor**[40,43] | 5.8 | 3.1 | 455 | 76:24 | 15.5 ($CH_2Cl_2$) 41.7 ($CHCl_3$) |
| **Pi-extended motor**[36] | - | - | 420 | 76:24 | 93.9 KJ/mol ($CH_2Cl_2$) |
| **Pyrene motor**[37] | 1.4 | 0.38 | 455 | 28:72 | 11.3 ($CH_2Cl_2$) |
| **Hemithioindigo Motor**[51] | 7 | - | 460 | - | - |
| **FRET motor**[42] | 13.4 | - | 455 | 71:29 | 44.3 ($CHCl_3$) |
| **Pd-Porphyrin-motor**[41] | 11 | - | 532 | 33:67 | - |
| **Donor-acceptor motor**[39] | - | - | 435 | 90:10 | 1.7 (50 °C, $CHCl_3$) |
| **Bodipy motor**[44] | 22 (acetone) 12 (toluene) | 51 (acetone) 18 (toluene) | 390 | 96:4 (acetone) 40:60 (toluene) | 2.8 (acetone) 4.0 (THF) 4.8 (toluene) |
| | 3 (acetone) 4 (toluene) | 4 (acetone) 4 (toluene) | 505 | 85:15 (acetone) 30:70 (toluene) | |
| **Triphenylamine motor**[43] | 2.3 | 0.9 | 470 | 80:20 | 6.0 ($CH_2Cl_2$) |

**Table S2.** Molar absorption coefficient of stable and metastable isomers of motors **1**, **2**, **4** at indicated wavelengths.

| Sample | $\varepsilon_{st}$ (365 nm) [mol$^{-1}$·cm$^{-1}$] | $\varepsilon_{mst}$ (365 nm) [mol$^{-1}$·cm$^{-1}$] | $\varepsilon_{st}$ (390 nm) [mol$^{-1}$·cm$^{-1}$] | $\varepsilon_{mst}$ (390 nm) [mol$^{-1}$·cm$^{-1}$] | $PSS_{365}$ (mst : st) | $PSS_{390}$[a] (mst : st) |
|---|---|---|---|---|---|---|
| Motor **1** | 25200 | 9320 | 32310 | 17110 | 83:17 | - |
| Motor **2** | 23250[b] | 13180 | 16490 | 7100 | 80:20[c] | - |
| Motor **4** | 17370 | 10450 | 4390 | 120 | 85:15 | 85:15 |

[a] $PSS_{390}$ was assumed to be negligible difference as $PSS_{405}$ or $PSS_{395}$. [b] $\varepsilon_{st}$ and $\varepsilon_{mst}$ were obtained at 420 nm for motor **2**. [c] PSS obtained at 420 nm irradiation.

**Table S3.** Molar absorption coefficient of stable and metastable isomers of motors **3** at indicated wavelengths.

| Sample | $\varepsilon_{st}$ (365 nm) [mol$^{-1}$·cm$^{-1}$] | $\varepsilon_{mst}$ (365 nm) [mol$^{-1}$·cm$^{-1}$] | $\varepsilon_{st}$ (390 nm) [mol$^{-1}$·cm$^{-1}$] | $\varepsilon_{mst}$ (390 nm) [mol$^{-1}$·cm$^{-1}$] | $\varepsilon_{st}$ (445 nm) [mol$^{-1}$·cm$^{-1}$] | $\varepsilon_{mst}$ (445 nm) (mst : st) |
|---|---|---|---|---|---|---|
| Motor **3** | 18030 | 5890 | 16760 | 11600 | 620 | 17020 |

**Table S4.** Experimental and Calculated $\lambda_{max}$ of Motors 1-5.

| Motor<br>*R*= | $\lambda_{max}^{exp}$ (nm)<br>-CHO | $\lambda_{max}^{calc}$ (nm)[a]<br>-CHO | $\lambda_{max}^{exp}$ (nm)<br>-Br | $\lambda_{max}^{exp}$ (nm)<br>-H |
|---|---|---|---|---|
| **1**$_s$ | 385 | 383 | 365 | 360[56] |
| **2**$_s$ | 415 | 418 | 395 | 390[80] |
| **3**$_s$ | 375 | 378 | 355 | 355[106] |
| **4**$_s$ | 360 | 364 | 320 | - |
| **5**$_s$ | 390 | 383 | 360 | 370[52] |

[a] Calculated $\lambda_{max}$ at the ωB97X-D3/def2-TZVPP//r$^2$SCAN-3c/CPCM($CH_2Cl_2$) level of theory, corrected with a linear correction (see Figure S27).

**Table S5.** Experimental and Calculated Activation Barriers of the Thermal Steps of Motors 1-5 (calculations were performed at a $r^2$SCAN-3c/CPCM($CH_2Cl_2$) level of theory).

| Motor<br>*R*= | $\Delta^{‡}G^{exp}$ [kJ/mol]<br>-CHO | $\Delta^{‡}G^{calc}$ [kJ/mol]<br>-CHO | $\Delta^{‡}G^{exp}$ [kJ/mol]<br>-H |
|---|---|---|---|
| **$1_m$** | 80.0 ± 0.3 | 90.73 | 79.1[56] |
| **$2_m$** | 86.4 ± 0.1 | 93.06 | 85.0[106] |
| **$3_m$** | - | 129.80[a]<br>81.92[b] | 122.2[63] |
| **$4_m$** | 94.2 ± 0.3 | 95.9[c] | 91.6[22] |
| **$5_m$** | - | 38.5 | 33.5[78] |

[a] THI activation barrier of **$3_m$**
[b] TEZI activation barrier of **$3_m$**
[c] Activation barrier of the rate-determining step of the lowest energy pathway.

**Table S6.** Relative energies of motor 1.

| Motor isomer | Relative energy (kJ $mol^{-1}$) |
|---|---|
| **$1_s$** | 0.00 |
| **$1\text{-}TS_{THI}$** | 102.76 |
| **$1_m$** | 12.03 |

**Table S7.** Relative energies of motor 2.

| Motor isomer | Relative energy (kJ $mol^{-1}$) |
|---|---|
| $\mathbf{2_s}$ | 0.00 |
| $\mathbf{2\text{-}TS_{THI}}$ | 105.16 |
| $\mathbf{2_m}$ | 12.10 |

**Table S8. Relative energies of motor 3.**

| Motor isomer | Relative energy (kJ $mol^{-1}$) |
|---|---|
| $\mathbf{3_s}$ | 0.00 |
| $\mathbf{3\text{-}TS_{THI}}$ | 150.81 |
| $\mathbf{3\text{-}TS_{TEZI}}$ | 102.94 |
| $\mathbf{3_m}$ | 21.02 |

**Table S9.** Relative energies of motor 4.

| Motor isomer | Relative energy (kJ mol$^{-1}$) |
|---|---|
| **$4_s$** | 0.00 |
| **4-TS3** | 59.01 |
| **4-I2** | 18.42 |
| **4-TS2** | 123.86 |
| **4-I1** | 46.75 |
| **4-TS1** | 68.29 |
| **$4_m$** | 22.14 |
| **4-TS4** | 73.24 |
| **4-I3** | 51.60 |
| **4-TS5** | 118.08 |

**Table S10.** Relative energies of motor 5.

| Motor isomer | Relative energy (kJ $mol^{-1}$) |
|---|---|
| $\mathbf{5_s}$ | 0.00 |
| $\mathbf{5\text{-}TS_{THI}}$ | 76.22 |
| $\mathbf{5_m}$ | 37.76 |

**Table S11.** Summary of the QY of a 2nd generation motor core with different substitutions.

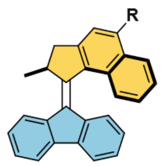


| -R group | PSS at 365 nm | $\Phi_{st \rightarrow mst}$ | $\Phi_{mst \rightarrow st}$ |
|---|---|---|---|
| -H | 25:75 | 0.14 | 0.50 |
| **-CHO** | **20:80** | **0.277** | **0.174** |
| -OMe | 43:57 | 0.048 | 0.17 |
| -Cl | 30:70 | 0.15 | 0.69 |
| -CN | 18:82 | 0.20 | 0.39 |

**Table S12.** Energies relative to the ground-state stable form and characteristic dihedral angle values at stationary structures of Motor 2 (upper table) and its unsubstituted parent molecule (lower table) determined at the TDA TD-DFT CAM-B3LYP/def2-SVP level of theory with the BJ-D3 dispersion correction. For definition of dihedral angles, please refer to Fig. S33. Data in the gray column correspond to unconverged excited state optimization of the unsubstituted motor M form which ended in the CI region.

| **Motor 2 (formylated)** | **S ($S_0$-opt)** | **S* ($S_1$-opt)** | **M ($S_0$-opt)** | **M* ($S_1$-opt)** |
|---|---|---|---|---|
| **E.rel** [$S_1$/$S_0$ gap] (eV) | 0.000 [3.465] | 3.125 [2.739] | 0.192 [3.175] | 3.003 [2.399] |
| φ (deg) | 170 | 155 | 28 | 47 |
| **γ** (deg) | 177 | 176 | 178 | 175 |
| **α** (deg) | 104 | 101 | 34 | 35 |

| **Motor 2 (unsubstituted)** | **S ($S_0$-opt)** | **S* ($S_1$-opt)** | **M ($S_0$-opt)** | **M* ($S_1$-opt)** |
|---|---|---|---|---|
| **E.rel** [$S_1$/$S_0$ gap] (eV) | 0.000 [3.684] | 3.286 [2.741] | 0.183 [3.369] | 2.552 [0.130] |
| φ (deg) | 170 | 149 | 28 | 97 |
| **γ** (deg) | 177 | 176 | 178 | 173 |
| **α** (deg) | 103 | 101 | 34 | 43 |

**Data S1: XYZ coordinates of optimized structures**

Cartesian coordinates of ground-state minima and transition states. Cartesian coordinates of **2** and non-formylated counterpart optimized at the DFT (ground electronic state structures, $S_0$) and TDA TD-DFT level of theory (excited state structures, $S^1$), with CAM-B3LYP functional and def2-SVP basis set, with included BJ-D3 dispersion correction excited state. Cartesian coordinates of non-formylated counterparts of motors studied in the manuscript, optimized at the r2scan-c3 level of theory, with DCM solvent included at the PCM level. Cartesian coordinates of **2** and non-formylated counterpart optimized at the ODM2/MRCI level of theory Meci1/Meci2 are respective structures of minimum-energy conical intersection points.

## REFERENCES AND NOTES


1. K. Kinbara, T. Aida, Toward intelligent molecular machines: Directed motions of biological and artificial molecules and assemblies. *Chem. Rev.* **105**, 1377–1400 (2005).

2. W. R. Browne, B. L. Feringa, Making molecular machines work. *Nat. Nanotechnol.* **1**, 25–35 (2006).

3. V. Balzani, A. Credi, M. Venturi, Light powered molecular machines. *Chem. Soc. Rev.* **38**, 1542–1550 (2009).

4. A. Coskun, M. Banaszak, R. D. Astumian, J. F. Stoddart, B. A. Grzybowski, Great expectations: Can artificial molecular machines deliver on their promise? *Chem. Soc. Rev.* **41**, 19–30 (2012).

5. J.-P. Sauvage, Transition metal-containing rotaxanes and catenanes in motion: Toward molecular machines and motors. *Acc. Chem. Res.* **31**, 611–619 (1998).

6. T. van Leeuwen, A. S. Lubbe, P. Štacko, S. J. Wezenberg, B. L. Feringa, Dynamic control of function by light-driven molecular motors. *Nat. Rev. Chem.* **1**, 0096 (2017).

7. M. Baroncini, S. Silvi, A. Credi, Photo- and redox-driven artificial molecular motors. *Chem. Rev.* **120**, 200–268 (2020).

8. V. Garcia-Lopez, D. Liu, J. M. Tour, Light-activated organic molecular motors and their applications. *Chem. Rev.* **120**, 79–124 (2020).

9. S. Corra, M. Curcio, M. Baroncini, S. Silvi, A. Credi, Photoactivated artificial molecular machines that can perform tasks. *Adv. Mater.* **32**, e1906064 (2020).

10. D. Dattler, G. Fuks, J. Heiser, E. Moulin, A. Perrot, X. Yao, N. Giuseppone, Design of collective motions from synthetic molecular switches, rotors, and motors. *Chem. Rev.* **120**, 310–433 (2020).

11. I. Aprahamian, The future of molecular machines. *ACS Cent. Sci.* **6**, 347–358 (2020).

12. Y. Feng, M. Ovalle, J. S. W. Seale, C. K. Lee, D. J. Kim, R. D. Astumian, J. F. Stoddart, Molecular pumps and motors. *J. Am. Chem. Soc.* **143**, 5569–5591 (2021).

13. J. Sheng, D. R. S. Pooler, B. L. Feringa, Enlightening dynamic functions in molecular systems by intrinsically chiral light-driven molecular motors. *Chem. Soc. Rev.* **52**, 5875–5891 (2023).

14. B. L. Feringa, W. R. Browne. *Molecular Switches* (Wiley-VCH Verlag GmbH & Co. KGaA, 2011).

15. C. J. Bruns, J. F. Stoddart, Rotaxane-based molecular muscles. *Acc. Chem. Res.* **47**, 2186–2199 (2014).

16. S. Kassem, T. van Leeuwen, A. S. Lubbe, M. R. Wilson, B. L. Feringa, D. A. Leigh, Artificial molecular motors. *Chem. Soc. Rev.* **46**, 2592–2621 (2017).

17. D. R. S. Pooler, A. S. Lubbe, S. Crespi, B. L. Feringa, Designing light-driven rotary molecular motors. *Chem. Sci.* **12**, 14964–14986 (2021).

18. S. Krause, B. L. Feringa, Towards artificial molecular factories from framework-embedded molecular machines. *Nat. Rev. Chem.* **4**, 550–562 (2020).

19. D. Roke, S. J. Wezenberg, B. L. Feringa, Molecular rotary motors: Unidirectional motion around double bonds. *Proc. Natl. Acad. Sci. U.S.A.* **115**, 9423–9431 (2018).

20. S. Corra, M. Curcio, A. Credi, Photoactivated artificial molecular motors. *JACS Au* **3**, 1301–1313 (2023).

21. N. Koumura, R. W. Zijlstra, R. A. van Delden, N. Harada, B. L. Feringa, Light-driven monodirectional molecular rotor. *Nature* **401**, 152–155 (1999).

22. N. Koumura, E. M. Geertsema, M. B. van Gelder, A. Meetsma, B. L. Feringa, Second generation light-driven molecular motors. Unidirectional rotation controlled by a single stereogenic center with near-perfect photoequilibria and acceleration of the speed of rotation by structural modification. *J. Am. Chem. Soc.* **124**, 5037–5051 (2002).

23. J. C. Kistemaker, P. Stacko, J. Visser, B. L. Feringa, Unidirectional rotary motion in achiral molecular motors. *Nat. Chem.* **7**, 890–896 (2015).

24. R. Eelkema, M. M. Pollard, J. Vicario, N. Katsonis, B. Serrano Ramon, C. W. M. Bastiaansen, D. J. Broer, B. L. Feringa, Molecular machines: Nanomotor rotates microscale objects. *Nature* **440**, 163 (2006).

25. S. F. Pizzolato, P. Štacko, J. C. M. Kistemaker, T. van Leeuwen, E. Otten, B. L. Feringa, Central-to-helical-to-axial-to-central transfer of chirality with a photoresponsive catalyst. *J. Am. Chem. Soc.* **140**, 17278–17289 (2018).

26. X. Chen, P. J. Gilissen, P. Tinnemans, N. Vanthuyne, F. P. J. T. Rutjes, B. L. Feringa, J. A. A. W. Elemans, R. J. M. Nolte, Enantiodivergent epoxidation of alkenes with a photoswitchable phosphate manganese-salen complex. *Nat. Synth.* **1**, 873–882 (2022).

27. J. Chen, F. K.-C. Leung, M. C. A. Stuart, T. Kajitani, T. Fukushima, E. van der Giessen, B. L. Feringa, Artificial muscle-like function from hierarchical supramolecular assembly of photoresponsive molecular motors. *Nat. Chem.* **10**, 132–138 (2018).

28. S. Chen, L. Yang, F. K.-C. Leung, T. Kajitani, M. C. A. Stuart, T. Fukushima, P. van Rijn, B. L. Feringa, Photoactuating artificial muscles of motor amphiphiles as an extracellular matrix mimetic scaffold for mesenchymal stem cells. *J. Am. Chem. Soc.* **144**, 3543–3553 (2022).

29. W. Danowski, T. van Leeuwen, S. Abdolahzadeh, D. Roke, W. R. Browne, S. J. Wezenberg, B. L. Feringa, Unidirectional rotary motion in a metal-organic framework. *Nat. Nanotechnol.* **14**, 488–494 (2019).

30. W. Danowski, F. Castiglioni, A. S. Sardjan, S. Krause, L. Pfeifer, D. Roke, A. Comotti, W. R. Browne, B. L. Feringa, Visible-light-driven rotation of molecular motors in a dual-function metal-organic framework enabled by energy transfer. *J. Am. Chem. Soc.* **142**, 9048–9056 (2020).

31. Y. Shan, J. Sheng, Q. Zhang, M. C. A. Stuart, D.-H. Qu, B. L. Feringa, Multi-state photoluminescent properties of an overcrowded alkene-based molecular motor in aggregates. *Aggregate* **5**, e584 (2024).

32. Y. Shan, Q. Zhang, J. Sheng, M. C. A. Stuart, D.-H. Qu, B. L. Feringa, Motorized photomodulator: Making a non-photoresponsive supramolecular gel switchable by light. *Angew. Chem. Int. Ed. Engl.* **62**, e202310582 (2023).

33. R. A. van Delden, M. K. J. ter Wiel, M. M. Pollard, J. Vicario, N. Koumura, B. L. Feringa, Unidirectional molecular motor on a gold surface. *Nature* **437**, 1337–1340 (2005).

34. Y. Jiang, W. Danowski, B. L. Feringa, L. Heinke, Nanoporous films with oriented arrays of molecular motors for photoswitching the guest adsorption and diffusion. *Angew. Chem. Int. Ed. Engl.* **62**, e202214202 (2023).

35. H. Wang, H. K. Bisoyi, X. Zhang, F. Hassan, Q. Li, Visible light-driven molecular switches and motors: Recent developments and applications. *Chem. A Eur. J.* **28**, e202103906 (2022).

36. T. van Leeuwen, J. Pol, D. Roke, S. J. Wezenberg, B. L. Feringa, Visible-light excitation of a molecular motor with an extended aromatic core. *Org. Lett.* **19**, 1402–1405 (2017).

37. D. Roke, B. L. Feringa, S. J. Wezenberg, A visible-light-driven molecular motor based on pyrene. *Helv. Chim. Acta* **102**, e1800221 (2019).

38. S. van Vliet, J. Sheng, C. N. Stindt, B. L. Feringa, All-visible-light-driven salicylidene schiff-base-functionalized artificial molecular motors. *Nat. Commun.* **15**, 6461 (2024).

39. R. A. van Delden, N. Koumura, A. Schoevaars, A. Meetsma, B. L. Feringa, A donor-acceptor substituted molecular motor: Unidirectional rotation driven by visible light. *Org. Biomol. Chem.* **1**, 33–35 (2003).

40. L. Pfeifer, M. Scherübl, M. Fellert, W. Danowski, J. Cheng, J. Pol, B. L. Feringa, Photoefficient 2nd generation molecular motors responsive to visible light. *Chem. Sci.* **10**, 8768–8773 (2019).

41. A. Cnossen, L. Hou, M. M. Pollard, P. V. Wesenhagen, W. R. Browne, B. L. Feringa, Driving unidirectional molecular rotary motors with visible light by intra- and intermolecular energy transfer from palladium porphyrin. *J. Am. Chem. Soc.* **134**, 17613–17619 (2012).

42. L. Pfeifer, N. V. Hoang, M. Scherubl, M. S. Pshenichnikov, B. L. Feringa, Powering rotary molecular motors with low-intensity near-infrared light. *Sci. Adv.* **6**, eabb6165 (2020).

43. L. Pfeifer, N. V. Hoang, S. Crespi, M. S. Pshenichnikov, B. L. Feringa, Dual-function artificial molecular motors performing rotation and photoluminescence. *Sci. Adv.* **8**, eadd0410 (2022).

44. R. Toyoda, N. V. Hoang, K. G. Moghaddam, S. Crespi, D. R. S. Pooler, S. Faraji, M. S. Pshenichnikov, B. L. Feringa, Synergistic interplay between photoisomerization and photoluminescence in a light-driven rotary molecular motor. *Nat. Commun.* **13**, 5765 (2022).

45. S. J. Wezenberg, K. Y. Chen, B. L. Feringa, Visible-light-driven photoisomerization and increased rotation speed of a molecular motor acting as a ligand in a ruthenium(II) complex. *Angew. Chem. Int. Ed. Engl.* **54**, 11457–11461 (2015).

46. A. Faulkner, T. van Leeuwen, B. L. Feringa, S. J. Wezenberg, Allosteric regulation of the rotational speed in a light-driven molecular motor. *J. Am. Chem. Soc.* **138**, 13597–13603 (2016).

47. Z. T. Shi, Y.-X. Hu, Z. Hu, Q. Zhang, S.-Y. Chen, M. Chen, J.-J. Yu, G.-Q. Yin, H. Sun, L. Xu, X. Li, B. L. Feringa, H.-B. Yang, H. Tian, D.-H. Qu, Visible-light-driven rotation of molecular motors in discrete supramolecular metallacycles. *J. Am. Chem. Soc.* **143**, 442–452 (2021).

48. C. N. Stindt, S. Crespi, R. Toyoda, M. F. Hilbers, J. Kemmink, P. van der Meulen, W. J. Buma, B. L. Feringa, Activating a light-driven molecular motor by metal complexation. *Chem* **9**, 2337–2348 (2023).

49. M. Guentner, M. Schildhauer, S. Thumser, P. Mayer, D. Stephenson, P. J. Mayer, H. Dube, Sunlight-powered kHz rotation of a hemithioindigo-based molecular motor. *Nat. Commun.* **6**, 8406 (2015).

50. L. A. Huber, M. Schildhauer, F. Rott, L. A. Huber, M. Guentner, S. Thumser, K. Hoffmann, S. Oesterling, R. de Vivie-Riedle, E. Riedle, H. Dube, Direct observation of hemithioindigo-motor unidirectionality. *Angew. Chem. Int. Ed. Engl.* **56**, 14536–14539 (2017).

51. R. Wilcken, M. Schildhauer, F. Rott, L. A. Huber, M. Guentner, S. Thumser, K. Hoffmann, S. Oesterling, R. de Vivie-Riedle, E. Riedle, H. Dube, Complete mechanism of hemithioindigo motor rotation. *J. Am. Chem. Soc.* **140**, 5311–5318 (2018).

52. D. Roke, M. Sen, W. Danowski, S. J. Wezenberg, B. L. Feringa, Visible-light-driven tunable molecular motors based on oxindole. *J. Am. Chem. Soc.* **141**, 7622–7627 (2019).

53. K. Kuntze, D. R. S. Pooler, M. Di Donato, M. F. Hilbers, P. van der Meulen, W. J. Buma, A. Priimagi, B. L. Feringa, S. Crespi, A visible-light-driven molecular motor based on barbituric acid. *Chem. Sci.* **14**, 8458–8465 (2023).

54. J. Sheng, W. Danowski, A. S. Sardjan, J. Hou, S. Crespi, A. Ryabchun, M. Paradiz Domínguez, W. Jan Buma, W. R. Browne, B. L. Feringa, Formylation boosts the performance of light-driven overcrowded alkene-derived rotary molecular motors. *Nat. Chem.* **16**, 1330–1338 (2024).

55. M. K. ter Wiel, J. Vicario, S. G. Davey, A. Meetsma, B. L. Feringa, New procedure for the preparation of highly sterically hindered alkenes using a hypervalent iodine reagent. *Org. Biomol. Chem.* **3**, 28–30 (2005).

56. M. M. Pollard, A. Meetsma, B. L. Feringa, A redesign of light-driven rotary molecular motors. *Org. Biomol. Chem.* **6**, 507–512 (2008).

57. J. Chen, S. J. Wezenberg, B. L. Feringa, Intramolecular transport of small-molecule cargo in a nanoscale device operated by light. *Chem. Commun.* **52**, 6765–6768 (2016).

58. J. Hou, A. Mondal, G. Long, L. de Haan, W. Zhao, G. Zhou, D. Liu, D. J. Broer, J. Chen, B. L. Feringa, Photo-responsive helical motion by light-driven molecular motors in a liquid-crystal network. *Angew. Chem. Int. Ed. Engl.* **60**, 8251–8257 (2021).

59. J. Hou, G. Long, W. Zhao, G. Zhou, D. Liu, D. J. Broer, B. L. Feringa, J. Chen, Phototriggered complex motion by programmable construction of light-driven molecular motors in liquid crystal networks. *J. Am. Chem. Soc.* **144**, 6851–6860 (2022).

60. G. T. Carroll, G. London, T. F. Landaluce, P. Rudolf, B. L. Feringa, Adhesion of photon-driven molecular motors to surfaces via 1,3-dipolar cycloadditions: Effect of interfacial interactions on molecular motion. *ACS Nano* **5**, 622–630 (2011).

61. J. Bao, R. Lan, C. Shen, R. Huang, Z. Wang, W. Hu, L. Zhang, H. Yang, Modulation of chirality and intensity of circularly polarized luminescence emitting from cholesteric liquid crystals triggered by photoresponsive molecular motor. *Adv. Opt. Mater.* **10**, 2101910 (2021).

62. D. Pijper, B. L. Feringa, Molecular transmission: Controlling the twist sense of a helical polymer with a single light-driven molecular motor. *Angew. Chem. Int. Ed. Engl.* **46**, 3693–3696 (2007).

63. J. Sheng, W. Danowski, S. Crespi, A. Guinart, X. Chen, C. Stähler, B. L. Feringa, Designing P-type bi-stable overcrowded alkene-based chiroptical photoswitches. *Chem. Sci.* **14**, 4328–4336 (2023).

64. F. Castiglioni, W. Danowski, J. Perego, F. K.-C. Leung, P. Sozzani, S. Bracco, S. J. Wezenberg, A. Comotti, B. L. Feringa, Modulation of porosity in a solid material enabled by bulk photoisomerization of an overcrowded alkene. *Nat. Chem.* **12**, 595–602 (2020).

65. J. Hou, R. Toyoda, S. C. J. Meskers, B. L. Feringa, Programming and dynamic control of the circular polarization of luminescence from an achiral fluorescent dye in a liquid crystal host by molecular motors. *Angew. Chem. Int. Ed. Engl.* **61**, e202206310 (2022).

66. J. Sheng, J. Perego, W. Danowski, S. Bracco, S. Chen, X. Zhu, C. X. Bezuidenhout, S. Krause, W. R. Browne, P. Sozzani, A. Comotti, B. L. Feringa, Construction of a three-state responsive framework from a bistable photoswitch. *Chem* **9**, 2701–2716 (2023).

67. D. Bleger, S. Hecht, Visible-light-activated molecular switches. *Angew. Chem. Int. Ed. Engl.* **54**, 11338–11349 (2015).

68. Z. Zhang, W. Wang, M. O'Hagan, J. Dai, J. Zhang, H. Tian, Stepping out of the blue: From visible to near-IR triggered photoswitches. *Angew. Chem. Int. Ed. Engl.* **61**, e202205758 (2022).

69. D. Villaron, N. Duindam, S. J. Wezenberg, Push-pull stiff-stilbene: Proton-gated visible-light photoswitching and acid-catalyzed isomerization. *Chem. A Eur. J.* **27**, 17346–17350 (2021).

70. F. Xu, J. Sheng, C. N. Stindt, S. Crespi, W. Danowski, M. F. Hilbers, W. Jan Buma, B. L. Feringa, All-visible-light-driven stiff-stilbene photoswitches. *Chem. Sci.* **15**, 6763–6769 (2024).

71. A. A. Beharry, O. Sadovski, G. A. Woolley, Azobenzene photoswitching without ultraviolet light. *J. Am. Chem. Soc.* **133**, 19684–19687 (2011).

72. D. Bleger, J. Schwarz, A. M. Brouwer, S. Hecht, *o*-Fluoroazobenzenes as readily synthesized photoswitches offering nearly quantitative two-way isomerization with visible light. *J. Am. Chem. Soc.* **134**, 20597–20600 (2012).

73. L. N. Lameijer, S. Budzak, N. A. Simeth, M. J. Hansen, B. L. Feringa, D. Jacquemin, W. Szymanski, General principles for the design of visible-light-responsive photoswitches: Tetra-ortho-chloro-azobenzenes. *Angew. Chem. Int. Ed. Engl.* **59**, 21663–21670 (2020).

74. J. Sheng, J. Perego, S. Bracco, P. Cieciórski, W. Danowski, A. Comotti, B. L. Feringa, Orthogonal photoswitching in a porous organic framework. *Angew. Chem. Int. Ed. Engl.* **63**, e202404878 (2024).

75. V. Garcia-Lopez, F. Chen, L. G. Nilewski, G. Duret, A. Aliyan, A. B. Kolomeisky, J. T. Robinson, G. Wang, R. Pal, J. M. Tour, Molecular machines open cell membranes. *Nature* **548**, 567–572 (2017).

76. T. Galbadage, D. Liu, L. B. Alemany, R. Pal, J. M. Tour, R. S. Gunasekera, J. D. Cirillo, Molecular nanomachines disrupt bacterial cell wall, increasing sensitivity of extensively drug-resistant klebsiella pneumoniae to meropenem. *ACS Nano* **13**, 14377–14387 (2019).

77. A. L. Santos, D. Liu, A. K. Reed, A. M. Wyderka, A. van Venrooy, J. T. Li, V. D. Li, M. Misiura, O. Samoylova, J. L. Beckham, C. Ayala-Orozco, A. B. Kolomeisky, L. B. Alemany, A. Oliver, G. P. Tegos, J. M. Tour, Light-activated molecular machines are fast-acting broad-spectrum antibacterials that target the membrane. *Sci. Adv.* **8**, eabm2055 (2022).

78. M. Klok, N. Boyle, M. T. Pryce, A. Meetsma, W. R. Browne, B. L. Feringa, MHz unidirectional rotation of molecular rotary motors. *J. Am. Chem. Soc.* **130**, 10484–10485 (2008).

79. J. C. Kistemaker, S. F. Pizzolato, T. van Leeuwen, T. C. Pijper, B. L. Feringa, Spectroscopic and theoretical identification of two thermal isomerization pathways for bistable chiral overcrowded alkenes. *Chem. A Eur. J.* **22**, 13478–13487 (2016).

80. J. Conyard, A. Cnossen, W. R. Browne, B. L. Feringa, S. R. Meech, Chemically optimizing operational efficiency of molecular rotary motors. *J. Am. Chem. Soc.* **136**, 9692–9700 (2014).

81. R. Wilcken, A. Gerwien, L. A. Huber, H. Dube, E. Riedle, Quantitative in-situ NMR illumination for excitation and kinetic analysis of molecular motor intermediates. *ChemPhotoChem* **6**, e202100232 (2022).

82. P. Roy, A. S. Sardjan, W. R. Browne, B. L. Feringa, S. R. Meech, Excited state dynamics in unidirectional photochemical molecular motors. *J. Am. Chem. Soc.* **146**, 12255–12270 (2024).

83. T. M. Neubauer, T. van Leeuwen, D. Zhao, A. S. Lubbe, J. C. Kistemaker, B. L. Feringa, Asymmetric synthesis of first generation molecular motors. *Org. Lett.* **16**, 4220–4223 (2014).

84. C. Ayala Orozco, D. Liu, Y. Li, L. B. Alemany, R. Pal, S. Krishnan, J. M. Tour, Visible-light-activated molecular nanomachines kill pancreatic cancer cells. *ACS Appl. Mater. Interfaces* **12**, 410–417 (2020).

85. H. J. Kuhn, S. E. Braslavsky, R. Schmidt, Chemical actinometry (IUPAC Technical Report). *Pure Appl. Chem.* **76**, 2105–2146 (2004).

86. K. Stranius, K. Borjesson, Determining the photoisomerization quantum yield of photoswitchable molecules in solution and in the solid state. *Sci. Rep.* **7**, 41145 (2017).

87. M. Montalti, A. Credi, L. Prodi, M. T. Gandolfi, Handbook of Photochemistry (CRC Press, ed. 3, 2006); https://doi.org/10.1201/9781420015195.

88. S. Hoops, S. Sahle, R. Gauges, C. Lee, J. Pahle, N. Simus, M. Singhal, L. Xu, P. Mendes, U. Kummer, COPASI—A COmplex PAthway SImulator. *Bioinformatics* **22**, 3067–3074 (2006).

89. J. J. Snellenburg, S. P. Laptenok, R. Seger, K. M. Mullen, I. H. M. van Stokkum, Glotaran: A java-based graphical user interface for the R package TIMP. *J. Stat. Softw.* **49**, 1–22 (2012).

90. L. Krause, R. Herbst-Irmer, G. M. Sheldrick, D. Stalke, D., Comparison of silver and molybdenum microfocus x-ray sources for single-crystal structure determination. *J. Appl. Cryst.* **48**, 3–10 (2015).

91. G. M. Sheldrick, SHELXT–Integrated space-group and crystal-structure determination. *Acta Crystallogr. A. Found Adv.* **71**, 3–8 (2015).

92. G. M. Sheldrick, Crystal structure refinement with SHELXL. *Acta Crystallogr. C. Struct. Chem.* **71**, 3–8 (2015).

93. O. V. Dolomanov, L. J. Bourhis, R. J. Gildea, J. A. K. Howard, H. Puschmann, OLEX2: A complete structure solution, refinement and analysis program. *J. Appl. Cryst.* **42**, 339–341 (2009).

94. S. Grimme, A. Hansen, S. Ehlert, J. M. Mewes, r(2)SCAN-3c: A “Swiss army knife” composite electronic-structure method. *J. Chem. Phys.* **154**, 064103 (2021).

95. F. Neese, F. Wennmohs, U. Becker, C. Riplinger, The ORCA quantum chemistry program package. *J. Chem. Phys.* **152**, 224108 (2020).

96. V. Barone, M. Cossi, Quantum calculation of molecular energies and energy gradients in solution by a conductor solvent model. *J. Phys. Chem. A* **102**, 1995–2001 (1998).

97. F. Weigend, R. Ahlrichs, Balanced basis sets of split valence, triple zeta valence and quadruple zeta valence quality for H to Rn: Design and assessment of accuracy. *Phys. Chem. Chem. Phys.* **7**, 3297–3305 (2005).

98. J. D. Chai, M. Head-Gordon, Long-range corrected hybrid density functionals with damped atom-atom dispersion corrections. *Phys. Chem. Chem. Phys.* **10**, 6615–6620 (2008).

99. S. Grimme, J. Antony, S. Ehrlich, H. Krieg, A consistent and accurate ab initio parametrization of density functional dispersion correction (DFT-D) for the 94 elements H-Pu. *J. Chem. Phys.* **132**, 154104 (2010).

100. C. Stahler, D. R. S. Pooler, R. Costil, D. Sudan, P. van der Meulen, R. Toyoda, B. L. Feringa, Coupled rocking motion in a light-driven rotary molecular motor. *J. Org. Chem.* **89**, 1–8 (2024).

101. Gaussian 16, Revision C.01, M. J. Frisch, G. W. Trucks, H. B. Schlegel, G. E. Scuseria, M. A. Robb, J. R. Cheeseman, G. Scalmani, V. Barone, G. A. Petersson, H. Nakatsuji, X. Li, M. Caricato, A. V. Marenich, J. Bloino, B. G. Janesko, R. Gomperts, B. Mennucci, H. P. Hratchian, J. V. Ortiz, A. F. Izmaylov, J. L. Sonnenberg, D. Williams-Young, F. Ding, F. Lipparini, F. Egidi, J. Goings, B. Peng, A. Petrone, T. Henderson, D. Ranasinghe, V. G. Zakrzewski, J. Gao, N. Rega, G. Zheng, W. Liang, M. Hada, M. Ehara, K. Toyota, R. Fukuda, J. Hasegawa, M. Ishida, T. Nakajima, Y. Honda, O. Kitao, H. Nakai, T. Vreven, K. Throssell, J. A. Montgomery, Jr., J. E. Peralta, F. Ogliaro, M. J. Bearpark, J. J. Heyd, E. N. Brothers, K. N. Kudin, V. N. Staroverov, T. A. Keith, R. Kobayashi, J. Normand, K. Raghavachari, A. P. Rendell, J. C. Burant, S. S. Iyengar, J. Tomasi, M. Cossi, J. M. Millam, M. Klene, C. Adamo, R. Cammi, J. W. Ochterski, R. L. Martin, K. Morokuma, O. Farkas, J. B. Foresman, and D. J. Fox, Gaussian, Inc., Wallingford CT, 2016.

102. T. Yanai, D. P. Tew, N. C. Handy, A new hybrid exchange–correlation functional using the Coulomb-attenuating method (CAM-B3LYP). *Chem. Phys. Lett.* **393**, 51–57 (2004).

103. S. Grimme, S. Ehrlich, L. Goerigk, Effect of the damping function in dispersion corrected density functional theory. *J. Comput. Chem.* **32**, 1456–1465 (2011).

104. P. O. Dral, X. Wu, W. Thiel, Semiempirical quantum-chemical methods with orthogonalization and dispersion corrections. *J. Chem. Theory Comput.* **15**, 1743–1760 (2019).

105. M. W. Thiel, Max-Planck-Institut für Kohlenforschung, Mülheim, Germany (2020); https://mndo.kofo.mpg.de.

106. J. Vicario, A. Meetsma, B. L. Feringa, Controlling the speed of rotation in molecular motors. Dramatic acceleration of the rotary motion by structural modification. *Chem. Commun.* 5910–5912 (2005); https://doi.org/10.1039/B507264F.
